# Bovine infectious abortion: a systematic review and meta-analysis

**DOI:** 10.3389/fvets.2023.1249410

**Published:** 2023-09-29

**Authors:** Yanina Paola Hecker, Sara González-Ortega, Santiago Cano, Luis Miguel Ortega-Mora, Pilar Horcajo

**Affiliations:** ^1^SALUVET, Animal Health Department, Faculty of Veterinary Sciences, Complutense University of Madrid, Madrid, Spain; ^2^Institute of Innovation for Agricultural Production and Sustainable Development (IPADS Balcarce), INTA-CONICET, Balcarce, Argentina; ^3^Computing Services, Research Support Center, Complutense University of Madrid, Madrid, Spain

**Keywords:** bovine abortion, infectious agents, prevalence, diagnosis, meta-analysis

## Abstract

The aim of the present systematic review and meta-analysis was to identify the main infectious agents related to bovine abortion worldwide in the period between 2000 and 2022. First, we investigated the global prevalence of infectious agents related to bovine abortion. For this analysis, only 27 articles detected of a wide panel of agents were included. The random effects model revealed that the estimated prevalence of the abortifacient agents in bovine abortion was 45.7%. The heterogeneity among studies was high, but Egger’s test showed that there was no publication bias, even though the total number of samples analyzed in these articles was variable. There was no significant effect of the year of the study publication on the estimated prevalence, although an increasing trend was observed over time, possibly due to the implementation of new diagnostic techniques. Then, we analyzed the prevalence of the main transmissible agents in bovine abortion. For this analysis, 76 studies that analyzed 19,070 cases were included. Some infectious agent was detected in 7,319 specimens, and a final diagnosis was reached in 3,977 of these, when both the infectious agent and compatible histopathological changes were detected. We found that *Neospora caninum* was the most detected agent (22.2%), followed by opportunistic bacteria (21.4%), Chlamydiaceae family (10.9%) and *Coxiella burnetii* (9.5%). Regarding viral agents, bovine herpes virus type 1 and bovine viral diarrhea displayed similar prevalence rates (approximately 5%). After considering the description of specific histopathological changes, our analyzes showed that *N. caninum* was a confirmed cause of abortion in 16.7% of the analyzed cases, followed by opportunistic bacteria (12.6%) and *Chlamydia* spp. (6.8%); however, *C. burnetii was* only confirmed as a cause of abortion in 1.1% of the cases. For all agents, the heterogeneity among studies was high, and the subgroup analyzes discarded the diagnostic method as the cause of such heterogeneity. This study provides knowledge about the global prevalence of the different infectious agents related to bovine abortion, the most coming of which is *N. caninum*. In addition, this review reveals the existing deficiencies in the diagnosis of bovine abortion that must be addressed in the future.

## 1. Introduction

The cattle industry is the main producer of milk worldwide and the third largest producer of meat, following poultry and pigs (1). Intensive production systems are common in dairy cattle, while extensive grazing is common in beef cattle (2). The main challenge to reaching profitability for both production systems is achieving the largest quantity of calves per bred cow in a year. However, the efficiency in most livestock systems often falls below the level of expectation due to bovine reproductive failure (3). Economic losses induced by bovine abortion and perinatal mortality are associated with costs of lost milk and beef production due to longer calving intervals, decreased average weight in calves, loss of offspring from aborted cows, costs of replacement cows, costs due to losing genetic improvement, and veterinarian costs associated with sanitary and reproductive treatments (4, 5). In addition, these losses lead to animal welfare and societal concerns (6).

Reproductive failure is common in the livestock industry; the prevalences of abortion and perinatal mortality range from 5 to 12% and from 2 to 5%, respectively (5, 7). Bovine abortion and perinatal mortality have multifactorial origins, and many factors can influence viability, such as hormonal fluctuations, genetic abnormalities, and exposure to pharmacologic, environmental, toxic, or infectious agents. Among them, infectious agents play an important role. In addition, many of the agents responsible for bovine reproductive failure have zoonotic potential, such as *Brucella abortus*, *Campylobacter* spp., *Listeria monocytogenes* and *Coxiella burnetii* (8). Consequently, these infectious agents are of importance in both public health and the livestock industry, and strategies to reduce reproductive infections should be based on close collaboration between both medics and veterinarians in accordance with the One Health approach (9). However, the aetiological diagnosis efficacy of these reproductive losses is still below 50% and does not appear to have improved over time despite the development of new diagnostic techniques (4, 6, 7, 10). Recently, a meta-analysis quantified reproductive losses during early pregnancy in beef cattle and the main associated factors (3). However, no updated information is available on the global situation of the main causes of the bovine infectious abortion. Therefore, the aim of the present study was to carry out a systematic review of the scientific literature and a meta-analysis of the prevalence of the main infectious agents involved in bovine abortion worldwide between 2000 and 2022.

## 2. Materials and methods

### 2.1. Search strategy and selection criteria

This review was performed in accordance with the Preferred Reporting Items for Systematic Reviews and Meta-analyzes (PRISMA) guidelines (PRISMA Transparent Reporting of Systematic Reviews and Meta-Analysis) (11). We searched the PubMed^1^ and Scopus^2^ databases from January 1, 2000, to December 31, 2022 to retrieve relevant studies. Search terms included a combination of words “bovine” or “cattle” and “abortion.”

A series of inclusion and exclusion criteria were established, and the reference lists of all included articles were manually searched for potentially eligible literature. The inclusion criteria were as follows: 1) full-text articles available online in English or Spanish languages, 2) studies examining infectious causes of abortion, 3) studies examining at least one infectious agent as a possible foetal death cause, 4) beginning and end date of study implementation should be mentioned, and 5) number of abortion cases in the study is specified. On the other hand, all the studies that did not include the above-mentioned criteria, review articles, systematic analyzes, and experimental infection studies were excluded. In addition, seroprevalence studies in which only serological tests were performed or studies of an outbreak case were also excluded. Furthermore, duplicates and articles that did not include in the title the words “bovine,” “cattle” or “ruminant,” and “abortion/aborted” or “reproductive failure” were eliminated. Then, the abstracts of the remaining studies were screened. Subsequently, the full texts of the potentially eligible studies were screened against the inclusion criteria.

### 2.2. Data extraction

Full text articles were examined and assessed for eligibility by two independent researchers (YPH and SGO). Discrepancies between the authors at any stage of the selection process were resolved by consulting a third and fourth reviewer (PHI, LMOM). Relevant data were extracted from text and placed into purpose-built tables using Microsoft Excel 2019 (Microsoft Corp., Redmond, WA, United States). The required information was extracted for each study, including name of the first author, country of the study, publication year, abortion moment (1st, 2nd, or 3rd trimester); type of production system (dairy or beef); number of analyzed farms; cattle management system (intensive or extensive); history of reproductive failures; type of samples analyzed and sample size (the number of examined animals), infectious pathogens analyzed, diagnostic methods and results, final diagnosis; and any additional data that could be of interest for the study.

### 2.3. Quality assessment

In the present study, the quality of articles was assessed using a modified version of the Newcastle–Ottawa Scale (NOS) (12). This quality scale ranges from 0 to 9 points, and higher scores indicate better quality studies. Only articles with acceptable quality (≥4) were included in this study.

### 2.4. Data synthesis and statistical analysis

All analyzes were carried out using the “SPSS v. 28” and “Open Meta-Analyst 10.12” programs. First, an analysis of the global prevalence of infectious agents related to bovine abortion was performed. All studies that diagnosed a wide panel of agents (viruses, bacteria, protozoa and/or fungi) were included in this analysis. Using the website http://mapinseconds.com, a map that represents the number of cases analyzed in the different countries was created. Second, a meta-analysis was carried out to determine the prevalence of the main infectious agent in bovine abortion. In all cases a random effects model, since high heterogeneity between studies was expected, and confidence levels of 95% were applied.

The degree of heterogeneity between the analyzed studies was determined using the I^2^ statistic. Heterogeneity was considered low if the I^2^ value was less than 25%; average heterogeneity was indicated by an I^2^ value of approximately 50%, and high heterogeneity was indicated by an I^2^ value above 75% (13). Forest plots were used to show the results of each study and the heterogeneity among studies. For the analysis of the prevalence of infectious agents related to bovine abortion, due to the high rate of heterogeneity obtained, sensitivity analysis was performed using the LOOCV (leave-one-out cross-validation) method. This was performed to exclude small studies with extreme effect sizes that could affect the overall results. To determine if publication bias could affect the estimated prevalence, a funnel plot and Egger’s test were used.

To determine the influence of the diagnostic method on the high heterogeneity, subgroup analyzes were performed, and significant differences between groups were analyzed by using a chi-square test. *p* values <0.05 indicated statistical significance and the existence of a relationship between the diagnostic method used and the prevalence obtained (13). Sensitivity analysis was also performed using the LOOCV (leave-one-out cross-validation) method. Finally, we performed a meta-regression to evaluate the effect of the publication year of studies on the prevalence estimates by adding the publication year as a variable to the simple model regression.

## 3. Results

### 3.1. Identification and selection of studies

**Figure 1:**
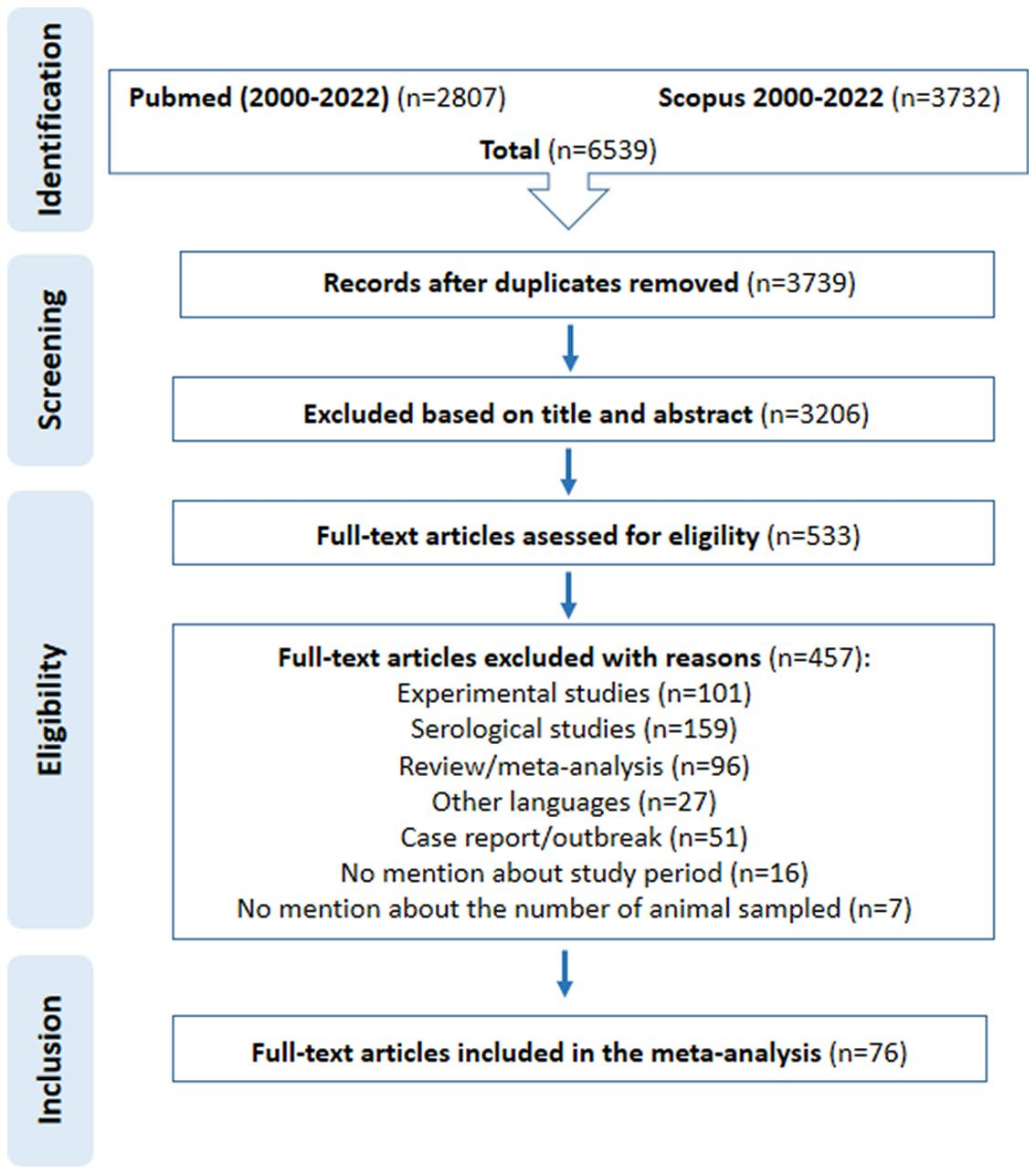
Flow diagram shows the procedure for the selection of the eligible studies.

The search of two databases (PubMed and Scopus) yielded 6,539 articles; 3,739 articles remained after removing duplicates. Following an initial screening based on titles and abstracts, 3,206 studies were excluded because they did not meet the inclusion criteria. Next, the remaining 533 full-text articles were assessed for eligibility. Ultimately, 76 of these articles met the inclusion criteria and were included in the meta-analysis (Figure 1). Information and characteristics of the included articles are presented in Table 1 (4, 7, 14–87). The quality assessment of studies with the NOS checklist indicated that the articles included in this meta-analysis were of acceptable quality (≥4 for each study) (Supplementary Table S1).

**Table 1:** Summary of abstracted datasets from included studies.

Reference	Year	Country	Analysed agents	Abortion stage	Type of herd production system	Total aborted foetal samples	Positives samples	Detected agents
Schock et al.	2000	Scotland	Protozoa	NA	NA	324	39	*N. caninum*
Corbellini et al.	2002	Brazil	Protozoa	2nd trimester (average 5 months)	D, B	46	18	*N. caninum*
Kim et al.	2002	Republic of Korea	Virus bacteria protozoa	NA	NA	180	73	BoHV-1, BVDV, *N. caninum,* other pathogens
Campero et al.	2003	Argentina	Virus bacteria protozoa fungi	1st-2nd-3rd trimester (average 7.1 months)	D, B	354	122	BoHV-1, BVDV, *Brucella* spp., *Campylobacter* spp., *N. caninum*, *Aspergillus* spp., other pathogens
Pereira-Bueno et al.	2003	Spain	Protozoa	2nd–3rd trimester (average 5.9 months)	D, B	31	80	*N. caninum*
Khodakaram-Tafti et al.	2005	Canada	Virus bacteria protozoa fungi	NA	NA	234	92	BoHV-1, BVDV, *Campylobacter* spp., *Listeria* spp., Chlamydiaceae family, *N. caninum,* other pathogens
Takiuchi et al.	2005	Brazil	Virus	2nd-3rd trimester	D, B	55	14	BoHV-1
Corbellini et al.	2006	Brazil	Virus bacteria protozoa fungi	2nd trimester	NA	161	73	BVDV, *Leptospira* spp., *Brucella* spp., *N. caninum*, *Aspergillus* spp., other pathogens
Deim et al.	2006	Hungary	Virus bacteria protozoa fungi	NA	NA	33	22	BoHV-1, BoHV-4
Medina et al.	2006	Mexico	Protozoa	1st-2nd-3rd trimester (average 5.6 months)	D	44	35	*N. caninum*
Parisi et al.	2006	Italy	Bacteria protozoa	NA	D	138	26	*Coxiella burnetii*
Anderson	2007	USA	Virus bacteria protozoa fungi	2nd-3rd trimester	NA	2,296	1,019	BoHV-1, BVDV, *Brucella* spp., *Campylobacter* spp., *Listeria* spp., *N. caninum*, other pathogens
Borel et al.	2007	Swiss	Bacteria	3rd trimester	NA	235	61	Chlamydiaceae family
Deim et al.	2007	Hungary	Virus bacteria protozoa	NA	D	24	7	BoHV-1, BoHV-4
Pescador et al.	2007	Brazil	Protozoa	2nd- 3rd trimeste (average 4.5 months)	NA	258	55	*N. caninum*
Razmi et al.	2007	Iran	Protozoa	NA	D	100	13	*N. caninum*
Reitt et al.	2007	Swiss	Virus bacteria protozoa fungi	2nd- 3rd trimeste (average 7 months)	NA	235	99	BVDV, *C. burnetii*, *Leptospira* spp., Chlamydiaceae family, *N. caninum,* other pathogens
Sadrebazzaz et al.	2007	Iran	Protozoa	2nd-3rd trimester	D	12	5	*N. caninum*
Syrjälä et al.	2007	Finland	Virus bacteria fungi	NA	NA	286	149	*Listeria* spp., other pathogens
Da Silva et al.	2009	Brazil	Virus bacteria protozoa	NA	NA	42	8	*Brucella* spp., *Listeria* sp., *N. caninum*
Yao et al.	2009	China	Protozoa	1st-2nd- 3rd trimester	D	26	15	*N. caninum*
Gagnon et al.	2010	Canada	Virus bacteria protozoa	NA	NA	26	5	BoHV-1, *C. burnetii*, *N. caninum*, other pathogens
Wheelhouse et al.	2010	United Kingdom	Bacteria	NA	NA	83	22	Chlamydiaceae family
Blumer et al.	2011	Swiss	Virus bacteria protozoa fungi	NA	NA	343	110	BVDV, *C. burnetii*, Chlamydiaceae family, *N. caninum*, fungus
Cantas et al.	2011	Cyprus	Bacteria	NA	D	51	18	*C. burnetii*
Clemente et al.	2011	Portugal	Bacteria	NA	NA	43	19	Chlamydiaceae family
dos Santos et al.	2011	Brazil	Protozoa	NA	D	16	6	*N. caninum*
Ghalmi et al.	2011	Algeria	Protozoa	NA	NA	5	3	*N. caninum*
Mazuz et al.	2011	Israel	Protozoa	NA	NA	98	41	*N. caninum*
Safarpoor Dehkordi et al	2011	Iran	Virus	NA	NA	620	127	Pestivirus (BVDV)
Albayrak et al.	2012	Turkey	Virus	NA	NA	21	6	Pestivirus (BVDV)
Crook et al	2012	United Kingdom	Virus	NA	NA	400	10	BoHV-1
Momtaz and Moshkelani	2012	Iran	Bacteria	NA	D	220	46	*Leptospira* spp.
Muskens et al.	2012	Netherlands	Virus bacteria protozoa fungi	1st-2nd- 3rd trimester	D	100	39	BoHV-1, BVDV, *C. burnetii*, *N. caninum*, other pathogens
Safarpoor Dehkordi et al.	2012	Iran	Fungi	NA	NA	350	62	*Aspergillus* spp.
Wheelhouse et al.	2012	Scotland	Virus bacteria fungi	NA	NA	113	31	Chlamydiaceae family
Yang et al.	2012	China	Virus bacteria protozoa	NA	NA	80	45	BoHV-1, BVDV, *Brucella* spp., *N. caninum*, *Tritrichomonas foetus*, other pathogens
Guven et al.	2013	Turkey	Protozoa	2nd-3rd trimester	NA	246	14	*Tritrichomonas foetus*
Safarpoor Dehkordi et al.	2013	Iran	Virus	NA	NA	143	21	BoHV-1
Šteingolde et al.	2014	Latvia	Bacteria	2nd-3rd trimester	NA	186	44	*Listeria* spp.
Kamali et al.	2014	Iran	Protozoa	1st-2nd- 3rd trimester	D	395	179	*N. caninum*
Headley et al.	2015	Brazil	Virus bacteria protozoa	1st-2nd- 3rd trimester	D	14	14	BoHV-1, BVDV, *Brucella* spp., *N. caninum*, other pathogens
Kreizinger et al.	2015	Hungary	Bacteria fungi	NA	NA	85	33	Chlamydiaceae family, *C. burnetti*
Clothier and Anderson	2016	USA	Virus bacteria protozoa fungi	1st-2nd- 3rd trimester	NA	709	335	BoHV-1, BVDV, *Listeria* spp., *Leptospira* spp., *Campylobacter* spp., *C. burnetii*, *N. caninum*, other pathogens
Cvetojević et al.	2016	Serbia	Virus	1st-2nd- 3rd trimester	D	100	21	BoHV-4, BVDV, *N. caninum*
Medina-Esparza et al.	2016	Mexico	Protozoa	NA	D	63	27	*N. caninum*
Pessoa et al.	2016	Brazil	Virus bacteria protozoa fungi	NA	D	38	17	*Leptospira* spp., *N. caninum*, *Aspergillus* spp., other pathogens
Barati et al.	2017	Iran	Bacteria	3rd trimester	NA	9	1	Chlamydiaciae family
Delooz et al.	2017	Belgium	Virus bacteria protozoa fungi	3rd trimester	NA	368	209	BoHV-4, BVDV, *Leptospira* spp., *Campylobacter* spp., *Listeria* spp., *C. burnetti*, *N. caninum*, other pathogens
Kaveh et al.	2017	Iran	Virus bacteria protozoa	NA	D	128	84	BoHV-1, BVDV, *Leptospira* spp., *N. caninum*
Vidal et al.	2017	Swiss	Bacteria	NA	NA	249	78	*C. burnetii*, Chlamydiaciae family, *Leptospira* spp.
Díaz-Cao et al.	2018	Spain	Virus bacteria protozoa	NA	NA	25	18	BVDV, *Campylobacter* spp., *N. caninum*, other pathogens
Moroni et al.	2018	Chili	Protozoa	NA	NA	296	36	*N. caninum*
Rahal et al.	2018	Algeria	Bacteria	NA	D	73	14	*C. burnetii*, Chlamydiaciae family, *Leptospira* spp.
Rojas et al.	2018	Argentina	Bacteria	1st-2nd- 3rd trimester	NA	251	12	Chlamydiaciae family
Açici et al.	2019	Turkey	Protozoa	2nd-3rd trimester	D	88	43	*N. caninum*
Morrell et al.	2019	Argentina	Virus bacteria protozoa fungi	1st-2nd- 3rd trimester	D, B	150	72	BoHV-1, BVDV, *Leptospira* spp.*, Campylobacter* spp., *Brucella* spp., *Listeria* spp., *N. caninum*, *Tritrichomona foetus*, *Aspergillus* spp., other pathogens.
Serrano-Martínez et al.	2019	Peru	Protozoa	1st-2nd- 3rd trimester	D	68	11	*N. caninum*
Dorsch et al.	2020	Argentina	Protozoa	NA	D, B	303	103	*N. caninum*
Grégoire et al.	2020	Belgium	Bacteria	1st-2nd- 3rd trimester	NA	116	32	*Leptospira* spp.
Macías-Rioseco et al.	2020	Uruguay	Virus bacteria protozoa	1st-2nd- 3rd trimester	D	102	51	BVDV, *C. burnetii*, *Campylobacter* spp., *Leptospira* spp., *N. caninum*, other pathogens
Szeredi et al.	2020	Hungary	Virus bacteria protozoa	NA	NA	387	46	BVDV, *C. burnetii*, *Listeria* spp., *N. caninum*, other pathogens
Wolf-Jäckel et al.	2020	Denmark	Virus bacteria protozoa fungi	1st-2nd- 3rd trimester	D, B	162	68	BVDV, *Listeria* spp., *N. caninum*, other pathogens
Zhang et al.	2020	China	Bacteria	NA	D	66	22	*Brucella* spp., Chlamydiaciae family, other pathogens
Jonker and Michel	2021	South Africa	Bacteria fungi	NA	D, B	193	46	*Brucella* spp., *Campylobacter* spp., *Leptospira* spp., *Listeria* spp., *Aspergillus* spp., other pathogens
Mohabati Mobarez et al.	2021	Iran	Bacteria	NA	NA	46	10	*C. burnetii*
Salehi et al.	2021	Iran	Protozoa	NA	NA	78	16	*N. caninum*
Şevik	2021	Turkey	Virus bacteria	1st-2nd trimester	NA	553	51	BVDV, other pathogens
Van Loo et al.	2021	Belgium	Virus bacteria protozoa fungi	2nd-3rd trimester	D, B	4,006	2,753	BVDV, *C. burnetti*, *Listeria* spp., other pathogens
Villa et al.	2021	Italy	Protozoa	NA	D	198	55	*N. caninum*
de Souza Ribeiro Mioni et al.	2022	Brazil	Bacteria protozoa	NA	NA	76	7	*C. burnetii*
Irehan et al.	2022	Turkey	Protozoa	NA	NA	30	10	*N. caninum*, *Tritrichomonas foetus*, other pathogens
Ntivuguruzwa et al.	2022	Rwanda	Bacteria	NA	NA	19	2	*Brucella* spp.
Saegerman et al.	2022	Belgium	Bacteria	NA	D, B	1,212	103	*Coxiella burnetii*
Thomas et al.	2022	Tanzania	Virus bacteria protozoa	NA	NA	71	26	BHV-1, BVDV, *Brucella* spp., *N. caninum*, other pathogens
Silva da Costa et al.	2022	Brazil	Protozoa	NA	NA	85	20	*N. caninum*

### 3.2. General characteristics of the included studies

The included articles were published from 2000 to 2022, and all articles and all studies that examined one or more infectious agents in bovine abortion cases during this period were included. Overall, the selected works came from 4 continents, although not in the same proportion (Supplementary Figure S1). There were 32 studies from Europe (Belgium:4; Cyprus:1; Denmark:1; Finland:1; Hungary:4; Italy:2; Latvia:1; Netherland:1; Portugal:1; Scotland:2; Serbia:1; Spain:2; Switzerland:4; Turkey:5; United Kingdom:2), 23 studies from North and South America (Argentina:4; Brazil:10; Canada:2; Chile:1; Mexico:2; Peru:1; Uruguay:1; United States:2), 16 studies from Asia (China:3; Iran:11; Israel:1; Republic of Korea:1), and 5 studies from Africa (Algeria:2; Rwanda:1; South Africa:1; Tanzania:1). In 30 of the 76 articles, information about the time of gestation in which reproductive failure occurred was provided. Out of 11,376 aborted foetuses, 4.9% examined cases from 1st–2nd trimester of pregnancy (*n* = 553), 72.4% from the 2nd–3rd trimester (*n* = 8,232) and 22.8% from the 1st–2nd–3rd trimester (*n* = 2,591), without specifying the exact number in each trimester. Moreover, 32 (*n* = 8,576) of the 76 articles included the information about the type of herd production system (dairy or beef herds) from which the analyzed samples proceeded. Of these studies, 24.1% of cases (*n* = 2,064) were collected exclusively from dairy herds and 75.9% (*n* = 6,512) collected from dairy and beef herds without specifying the number of aborted specimens from each farm.

In relation to the analyzed samples, most of them were composed only of foetal tissues (41 studies with 10,700 cases). Only 26 studies (6,905 cases) included foetuses and some of their placentas for the diagnosis of abortion, although the exact number of placentas was not specified. In 5 studies (426 cases), the placenta was the only sample analyzed, in 4 studies (790 cases) only abomasal fluid was studied, and in one study, both placenta and abomasal fluid samples (249 cases) were investigated. A high or moderate grade of autolysis was mentioned in 17 studies in which some foetal tissues or placenta were not available for histopathological examination. In the same way, secondary contaminations that made the diagnosis difficult were mentioned in three studies.

### 3.3. Global estimated prevalence of infectious agents on bovine abortion

Although 76 articles were included in this meta-analysis, only 27 were selected to determine the prevalence of infectious agents related to bovine abortion. These articles included in their analysis the detection of a wide panel of viral, bacterial, protozoan and/or fungal agents to reach the diagnosis of abortion. The other studies (*n* = 49) were excluded from this analysis because their aims were to study the implication of some specific agents in bovine abortion. A total of 10,667 aborted bovine foetuses from 17 countries from America, Europe, Africa, and Asia were examined to detect infections that caused abortion.

**Figure 2:**
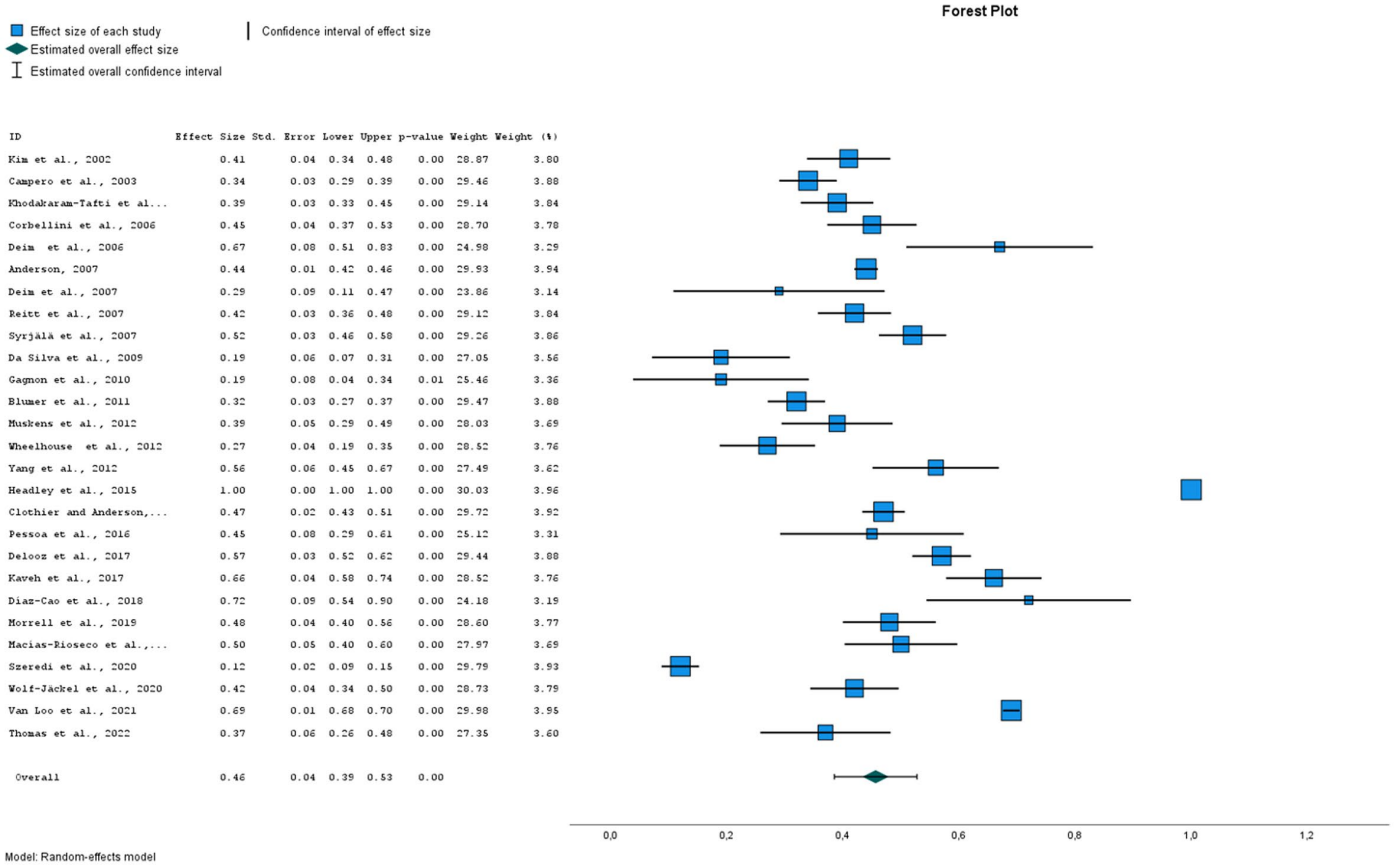
Forest plot of the worldwide prevalence of infectious agents related to bovine abortion. The blue square is the point estimate, and the horizontal line is the 95% confidence interval (CI) for prevalence plotted for each dataset. The left columns show the bibliographic reference for each dataset, the prevalence, the standard error, 95% CI from each dataset, the *p* value and the weight of the study related to the global estimate. The green diamond at the bottom of the forest plot is the worldwide prevalence of infectious agents related to bovine abortion.

**Figure 3:**
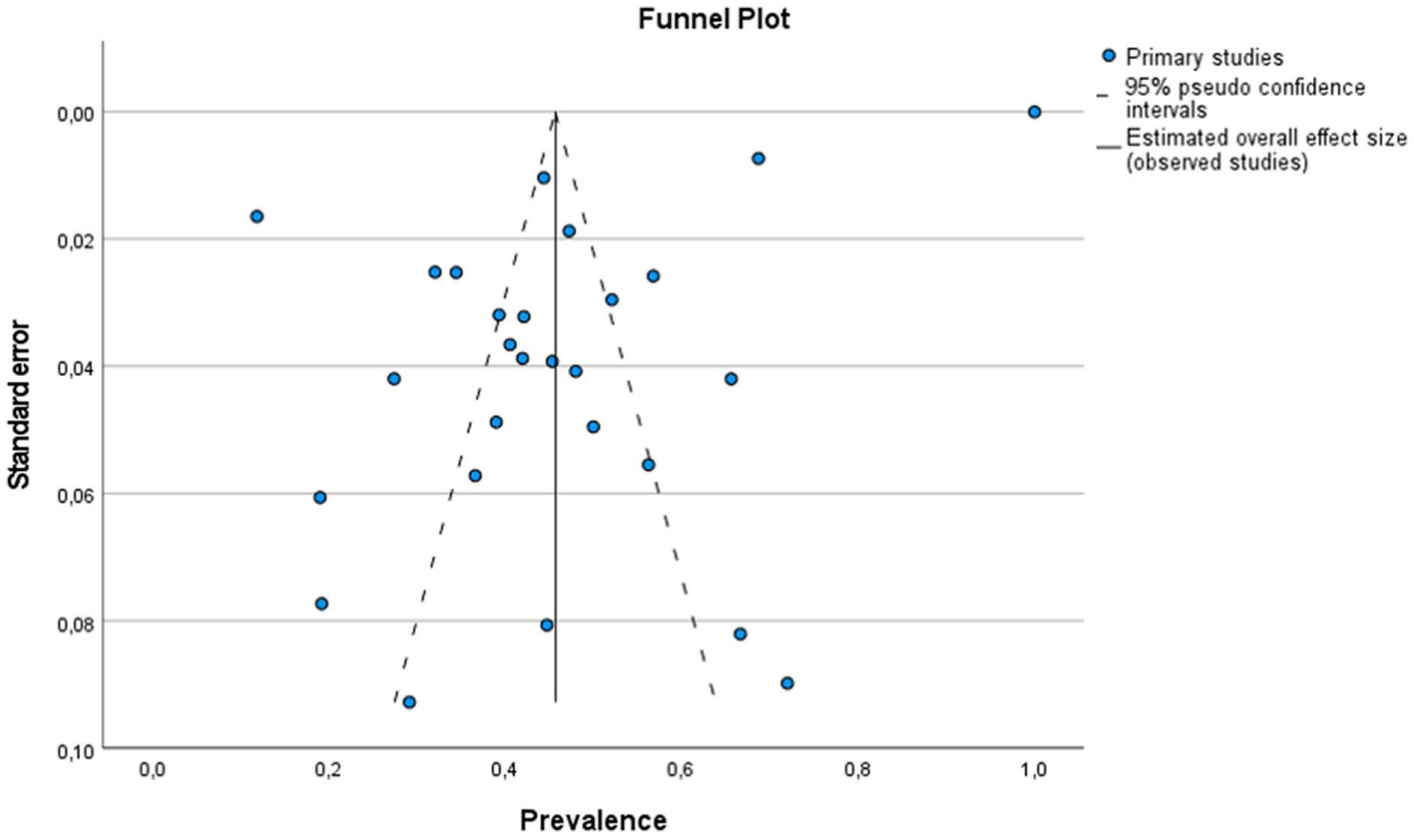
Funnel plot to detect publication bias in studies showing the prevalence of infectious agents on bovine infectious abortion. The circles represent the prevalence in each analyzed study. The continuous lines represent the overall prevalence of all analyzed studies. The prevalence of bovine infectious abortion is plotted on the horizontal axis. The standard error of each study is plotted on the vertical axis.

**Figure 4:**
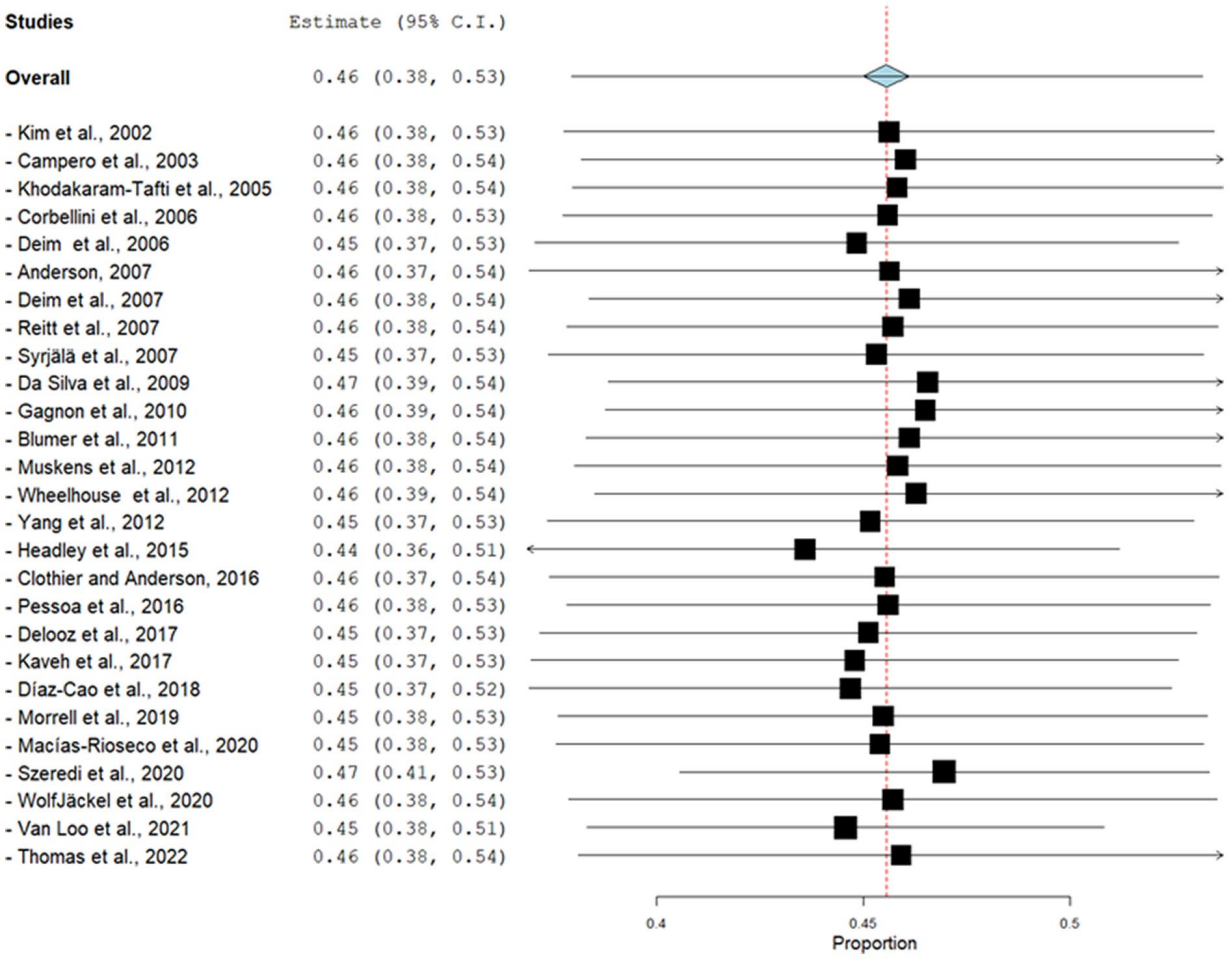
Forest plot of the results of the analysis using the leave-one-out cross-validation (LOOCV) method to determine the sensitivity of the meta-analysis of the estimated prevalence of infectious agents related to bovine abortion. The black square is the point estimate, and the horizontal line is the 95% confidence interval (CI) for prevalence plotted for each dataset. The left columns show the bibliographic reference, the prevalence and the 95% CI from each dataset. The blue diamond at the bottom of the forest plot is a worldwide-pooled prevalence of infectious agents related to bovine abortion.

**Figure 5:**
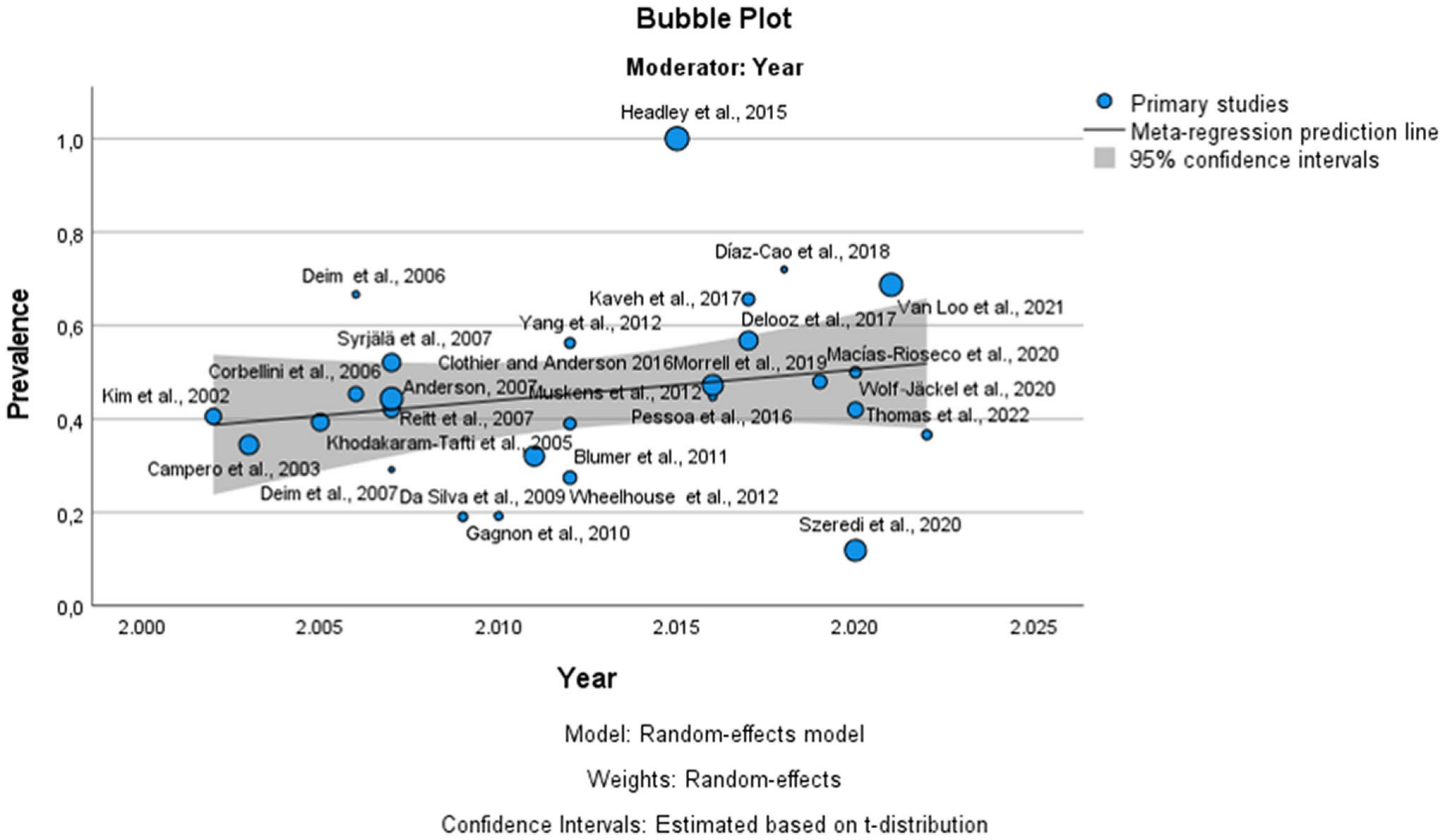
Bubble plot of the results of the meta-regression of published articles against the prevalence of bovine infectious abortion. The circles represent the individual studies. The continuous lines represent the regression lines. The year of publication is plotted on the horizontal axis. The prevalence of bovine infectious abortion is plotted on the vertical axis.

The global estimated prevalence of infectious agents that caused bovine abortion based on the random effects model was 45.7% (95% CI, 38.6–52.8%) (Figure 2). The heterogeneity among studies was high (I^2^ = 99.2%, *p* < 0.001); however, Egger’s test showed that there was no publication bias in the overall prevalence estimated (*p* = 0.239). This fact could be graphically observed in the funnel plot (Figure 3). In addition, the results of the LOOCV method showed that the overall estimated prevalence was not influenced by any specific study (Figure 4). Finally, we assessed the effect of the year of publication on the estimated prevalence of infectious agents related to bovine abortion, and although there was not a significant effect (coefficient = 0.006; *p* = 0.199), an increasing trend was observed over time (Figure 5).

### 3.4. Estimated prevalence of the main infectious agents involved in the bovine abortion

To determine the prevalence of the different transmissible agents in bovine abortion, studies in which some laboratory tests were performed (culture or polymerase chain reaction -PCR- or foetal serology or immunohistochemistry -IHC-) to detect a specific agent (on aborted foetus or placenta) were selected. However, in this analysis were excluded studies in which diagnosis of the abortion cause was made using only maternal serology. On the other hand, the detection of an agent in abortion samples does not confirm its participation in the production of abortion. Therefore, in this study only in the cases in which specific histopathological changes were reported, it was the cause of the abortion considered confirmed. From the 19,070 cases of bovine abortion included in the 76 selected studies, in 7,319 specimens some infectious agents were detected, but only 54.4% (3,977 cases) were confirmed as the cause of foetal death.

According to this meta-analysis, *Neospora caninum* was the most common infectious agent found in cases of bovine abortion, with an estimated prevalence of 22.2% (95% CI: 17.6–26.8) from 9,164 aborted bovine foetuses examined (Supplementary Figure S2a). However, the contribution of this protozoan parasite to foetal death was only confirmed in 16.7% (95% CI: 12.3–21.1) of the analyzed specimens when histopathological changes were found (Table 2).

**Table 2:** Prevalence of each pathogen and subgroup analysis results.

Pathogen			Studies (*n*)	Cases (*n*)	% Estimated prevalence (95% CI)	% *I*^2^ (*p* value)
Virus	BoHV-1	Overall	23	6,006	5.9 (1.5–10.2)	100 (< 0.001)
		Culture	7	1,987	0.9 (−0.2–2.0)	100 (< 0.001)
		*HP	17	5,496	0.9 (0.2–1.5)	100 (< 0.001)
		IHC	11	4,402	0.5 (−0.1–1.1)	100 (< 0.001)
		FS	1	709	3.5 (2.2–4.9)	NA
		PCR	13	1,277	10.4 (1.6–19.3)	100 (< 0.001)
		EAg/DFAT	3	514	2.1 (−0.5–4.7)	84.4 (< 0.001)
		SN	2	205	3.7 (−5–12.5)	81.8 (0.019)
	BVDV	Overall	26	11,348	4.7 (2.7–6.6)	100 (<0.001)
		Culture	4	1,499	1.4 (0.1–2.7)	87.0 (<0.001)
		*HP	13	5,232	1.9 (1.0–2.9)	100 (<0.001)
		IHC	7	4,202	2.3 (0.9–3.8)	88.1 (0.003)
		FS	1	709	2.0 (1.1–2.9)	NA
		PCR	12	2,036	7.3 (3.1–11.5)	100 (<0.001)
		EAg/DFAT	5	5,140	8.0 (2.7–13.3)	96.9 (<0.001)
Bacteria	*Chlamydia* spp.	Overall	19	2,651	10.9 (4.2–17.7)	100 (<0.001)
		Culture	2	246	0.0	NA
		*HP	9	1,623	6.8 (0.0–13.5)	100 (<0.001)
		IHC	7	1,192	1.4 (−0.4–3.2)	100 (<0.001)
		PCR	16	2,204	11.9 (4.8–19.0)	100 (<0.001)
		SS	1	249	4.0 (1.6–6.5)	NA
	*Coxiella burnetii*	Overall	19	7,987	9.5 (3.7–15.3)	100 (<0.001)
		*HP	8	2,008	1.1 (−0.1–2.3)	100 (<0.001)
		IHC	5	1,314	0.0 (−0.0–0.1)	100 (0.187)
		PCR	15	6,515	12.7 (5.6–19.9)	100 (<0.001)
		SS	2	592	2.5 (1.6–6.6)	88.8 (0.003)
	*Leptospira* spp.	Overall	17	5,522	5.2 (1.7–8.6)	100 (<0.001)
		Culture	3	589	2.4 (−1.0–5.8)	91.7 (0.008)
		*HP	9	4,496	5.2 (0.5–9.9)	99.7 (<0.001)
		PCR	9	1,353	6.8 (0.5–13.2)	100 (<0.001)
		DFAT	3	3,155	3.0 (−0.3–6.3)	97.3 (<0.001)
		SS	1	387	0.0	NA
	*Brucella* spp.	Overall	15	4,238	5.1 (1.5–8.6)	100 (<0.001)
		Culture	9	3,452	5.3 (1.0–9.5)	100 (<0.001)
		*HP	7	3,367	3.2 (0.2–6.2)	100 (<0.001)
		IHC	1	354	7.9 (5.1–10.7)	NA
		PCR	9	1,021	7.6 (0.9–14.4)	100 (<0.001)
	*Campylobacter* spp.	Overall	14	5,312	1.3 (0.2–2.3)	100 (<0.001)
		Culture	10	4,611	2.3 (0.5–4.2)	98.6 (<0.001)
		*HP	8	4,218	2.3 (0.3–4.3)	98.4 (<0.001)
		IHC	2	943	0.9 (0.0–1.7)	49.4 (0.160)
		FS	1	709	1.3 (0.4–2.1)	NA
		PCR	5	726	0.0 (−0.1–0.1)	100 (0.096)
	*Listeria monocytogenes*	Overall	13	9,103	2.6 (0.5–5.2)	99.9 (<0.001)
		Culture	12	9,077	2.9 (0.0–5.7)	99.6 (<0.001)
		*HP	9	4,517	1.1 (0.5–1.7)	59.3 (0.009)
		PCR	1	26	0.0	NA
	Opportunistic bacteria	Overall	18	9,824	21.4 (10.4–32.5)	99.8 (<0.001)
		Culture	16	9,768	17.7 (10.7–24.7)	98.8 (< 0.001)
		*HP	12	4,997	12.6 (7.9–17.3)	96.1 (< 0.001)
		PCR	2	56	52.4 (−41–146)	99.9 (< 0.001)
Protozoa	*Neospora caninum*	Overall	45	9,164	22.2 (17.6–26.8)	97.9 (< 0.001)
		*HP	26	6,769	16.7 (12.3–21.1)	97.4 (< 0.001)
		IHC	16	5,676	11.8 (7.4–16.2)	97.4 (< 0.001)
		FS	9	2,015	14.4 (8.6–20.3)	91.4 (< 0.001)
		PCR	36	4,811	21.2 (15.5–26.9)	98.0 (< 0.001)
	*Tritrichomonas foetus*	Overall	5	608	2.3 (−0.1–4.7)	82.5 (<0.001)
		Culture	3	498	0.0 (−0.1–0.1)	100 (0.363)
		*HP	2	252	0.3 (−0.8–1.5)	100 (0.155)
		PCR	3	356	4.5 (1.8–7.1)	25.6 (0.335)
Fungus		Overall	19	10,216	3.4 (1.5–5.3)	99.2 (<0.001)
		Culture	12	6,171	2.9 (1.0–4.7)	97.9 (<0.001)
		*HP	14	5,341	1.4 (0.7–2.0)	89.5 (<0.001)
		PCR	1	650	17.7 (13.7–21.7)	NA
		SS	6	3,708	1.1 (0.5–1.8)	57.2 (0.048)

CI, confidence interval; I2, heterogeneity; NA, not available; HP, histopathology; IHC, immunohistochemistry; FS, foetal serology; EAg, detection of antigen by direct ELISA; SS, special staining; DFAT, direct fluorescence antibody test; *compatible histopathological lesions confirmed the diagnosis.

The presence of sporadically isolated or opportunistic bacteria (*Salmonella* spp. *Escherichia coli*, *Trueperella pyogenes*, *Bacillus licheniformis*, *Pajaroellobacter abortibovis*, *Acinetobacter* spp., *Histophilus somni*, *Actinomyces* spp., *Aeromona* spp., *Bordetella* spp., *Cardiobacterium* spp., *Klebsiella* spp., *Enterobacter* spp., *Pasteurella* spp., *Staphylococcus* spp., *Streptococcus* spp., *Pseudomona* spp., and *Yersinia paratuberculosis*) was observed in 21.4% (95% CI: 10.4–32.5) of the 9,824 analyzed cases from 18 articles (Supplementary Figure S2b). In twelve of these studies (4,997 cases), the authors also searched for histopathological compatible changes, confirming some of these bacteria as causes of foetal death in 12.6% (95% CI: 7.9–17.3) (Table 2).

In third place, the prevalence of the Chlamydiaceae family was 10.9% (95% CI: 4.2–17.7) of 2,651 aborted foetuses (Supplementary Figure S2c). The detection of its infection was mostly carried out by PCR, and only compatible lesions were reported in 6.8% (95% CI: 0.0–13.5) of the cases (Table 2). The specific *Chlamydia* species were only reported in 10 studies (1,817 cases) mentioning the presence of *C. abortus* (90 cases), *C. psittaci* (10 cases), *C. suis* (1 case) and *C. pecorum* (1 case). In addition, five studies mentioned the detection of *Parachlamydia* spp., although its role as an abortigenic agent in these studies could not be confirmed (absence of compatible lesions). On the other hand, the estimated prevalence of *Coxiella burnetii* in bovine abortion was 9.5% (95% CI: 3.7–15.3) in the analyzed cases (*n* = 7,987) (Supplementary Figure S2d), and this agent was detected mostly by PCR (Table 2). Interestingly, this bacterium was confirmed as the cause of abortion (compatible histopathological changes) in only 1.1% (95% CI: −0.1–2.3) of the analyzed cases (*n* = 2,008).

Infection with *Leptospira* spp. was detected in 5.2% (95% CI: 1.7–8.6) of 5,522 aborted foetuses from 17 studies (Supplementary Figure S2e), but in any selected study was mentioned the *Leptospira* species found in the specimen analyzed. This spirochete was detected mostly by PCR and histopathology (HP) and was confirmed as a cause of death in 5.2% (95% CI: 0.5–9.9) of the cases (Table 2). In addition, the search of *Brucella* spp. was made in 4,238 specimens from 15 studies, yielding a prevalence rate of 5.1% (95% CI: 1.5–8.6) (Supplementary Figure S2f), although only it was confirmed as a cause of abortion in 3.2% (95% CI: 0.2–6.2). Only five studies (556 cases) detected *Brucella* spp., including the presence of *B. abortus* (39/556) and *B. melitensis* (22/556). Remarkably, the presence of *Campylobacter* spp. was detected in 1.3% (95% CI: 0.2–2.3) of 5,312 abortion cases (Supplementary Figure S2g) and only in 7 studies (1,805 cases) was the species of *Campylobacter* specified. The species found were *C. fetus* (21/1,805), *C. fetus* subsp. *fetus* (17/1,805) and *C. fetus* subsp. *venerealis* (10/1,805) and *C. jejuni* (4/1,805). The final diagnosis of campylobacteriosis was confirmed in 2.3% (95% CI: 0.3–4.3) of foetal tissues referred for pathological diagnosis (4,218 cases). *Listeria monocytogenes* was detected in 2.6% (95% CI: 0.5–5.2) of the 9,103 analyzed cases (Supplementary Figure S2h). On the other hand, *Tritrichomonas foetus* was detected in 2.3% (95% CI: −0.1–4.7) of 608 cases, although only five included studies reported the presence of this protozoa (Supplementary Figure S2i). Finally, the diagnosis of fungi as causal agents of bovine reproductive failure was investigated in 10,216 cases from 19 studies. The participation of these agents in reproductive bovine failure was 3.4% (95% CI: 1.5–5.3) (Supplementary Figure S2j), with different species of *Aspergillus* being the most commonly diagnosed. In fourteen of these studies (5,341 cases), the authors also searched for histopathological compatible changes, confirming some fungi as causes of foetal death in 1.4% (95% CI: 0.7–2.0) (Table 2).

In the case of viral pathogens, the most commonly involved in bovine reproductive failure were bovine herpes virus type 1 (BoHV-1) and bovine viral diarrhea virus (BVDV) (Table 2). The estimated prevalence of BoHV-1 was 5.9% (95% CI: 1.5–10.2) from 6,006 total cases (Supplementary Figure S2k) and histopathological changes were found in 0.9% (95% CI: 0.2–1.5) of the cases submitted for histopathological examination (*n* = 5,496). In the case of BVDV, the prevalence rate was 4.7% (95% CI: 2.7–6.6) among the 11,348 analyzed cases (Supplementary Figure S2l) and the diagnosis was confirmed in 1.9% (95% CI: 1.0–2.9) of foetal samples analyzed by histopathology (*n* = 5,232). For both viral pathogens, the viral antigen was detected mostly using PCR or direct ELISA or direct fluorescent antibody tests (DFAT). In addition, in four studies, the presence of BoHV-4 DNA was reported in aborted foetuses, although a clear link between this viral infection and abortion was not provided.

For all pathogens, the heterogeneity among studies was high (Table 2). However, the subgroup analysis based on the diagnostic method showed that regardless of the technique used for its analysis, the heterogeneity remained high, discarding this as the cause of such heterogeneity (Supplementary Figure S3). In addition, the results of the LOOCV method showed that the overall estimated prevalence for each infectious agent was not influenced by any specific study (Supplementary Figure S4). Finally, there was no effect of study year on the prevalence of all pathogens (*p* > 0.05) (Supplementary Figure S5).

## 4. Discussion

Reproductive failure causes a major deleterious impact on cattle operations, potentially resulting in large scale economic losses. In this context, infectious abortion plays a key role because the infectious diagnostic rate in bovine abortion can vary between 32 and 58% (88). The aim of the present study was to estimate the general prevalence of bovine infectious abortion and the relative importance of the main infectious agents involved worldwide. After carrying out a systematic review of the available literature, we found a lack of studies that met our inclusion criteria in countries such as Australia that, together with United States, Argentina, China, and Brazil, represent over 50% of the productive cattle stock worldwide (1). Thus, the data compiled in our study are mainly focused on certain areas of the world for which there are high-quality data, although some countries that are important livestock producers are not represented herein. It is well known that the prevalence of an infectious agent related to abortions is influenced by the region, climatic differences, management measures in each farm and health program implemented (5, 25). Therefore, it is essential to increase the number of studies on bovine abortion to have better understand its prevalence and causes, thereby enabling the development of specific control strategies to reduce its impact on the efficiency of bovine production systems. In addition, it is important to consider that the studies included in this meta-analysis are based on samples submitted by convenience to the diagnostic laboratory. Therefore, the lack of random sampling in the selected studies could bias the results of the present meta-analysis. However, due to the nature of this type of studies it is the most common way to obtain samples for analysis (when abortions occur under field conditions, only some foetuses are recovered and sent to the diagnostic laboratory, and only some of them are in good conditions to be analyzed).

In the analysis of the prevalence of bovine infectious abortion, it was only possible to include data from 17 countries belonging to America, Europe, Africa and Asia based on the inclusion criteria of this meta-analysis; thus, as commented before, this work has been limited to the study of the prevalence in the areas analyzed. The results revealed that the global prevalence of infectious agents related to bovine abortion was 45.7%, which is consistent with previously reported results by other authors that mentioned a prevalence between 25 and 50% (7, 88). However, this estimated prevalence was higher than those reported by Kirkbride (89) and Campero et al. (17). Reasons for the difference in prevalence could be due to the geographical origin of samples, differences in the management of production systems in the different studies, differences in the type of analyzed samples, and the use of different diagnostic methods. Possibly, the aforementioned could also be the causes of the heterogeneity observed in this analysis (I^2^ = 99.2, *p* < 0.001) because the publication bias analysis showed that the prevalence of bovine infectious abortion was not affected by the number of samples analyzed in each study (*p* = 0.239). In addition, it has been mentioned that the implementation of new techniques for the diagnosis of infectious agents related to bovine abortion could be responsible for the higher prevalence in the most recent reports (4, 5, 7). In the present study, the meta-regression did not show a significant effect of the study year on the prevalence estimate of infectious agents related to bovine abortion, although an increasing trend was observed over time (Figure 5).

Among the analyzed agents, *N. caninum* was the agent with the highest prevalence (22.2%). Previous studies have mentioned *N. caninum* as one of the main transmissible agents responsible for bovine abortion, probably due to the high efficiency of vertical transmission of this protozoan parasite (90). However, although some infected cows could abort after transplacental transmission, many foetal infections produce only congenitally infected offspring. In the present meta-analysis, *N. caninum* was confirmed as the cause of bovine abortion in 16.7% of 6,769 specimens that were submitted to histopathological studies in which compatible lesions were found in foetal brain or in other target tissues like heart, lung, tongue, or placenta. The authors detected that in some studies, the interpretation of the laboratory results was not correct, establishing, for example, a definitive diagnosis based on the detection of maternal antibodies against *N. caninum* (80). These studies were excluded of our analysis. Only studies in which the parasite was detected in the foetus, or its placenta were included. This fact shows that there are still deficiencies in the interpretation of laboratory results that could contribute to further reducing diagnostic efficiency.

The prevalence of opportunistic bacteria was estimated in 21% of 9,824 analyzed samples, including bacteria in this group that are sporadically involved as causal agents of abortions, such as *Salmonella* spp. *E. coli*, *T. pyogenes*, *B. licheniformis* and *P. abortibovis*, the cause of Epizootic Bovine Abortion. The latter is an important bovine reproductive pathogen in cattle grazing foothill rangelands from United States but has not been reported in other geographical areas (4, 25). Nevertheless, the isolation of these agents does not confirm them as the cause of the abortion because many of them are common inhabitants of the cow reproductive tract or can be an accidental contamination of the sample (25). In the present study, autolysis or secondary contamination of some samples or incorrect sample submission (especially nonsubmission of the placenta) was mentioned in 20 of the 76 analyzed studies. Only 36.21% of 19,070 analyzed cases were sending the foetuses with some of their placenta. The placenta is not usually collected for diagnosis because 1) might be retained in some abortions, 2) placentophagy, 3) presence of predatory animals that feed on the placenta, or 4) inability to recover the placenta in abortion cases occurring in extensive field conditions. However, the placenta is a fundamental organ in the detection of certain agents, such as *Chlamydia* spp., *C. burnetii, B. lichenifomis* or fungi (4, 5, 25, 91). Therefore, the non-inclusion of this tissue in diagnosis could contribute to underestimating the importance of some agents in abortion production.

In this work, infection with bacteria of the Chlamydiaceae family was detected in 10.9% of the analyzed samples, while some studies have described a prevalence higher than 40% (26, 39). On the other hand, the prevalence of *C. burnetii* detected in the present study was 9.5% of 7,987 samples analyzed, although in most cases, only infection with this agent was detected by PCR. In fact, its diagnosis was confirmed only in 1.1% of cases submitted to the pathology laboratory. Agerholm (92) mentioned that confirmation of an association between lesions and the presence of the organism is mandatory to confirm *C. burnetii* as the cause of foetal disease. Additionally, the PCR technique is commonly used for the detection of other agents, such as BoHV-1, *Leptospira* spp., *Chlamydia* spp., or *N. caninum* (7, 25, 93, 94). This diagnostic technique is chosen in certain cases where traditional diagnostic methods are laborious and costly. Nonetheless, a positive PCR result indicates the presence of the analyzed infectious agent, but it does not confirm that this infection was the cause of the abortion.

In addition, six studies mentioned the presence of the related *Chlamydia*-like organisms *Parachlamydia* spp., *Rhabdochlamydia* spp. or *Waddlia* spp. However, they were identified in combination with other more extensively characterized infectious agents (26, 36, 37, 56, 63). The presence of these microorganisms could be associated with contamination of the placental tissues during parturition with environmental *Parachlamydia* spp. (49). A similar situation occurred with the detection of BoHV-4 in four studies that reported the presence of DNA of this virus in the analyzed specimens (22, 27, 57, 61). Previous studies hypothesized that BoHV-4 induces immunosuppression that could enhance the proliferation of opportunistic bacteria, thus inducing reproductive failure (61, 95). However, the role of BoHV-4 as an aetiological agent of bovine abortion needs further study.

On the other hand, the presence of BoHV-1 and BVDV was analyzed in the included studies, and similar prevalence rates (between 5 and 6%) and final diagnosis rates (between 1 and 2%) were observed for both viral pathogens, consistent with previous reports (4, 7, 17, 72). Although abortion induced by BoHV-1 is only a sequel to respiratory infection and viremia, some authors mentioned that BoHV-1 was responsible for abortion in dairy herds in 36.3% of the cases analyzed (50). However, our results agree with other studies that mentioned a low prevalence of this virus in bovine abortions (45, 96), probably because in some countries eradication efforts have been successful.

Otherwise, foetal infection with BVDV is a common finding, however its importance as cause of abortion must be carefully considered. The outcomes of its infection during gestation depend on the moment of the infection and the biotype involved (5, 25). An important epidemiological aspect of foetal BVDV infection is that infections in foetuses prior to 4 months of gestation with a non-cytopathic BVDV can result in persistently infected (PI) live offspring that although it does not present antibodies, it is continuously eliminating the virus being a major source of infection for other cattle. In addition, foetal infections after 4 months of gestation often result in the development of a foetal immune response, with development of specific foetal antibodies, and these infections cannot result in abortions although the virus antigen is detected. Therefore, the importance of this virus as cause of abortion may be overestimated as the virus may infect the foetus without causing its death and there are not specific foetal lesions attributed to infection making accurate diagnosis difficult. Therefore, to confirm BVDV as the cause of abortion, the diagnosis of viral infection needs to be combined with the herd history. The prevalence of BVDV-positive specimens has been decreasing in the most recent studies as eradication and vaccination programs progress in different countries (88). In the present meta-analysis, the heterogeneity among the studies diagnosing BVDV was high, and one of the reasons for this was the different national BVDV control context because the subgroup analysis showed that said heterogeneity could not be associated with the techniques used to make the diagnosis.

In the present study, most of the analyzed foetuses recovered during the 2^nd^ and 3^rd^ (72.36%) trimesters of gestation. This situation has been previously mentioned by others (5) because during the first trimester of pregnancy, most foetal deaths go unnoticed, and abortion in extensive grazing conditions is more difficult to return to the laboratory. This fact could influence the prevalence obtained for certain agents that could be underestimated. This is the case for *T. foetus*, *Campylobacter* spp. or BVDV (6). In the present meta-analysis study, infection with *T. foetus* was estimated in 2.3% of the 608 studied cases, while for *C. fetus,* it was estimated in 1.3% of the 5,312 aborted cases, in agreement with the prevalence obtained in previous studies (7, 50). It is important to consider that the prevalence of these agents can also be influenced by the biotype animal and management conditions of each farm, which are possible causes of heterogeneity found in the analysis of these pathogens.

In conclusion, the results of this meta-analysis showed that the prevalence of infectious agents related to bovine abortion was approximately 50% of the analyzed cases. According to this study, *N. caninum* was the most commonly detected agent in bovine abortion, followed by opportunistic bacteria, the Chlamydiaceae family and *C. burnetii*, although the last two bacteria in most cases only infection was determined by PCR. Although the application of new techniques has improved the identification of infectious agents in abortions, the diagnosis of transmissible bovine abortion remains often incomplete and in some cases is only based on the detection of the agent and/or serological analyzes. An enhanced diagnostic is the key to establishing specific control strategies to reduce the impact of abortifacient agents on the efficiency of bovine productive systems.

## Data availability statement

The original contributions presented in the study are included in the article/Supplementary material, further inquiries can be directed to the corresponding authors.

## Author contributions

PH and LOM conceived the study. YH, PH, and LOM coordinated and organized the systematic review and reviewed and edited the manuscript. YH and SGO surveyed the literature and extracted and reviewed the data. YH, SGO, PH, and LOM collected and selected the articles. YH drafted the manuscript and designed tables and figures. SC and YH performed the statistical analysis. All authors contributed to the article and approved the submitted version.

## Footnotes

^1^ https://pubmed.ncbi.nlm.nih.gov/

^2^ https://www.scopus.com/
