# Supplementary material for: Bovine infectious abortion: a systematic review and meta-analysis
Source: Front Vet Sci. 2023 Sep 29;10:1249410. doi: 10.3389/fvets.2023.1249410 (PMC10570746; doi:10.3389/fvets.2023.1249410)

Supplementary Material

Bovine transmissible abortion: a systematic review and meta-analysis

Yanina Paola Hecker, Sara González Ortega, Santiago Cano, Luis Miguel Ortega Mora*, Pilar Horcajo*

*** Correspondence:** Corresponding Author: Luis Miguel Ortega Mora and Pilar Horcajo

luis.ortega@ucm.es / phorcajo@ucm.es

# Supplementary Figures and Tables

## Supplementary Table 1: Quality assessment of included studies based on the Newcastle-Ottawa Scale.

| Reference | **Year** | **Country** | **Selection** (0-3 points) | **Comparability** (0-2 points) | **Outcome** (0-3 points) | **Total** (scores) |
| --- | --- | --- | --- | --- | --- | --- |
| Schock *et al.* | 2000 | Scotland | 2 | 1 | 3 | 6 |
| Corbellini *et al.* | 2002 | Brazil | 2 | 1 | 3 | 6 |
| Kim *et al.* | 2002 | Korea | 3 | 2 | 3 | 8 |
| Campero *et al.* | 2003 | Argentina | 3 | 2 | 3 | 8 |
| Pereira-Bueno *et al.* | 2003 | Spain | 3 | 1 | 3 | 7 |
| Khodakaram-Tafti *et al.* | 2005 | Canada | 3 | 2 | 2 | 7 |
| Takiuchi *et al.* | 2005 | Brazil | 2 | 1 | 2 | 5 |
| Corbellini *et al.* | 2006 | Brazil | 3 | 2 | 3 | 8 |
| Deim *et al.* | 2006 | Hungary | 2 | 1 | 2 | 5 |
| Medina *et al.* | 2006 | Mexico | 2 | 1 | 2 | 5 |
| Parisi A. *et al.* | 2006 | Italy | 3 | 1 | 3 | 7 |
| Anderson | 2007 | USA | 3 | 2 | 2 | 7 |
| Borel *et al.* | 2007 | Swiss | 3 | 1 | 2 | 6 |
| Deim *et al.* | 2007 | Hungary | 2 | 1 | 2 | 5 |
| Pescador *et al.* | 2007 | Brazil | 3 | 1 | 3 | 7 |
| Razmi *et al.* | 2007 | Iran | 3 | 1 | 2 | 6 |
| Reitt *et al.* | 2007 | Swiss | 3 | 2 | 2 | 7 |
| Sadrebazzaz *et al.* | 2007 | Iran | 2 | 1 | 2 | 5 |
| Syrjälä *et al.* | 2007 | Finland | 3 | 2 | 2 | 7 |
| Da Silva *et al.* | 2009 | Brazil | 2 | 1 | 3 | 6 |
| Yao *et al.* | 2009 | China | 2 | 0 | 3 | 5 |
| Gagnon *et al.* | 2010 | Canada | 2 | 2 | 2 | 6 |
| Wheelhouse *et al.* | 2010 | United Kingdom | 2 | 0 | 2 | 4 |
| Blumer *et al.* | 2011 | Swiss | 3 | 2 | 2 | 7 |
| Cantas *et al.* | 2011 | Cyprus | 2 | 0 | 3 | 5 |
| Clemente *et al.* | 2011 | Portugal | 2 | 0 | 2 | 4 |
| dos Santos *et al.* | 2011 | Brazil | 2 | 1 | 2 | 5 |
| Ghalmi *et al.* | 2011 | Algeria | 2 | 1 | 3 | 6 |
| Mazuz *et al.* | 2011 | Israel | 2 | 1 | 2 | 5 |
| Safarpoor Dehkordi *et al.* | 2011 | Iran | 3 | 1 | 3 | 7 |
| Albayrak *et al.* | 2012 | Turkey | 2 | 0 | 2 | 4 |
| Crook *et al.* | 2012 | United Kingdom | 3 | 1 | 2 | 6 |
| Momtaz and Moshkelani | 2012 | Iran | 3 | 0 | 2 | 5 |
| Muskens *et al.* | 2012 | Netherlands | 3 | 2 | 2 | 7 |
| Safarpoor Dehkordi *et al.* | 2012 | Iran | 3 | 0 | 3 | 6 |
| Wheelhouse *et al.* | 2012 | Scotland | 3 | 2 | 2 | 7 |
| Yang *et al.* | 2012 | China | 2 | 0 | 2 | 4 |
| Guven *et al.* | 2013 | Turkey | 3 | 1 | 3 | 7 |
| Safarpoor Dehkordi *et al.* | 2013 | Iran | 3 | 0 | 3 | 6 |
| Šteingolde *et al.* | 2013 | Latvia | 3 | 0 | 3 | 6 |
| Kamali *et al.* | 2014 | Iran | 3 | 1 | 3 | 7 |
| Headley *et al.* | 2015 | Brazil | 2 | 2 | 2 | 6 |
| Kreizinger *et al.* | 2015 | Hungary | 2 | 2 | 2 | 6 |
| Clothier and Anderson | 2016 | USA | 3 | 3 | 2 | 8 |
| Cvetojević *et al.* | 2016 | Serbia | 2 | 0 | 3 | 5 |
| Medina-Esparza *et al.* | 2016 | Mexico | 2 | 0 | 3 | 5 |
| Pessoa *et al.* | 2016 | Brazil | 2 | 2 | 3 | 7 |
| Barati *et al.* | 2017 | Iran | 2 | 0 | 2 | 4 |
| Delooz *et al.* | 2017 | Belgium | 3 | 2 | 3 | 8 |
| Kaveh *et al.* | 2017 | Iran | 3 | 0 | 2 | 5 |
| Vidal *et al.* | 2017 | Swiss | 3 | 2 | 3 | 8 |
| Díaz-Cao *et al.* | 2018 | Spain | 2 | 2 | 2 | 6 |
| Moroni *et al.* | 2018 | Chile | 3 | 1 | 2 | 6 |
| Rahal *et al.* | 2018 | Algeria | 2 | 0 | 2 | 4 |
| Rojas *et al.* | 2018 | Argentina | 3 | 1 | 2 | 6 |
| Açici *et al.* | 2019 | Turkey | 2 | 0 | 3 | 5 |
| Morrell *et al.* | 2019 | Argentina | 3 | 2 | 3 | 8 |
| Serrano-Martínez *et al.* | 2019 | Peru | 2 | 1 | 3 | 6 |
| Dorsch *et al.* | 2020 | Argentina | 3 | 1 | 3 | 7 |
| Grégoire *et al.* | 2020 | Belgium | 3 | 1 | 3 | 7 |
| Macías-Rioseco *et al.* | 2020 | Uruguay | 3 | 2 | 3 | 8 |
| Salehi *et al.* | 2020 | Iran | 2 | 0 | 2 | 4 |
| Şevik | 2020 | Turkey | 3 | 1 | 2 | 6 |
| Szeredi *et al.* | 2020 | Hungary | 3 | 2 | 2 | 7 |
| Wolf‑Jäckel *et al.* | 2020 | Denmark | 3 | 2 | 2 | 7 |
| Zhang *et al.* | 2020 | China | 3 | 2 | 3 | 8 |
| Jonker and Michel | 2021 | South Africa | 3 | 2 | 3 | 8 |
| Mohabati Mobarez *et al.* | 2021 | Iran | 2 | 0 | 2 | 4 |
| Ntivuguruzwa *et al.* | 2021 | Rwanda | 2 | 1 | 3 | 6 |
| Van Loo *et al.* | 2021 | Belgium | 3 | 2 | 3 | 8 |
| Villa *et al.* | 2021 | Italy | 3 | 0 | 3 | 6 |
| Mioni *et al.* | 2022 | Brazil | 2 | 2 | 2 | 6 |
| Irehan *et al.* | 2022 | Turkey | 2 | 1 | 3 | 6 |
| Saegerman *et al.* | 2022 | Belgium | 3 | 0 | 3 | 6 |
| Thomas *et al.* | 2022 | Tanzania | 2 | 1 | 3 | 6 |
| da Costa | 2022 | Brazil | 2 | 0 | 2 | 4 |

**Newcastle - Ottawa Quality assessment scale** (adapted from Modesti *et al*. 2016) Selection (maximum 3)

1) Representativeness of the sample:

a. Truly representative of the average in the target population. Definition of infected specimens is same among articles (1)

b. Selected group of users. (0)

c. No description of the sampling strategy. (0)

2) Sample size:

a. Justified and satisfactory from herds with reproductive problems. (1)

b. Not justified. (0)

3) Method used to assess the pathogen presence (1):

a. Validated measurement tool. (1)

b. Non-validated measurement tool, but the tool is available or described. (1)

c. No description of the measurement tool. (0)

Comparability (maximum 2)

1) Study includes detection of infected and non-infected specimens

a. The studied population includes more of one diagnostic techniques (1)

b. The study does not include only detection of infection but also final diagnosis (1)

Outcome (maximum 3 stars)

1) Assessment of the outcome

a. Independent blind assessment. (2)

b. Record linkage. (2)

c. Self report (1)

d. No description. (0)

2) Statistical test:

a. The statistical test used to analyse the data is clearly described and appropriate, and the measurement of the association is presented and the probability level (p value). (1)

b. The statistical test is not appropriate, not described or incomplete. (0)

## Supplementary Figures

**Supplementary Figure 1.** Map including the countries represented by the 76 papers analysed in this study.


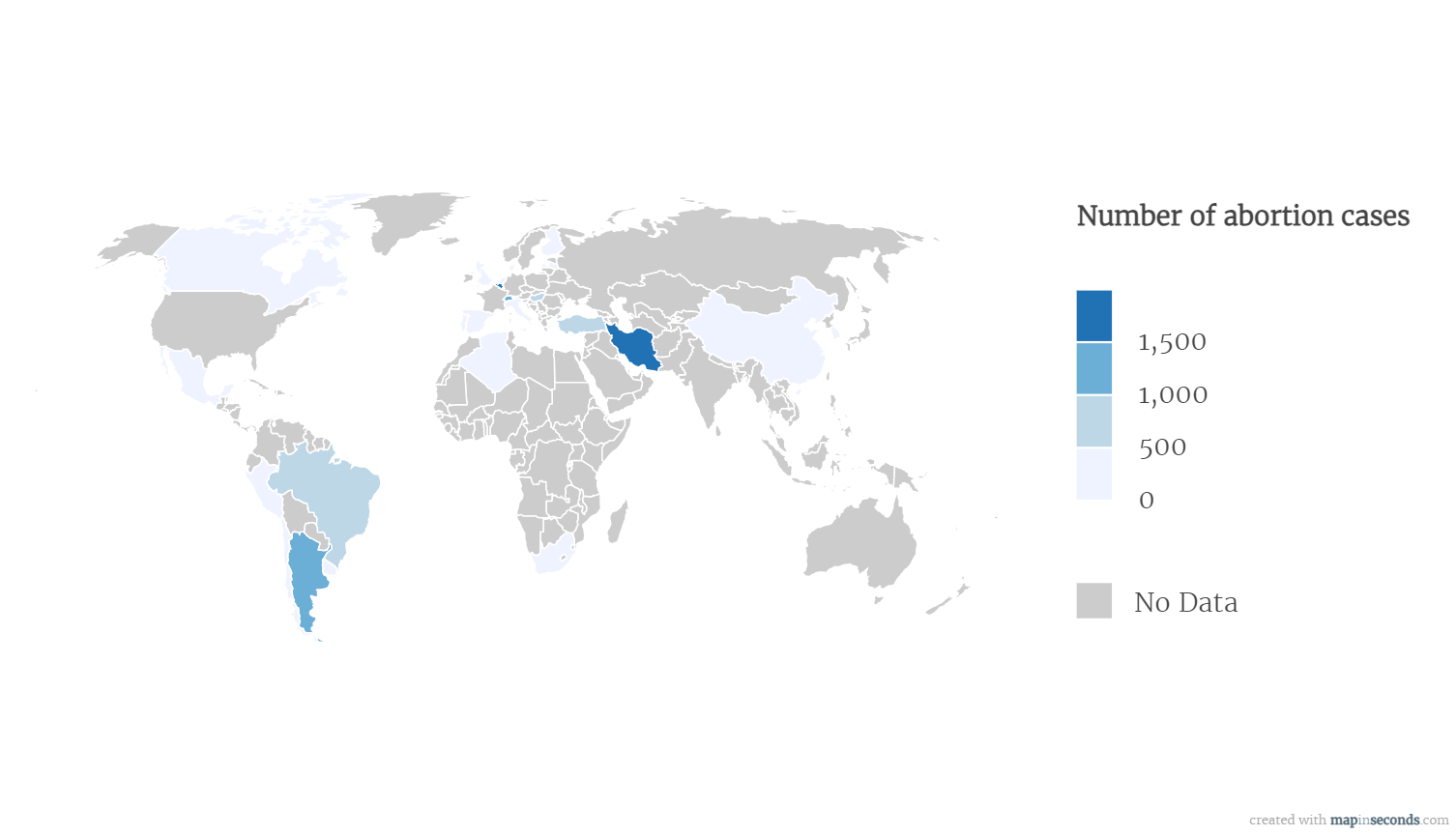


**Supplementary Figure 2.** Forest plot of the worldwide prevalence for each agent related to bovine abortion. The blue square is the point estimate, and the horizontal line is the 95% confidence interval (CI) for prevalence plotted for each dataset. The left columns show the bibliographic reference for each dataset, the prevalence, the standard error, 95% CI from each dataset, the *p* value and the weight of the study related to the global estimate. The green diamond at the bottom of the forest plot is a worldwide pooled prevalence of infectious agents related to bovine abortion. (a) *Neospora caninum*; (b) Opportunistic bacteria; (c) Chlamydiaceae family; (d) *Coxiella burnetii*; (e) *Leptospira* spp.; (f) *Brucella* spp.; (g) *Campylobacter* spp.; (h) *Listeria* spp. (i) *Tritrichomonas foetus*; (j) Fungus; (k) Bovine Herpes Virus type 1; (l)Bovine Viral Diarrhoea.

(a)

(b)
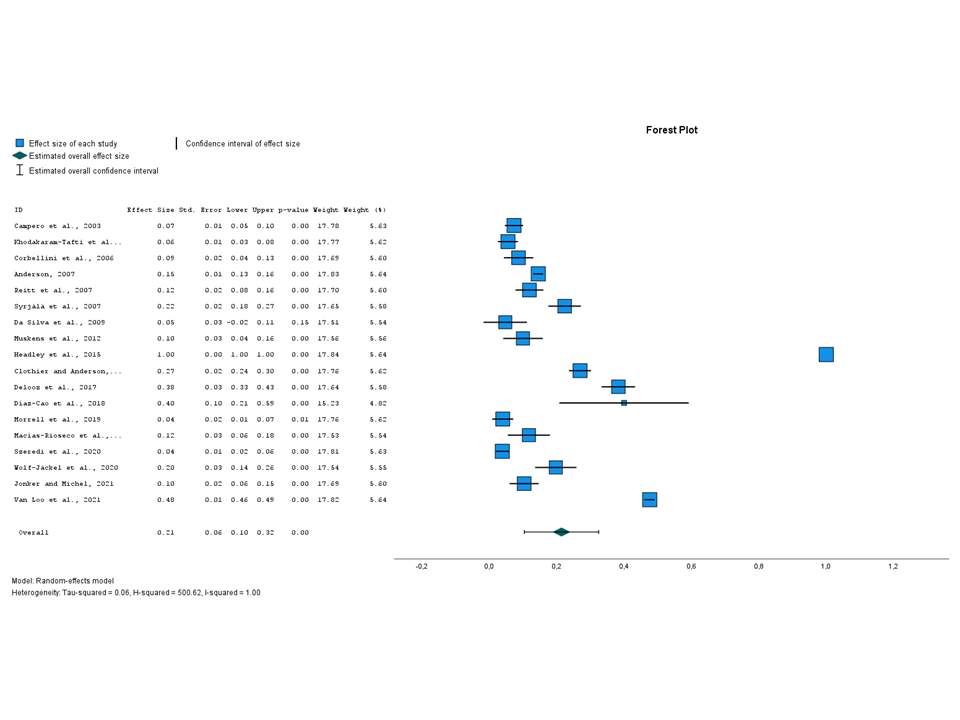


(c)


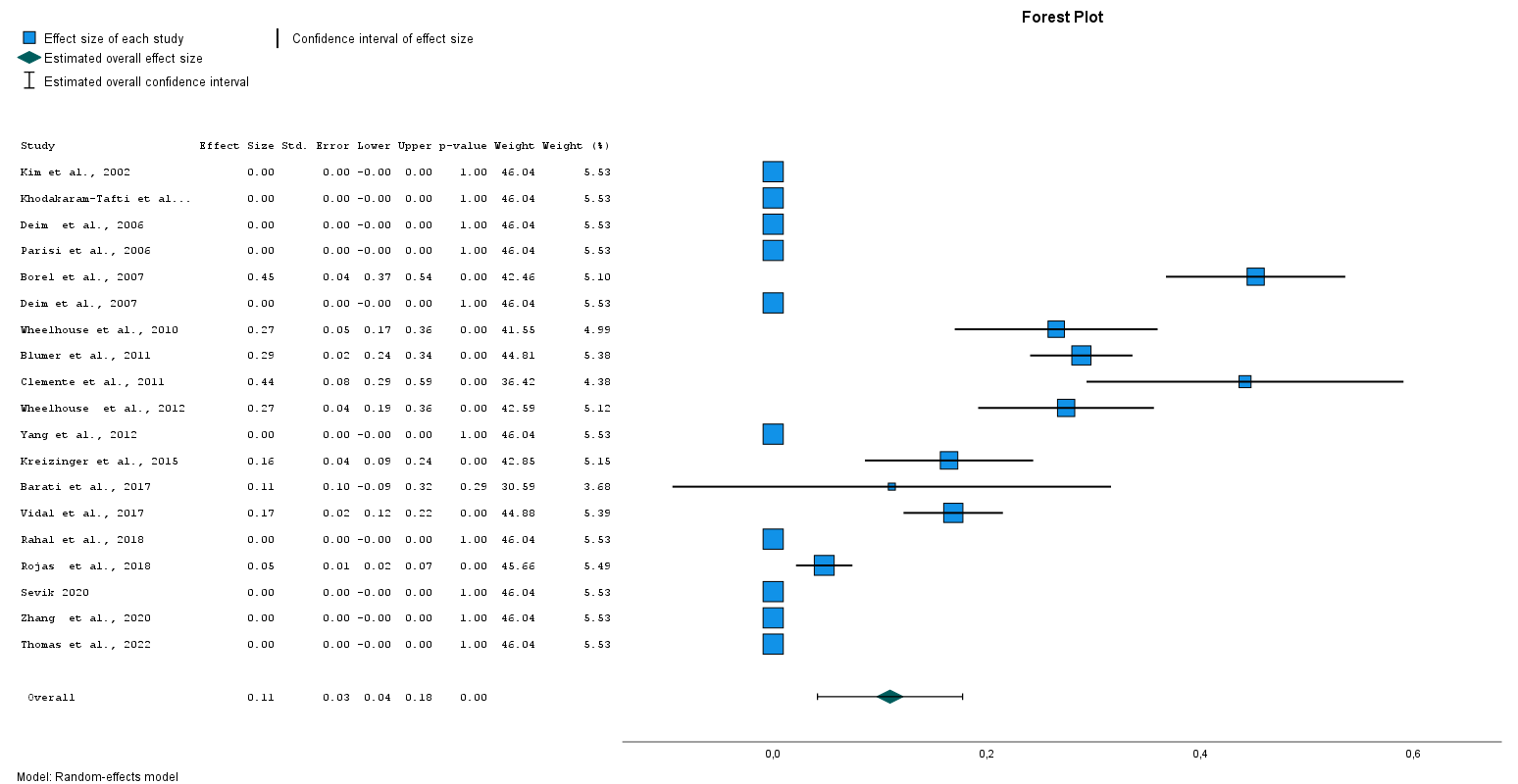


(d)


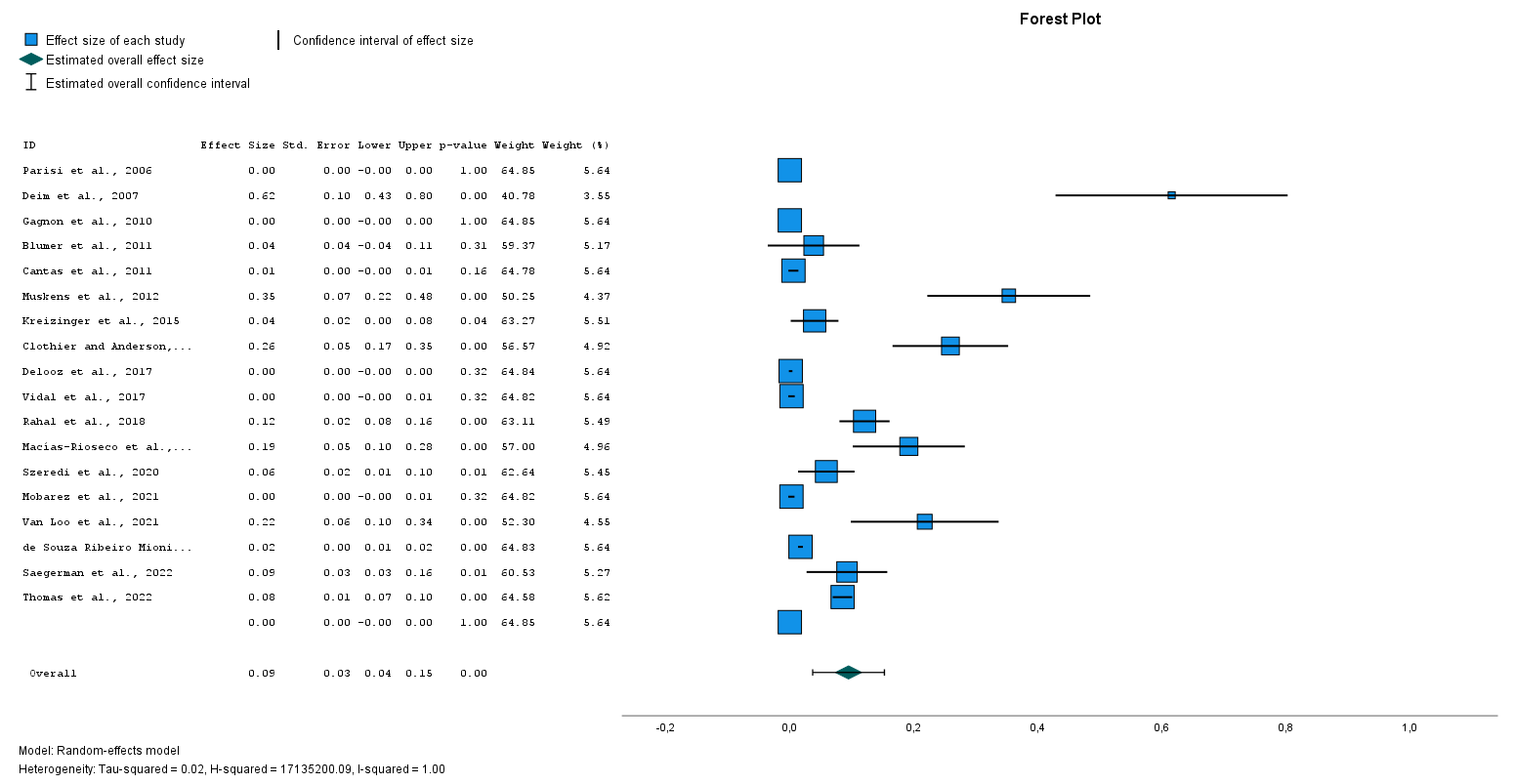


(e)


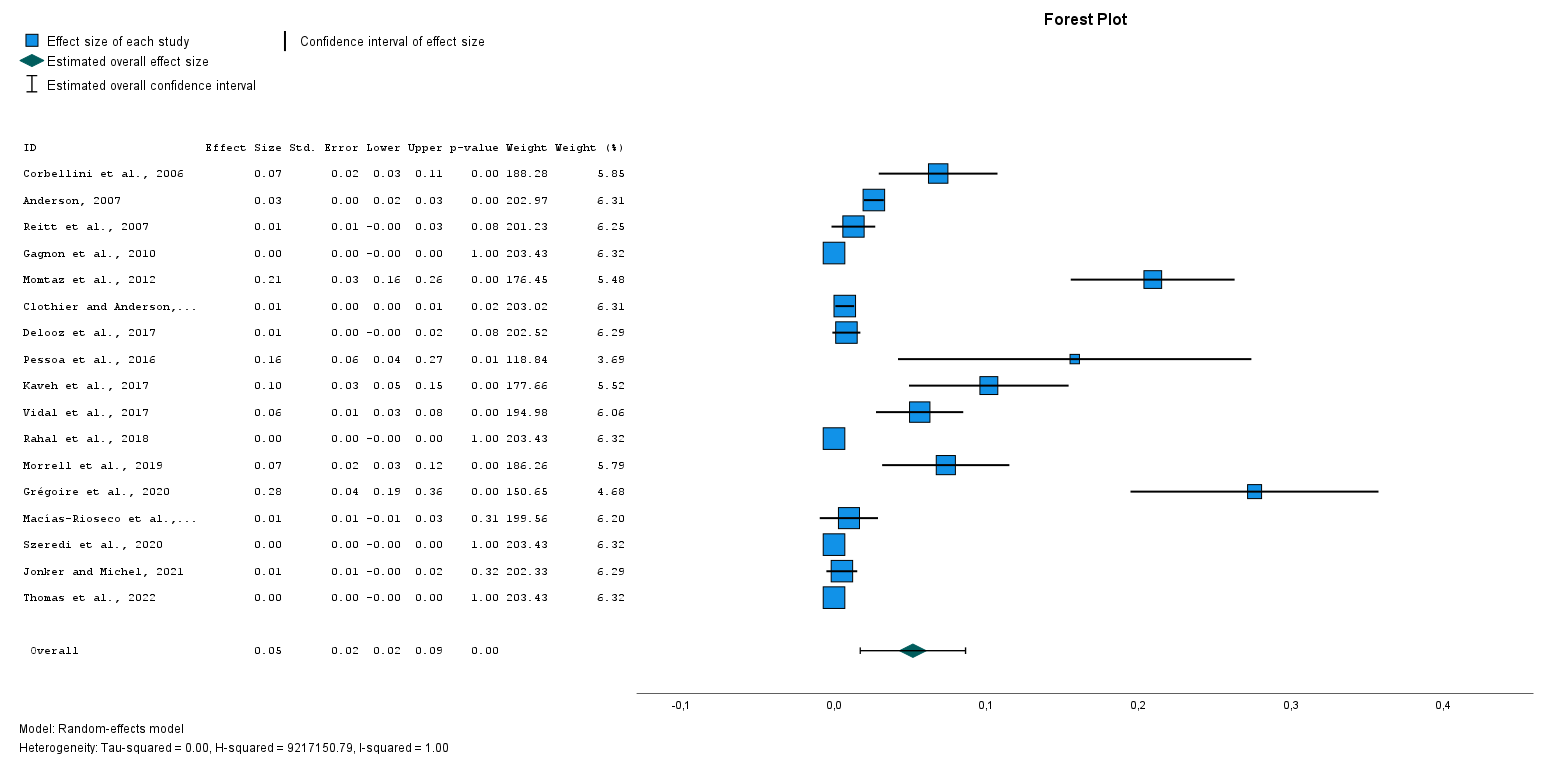


(f)


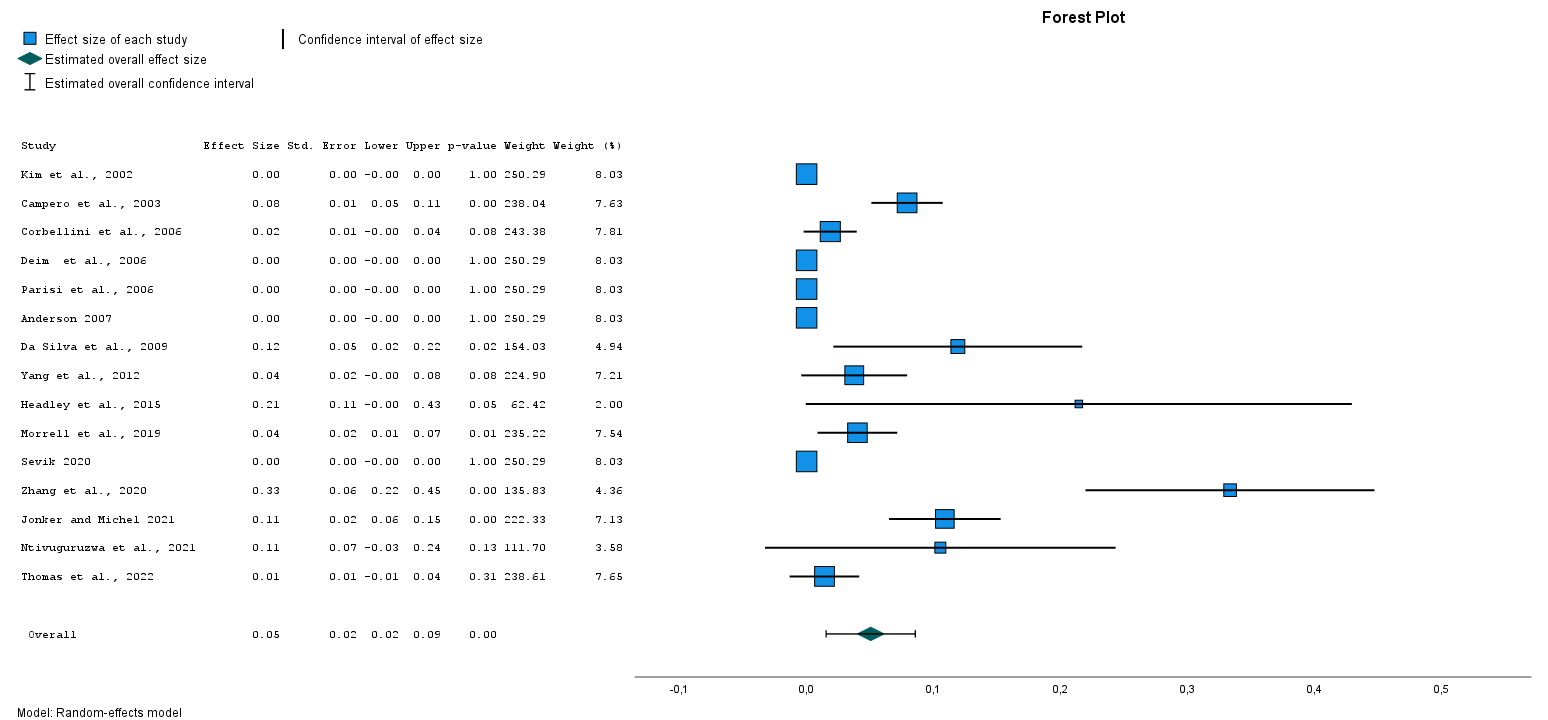


(g)

**
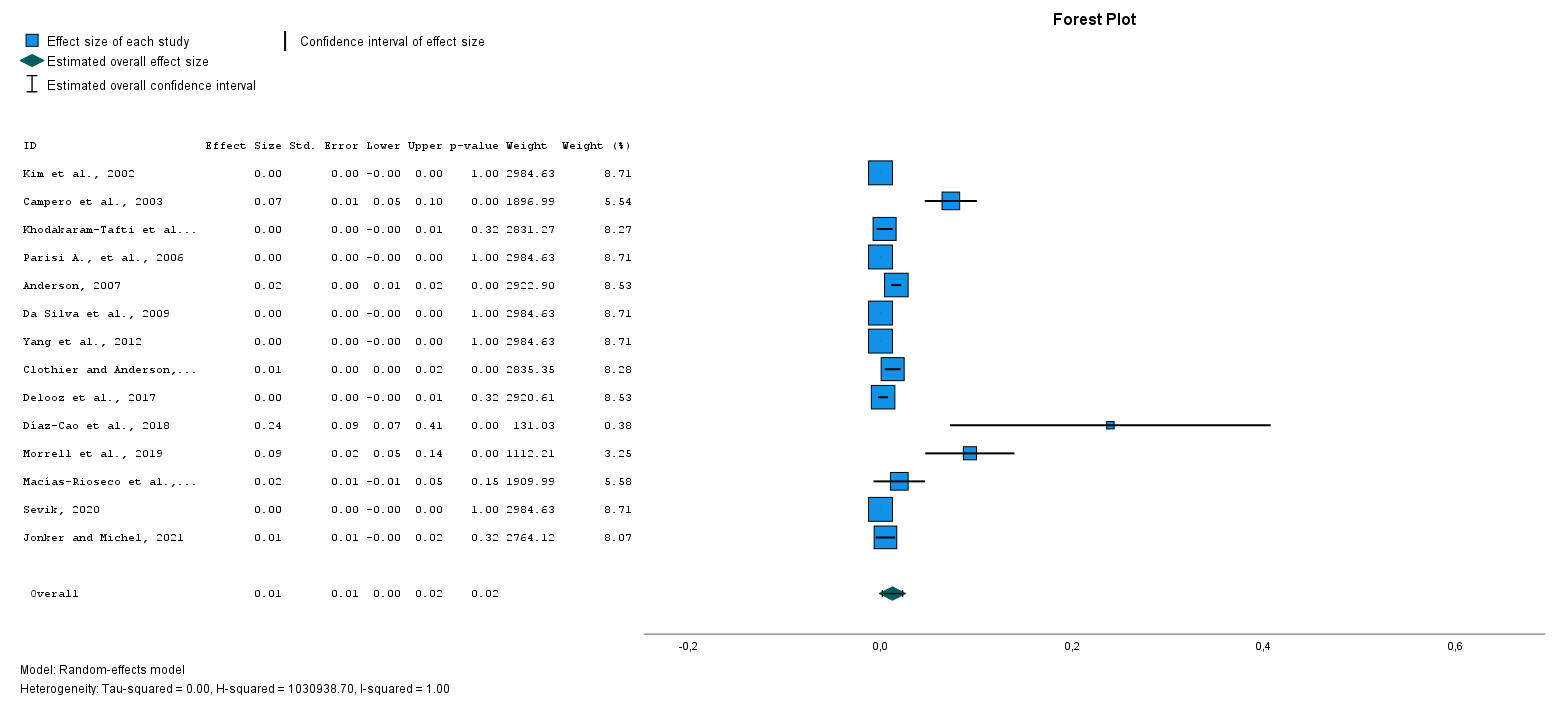
**

(h)


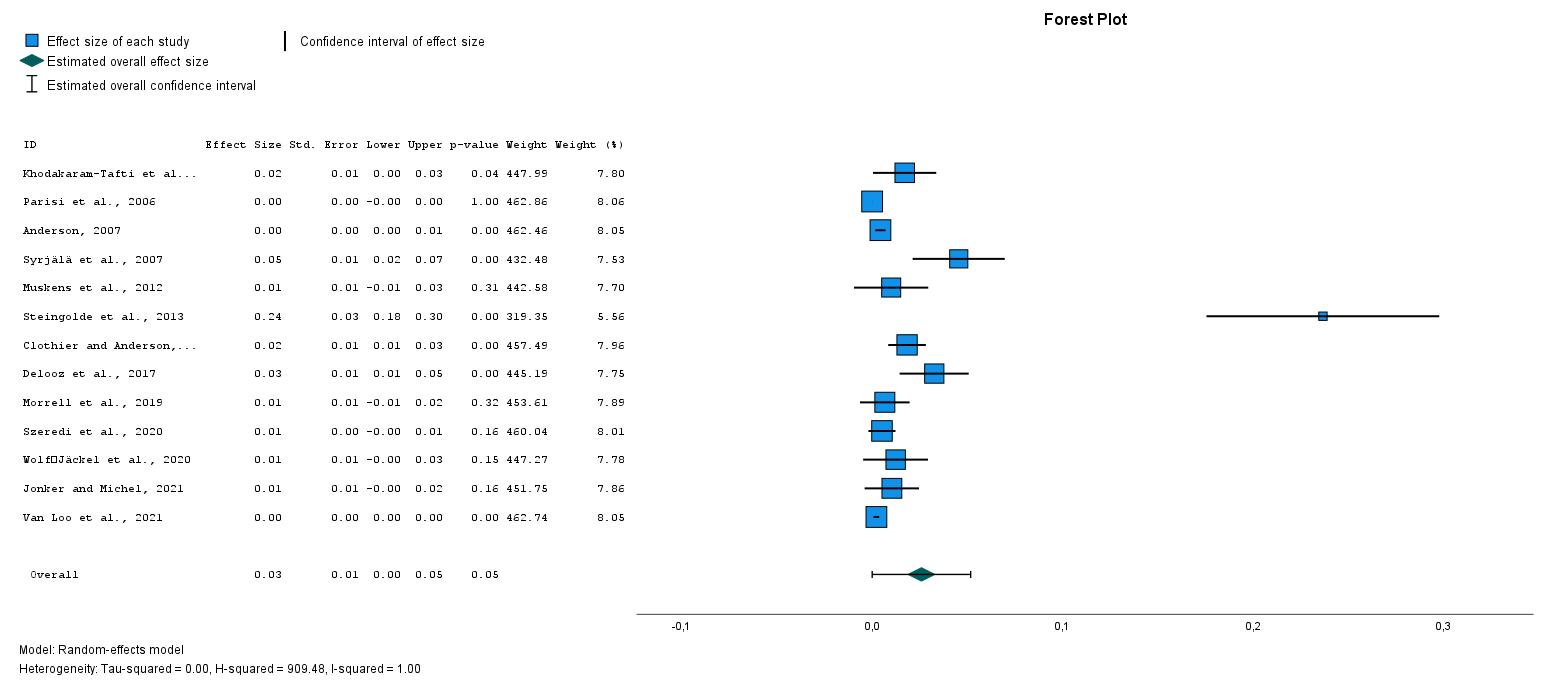


(i)


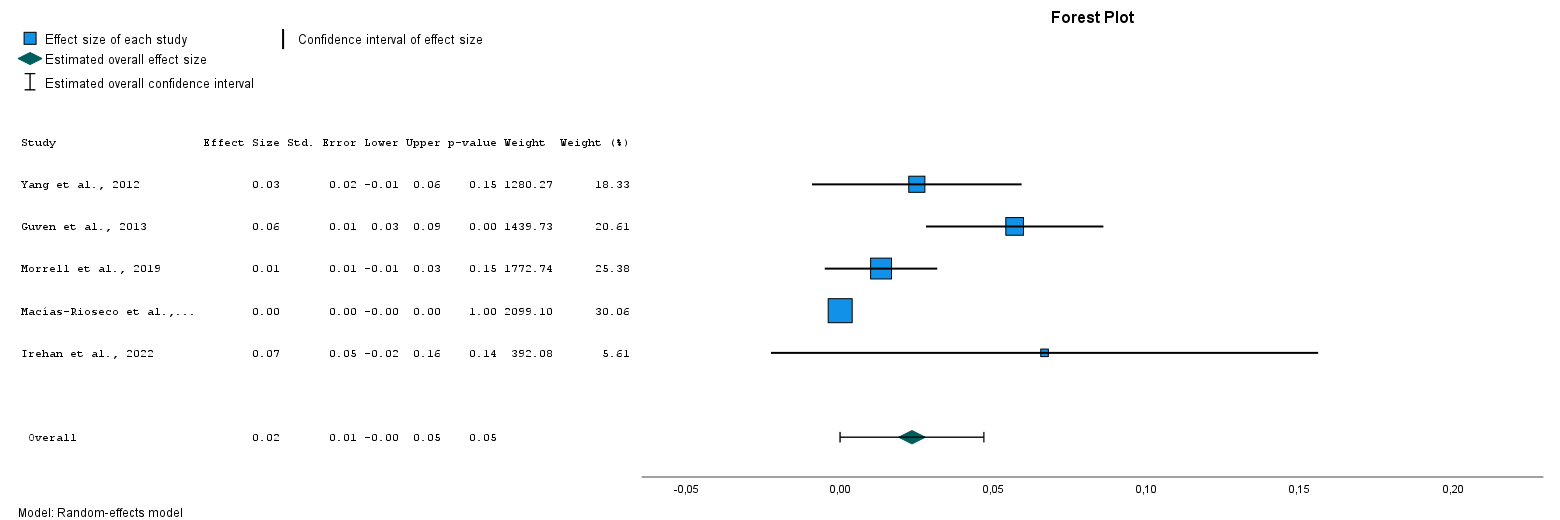


(j)

**
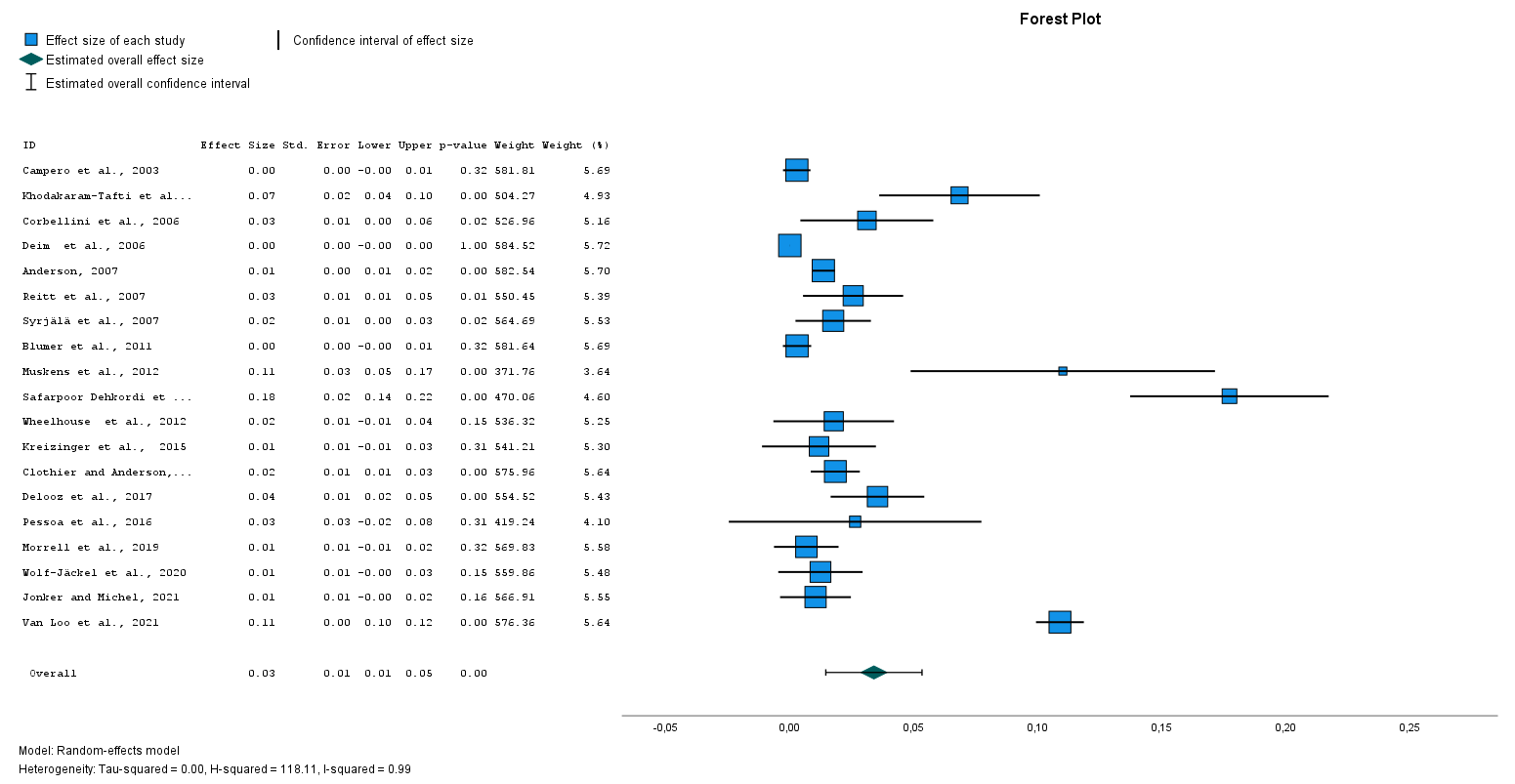
**

(k)


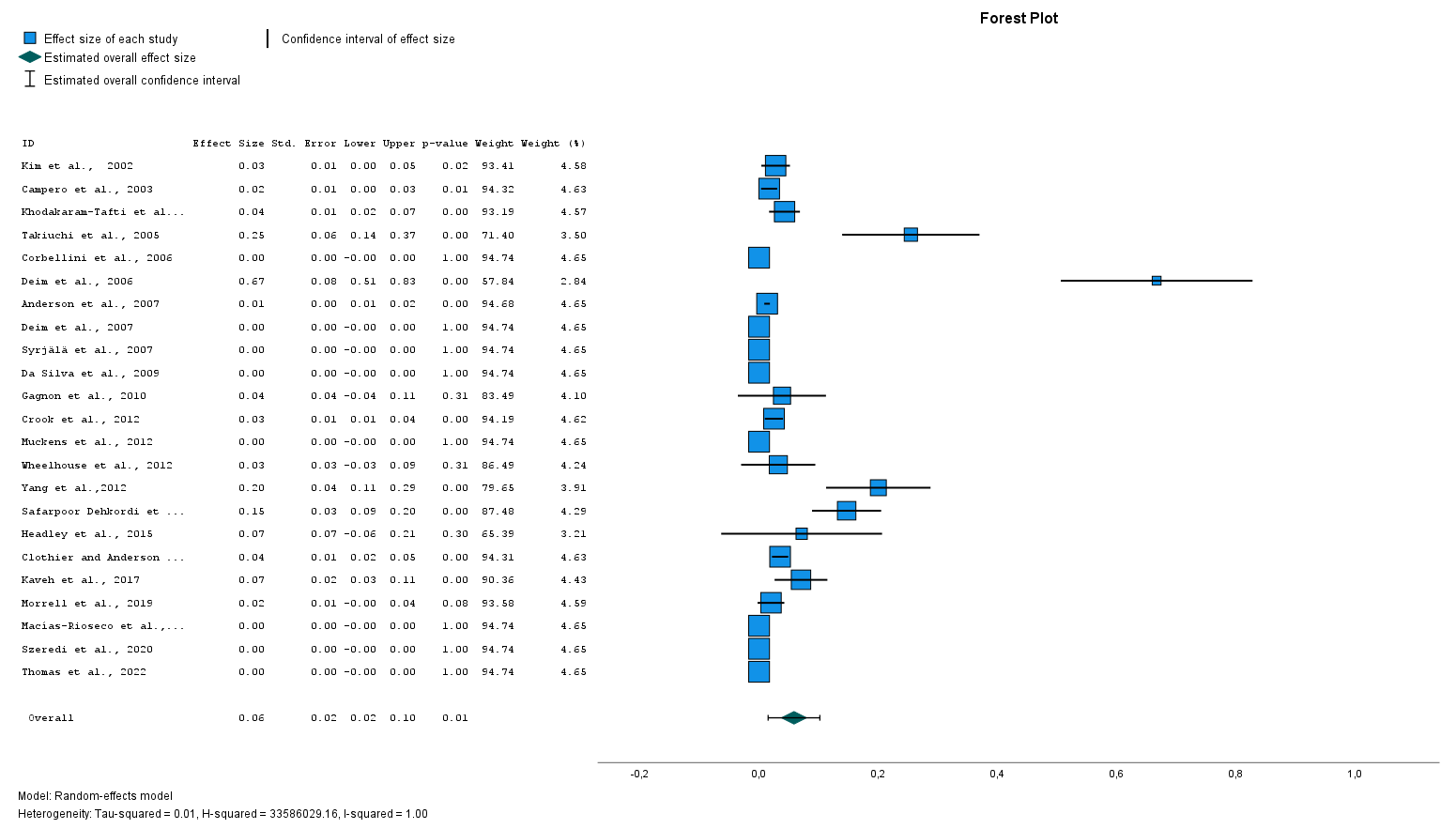


(l)


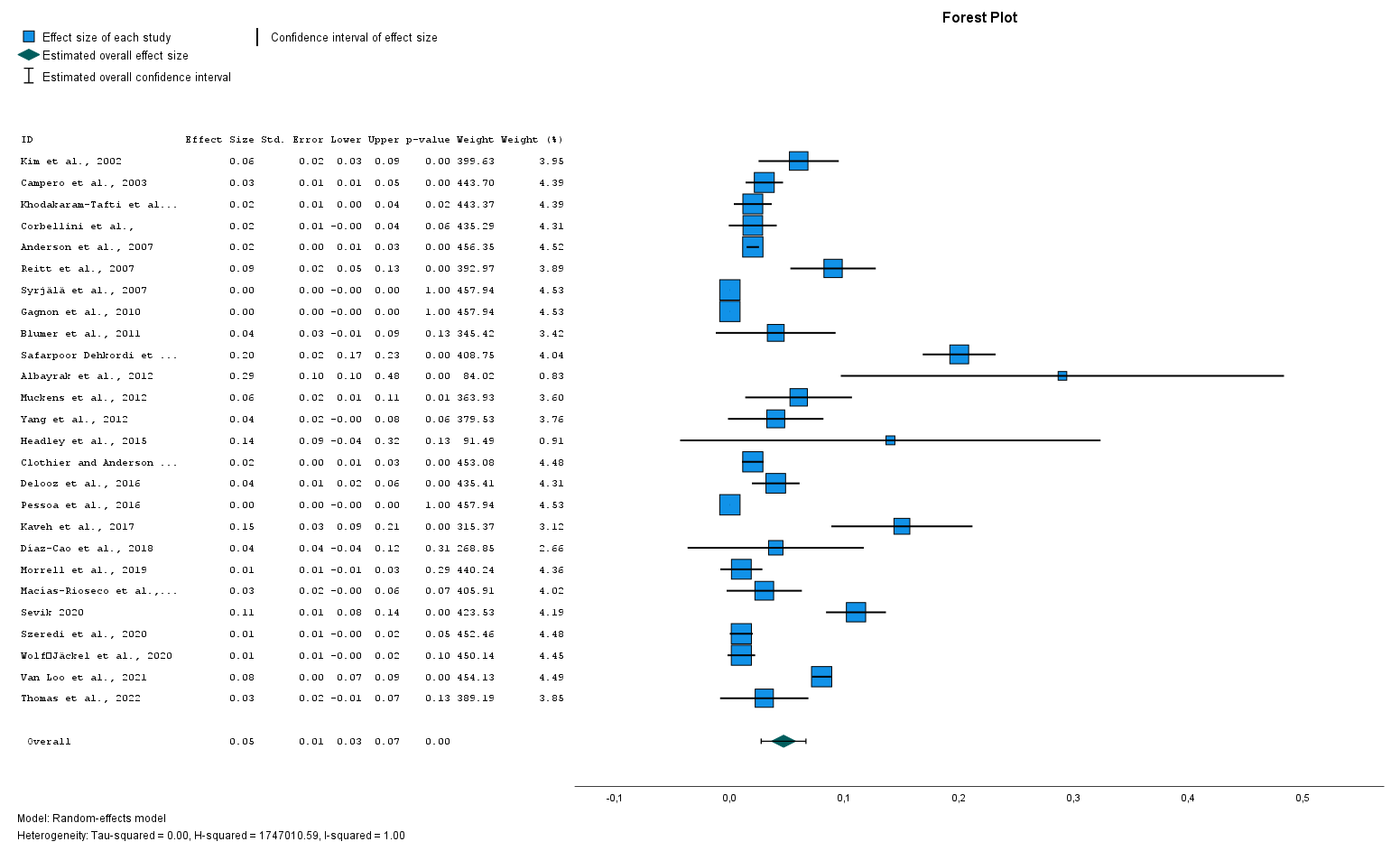


**Supplementary Figure 3.** Forest plot of the results of the subgroup analysis for each agent of bovine abortion. The blue square is the point estimate, and the horizontal line is the 95% confidence interval (CI) for prevalence plotted for each dataset. The left columns show the bibliographic reference for each dataset, the prevalence, the standard error, 95% CI from each dataset, the *p* value and the weight of the study related to the global estimate. The green diamond at the bottom of the forest plot is a worldwide-pooled prevalence of each infectious agents related to bovine abortion. (a) *Neospora caninum*; (b) Opportunistic bacteria; (c) Chlamydiaceae family; (d) *Coxiella burnetii*; (e) *Leptospira* spp.; (f) *Brucella* spp.; (g) *Campylobacter* spp.; (h) *Listeria* spp. (i) *Tritrichomonas foetus*; (j) Fungus; (k) Bovine Herpes Virus type 1; (l)Bovine Viral Diarrhoea. CI: confidence interval. I^2^: heterogeneity. NA: not available. I: isolate/culture. HP: histopathology; IHC: immunohistochemistry; FS: foetal serology; EAg: detection of antigen by direct ELISA; SS: special staining; DFAT: direct fluorescence antibody test, SN: seroneutralization.

(a)


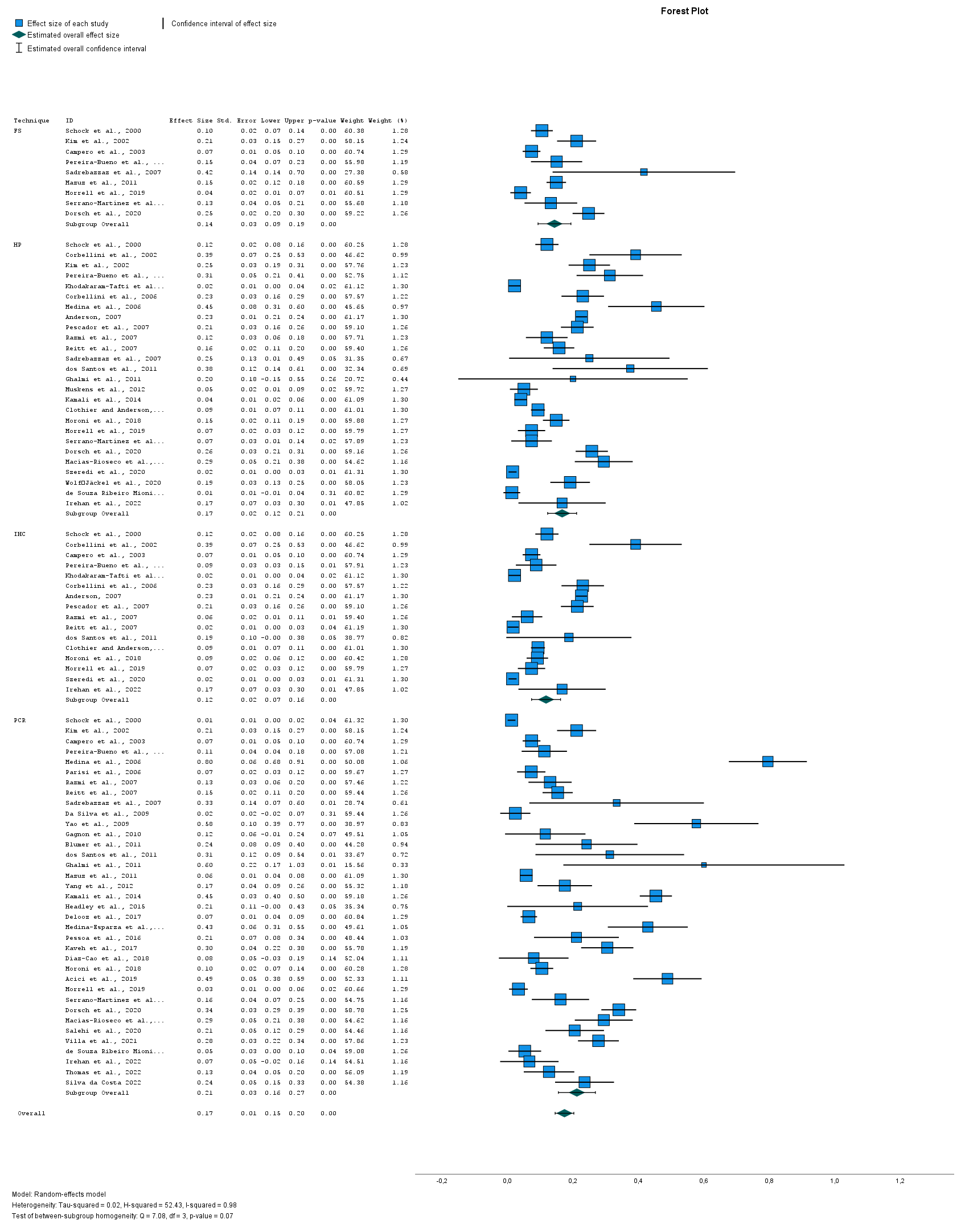


(b)


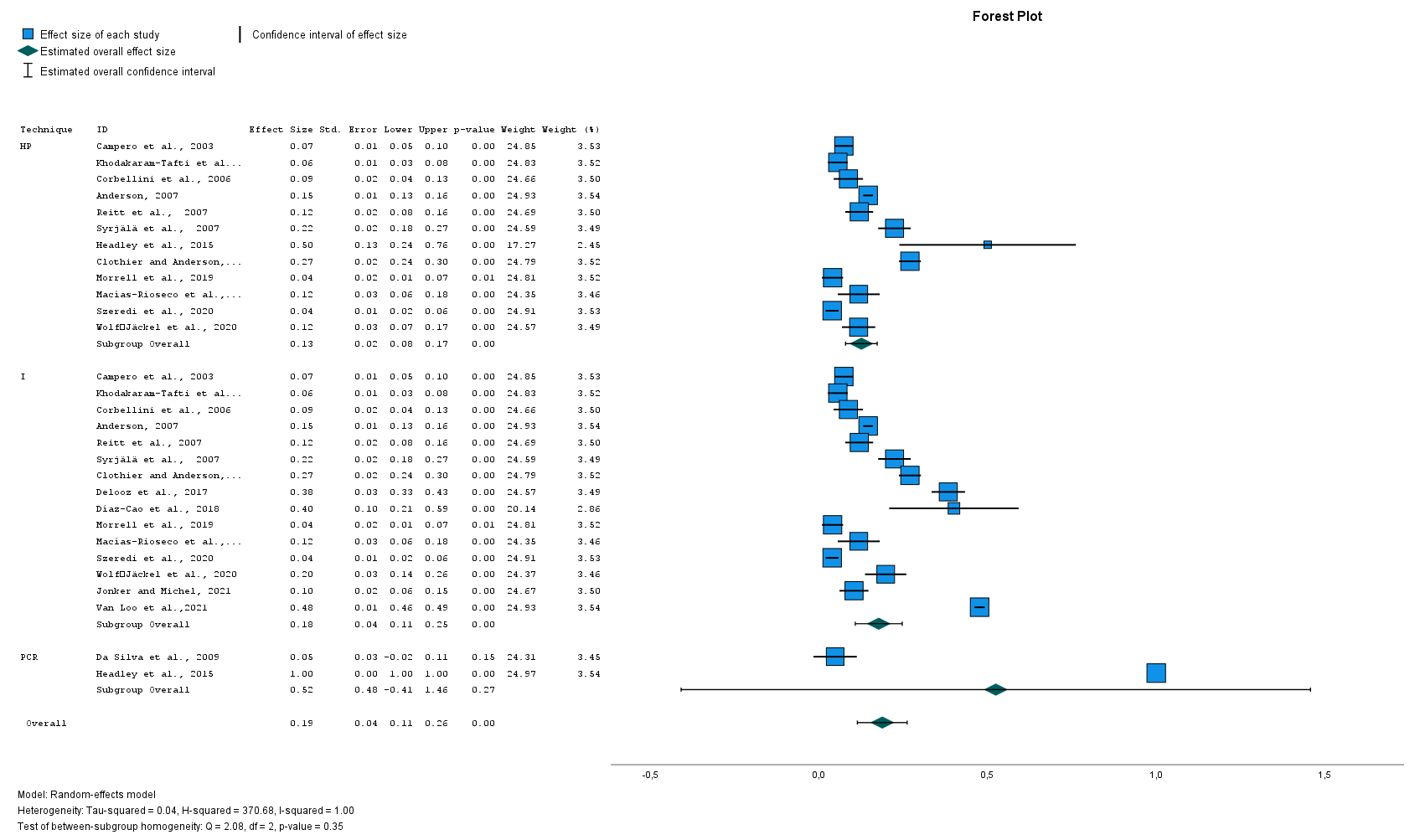


(c)


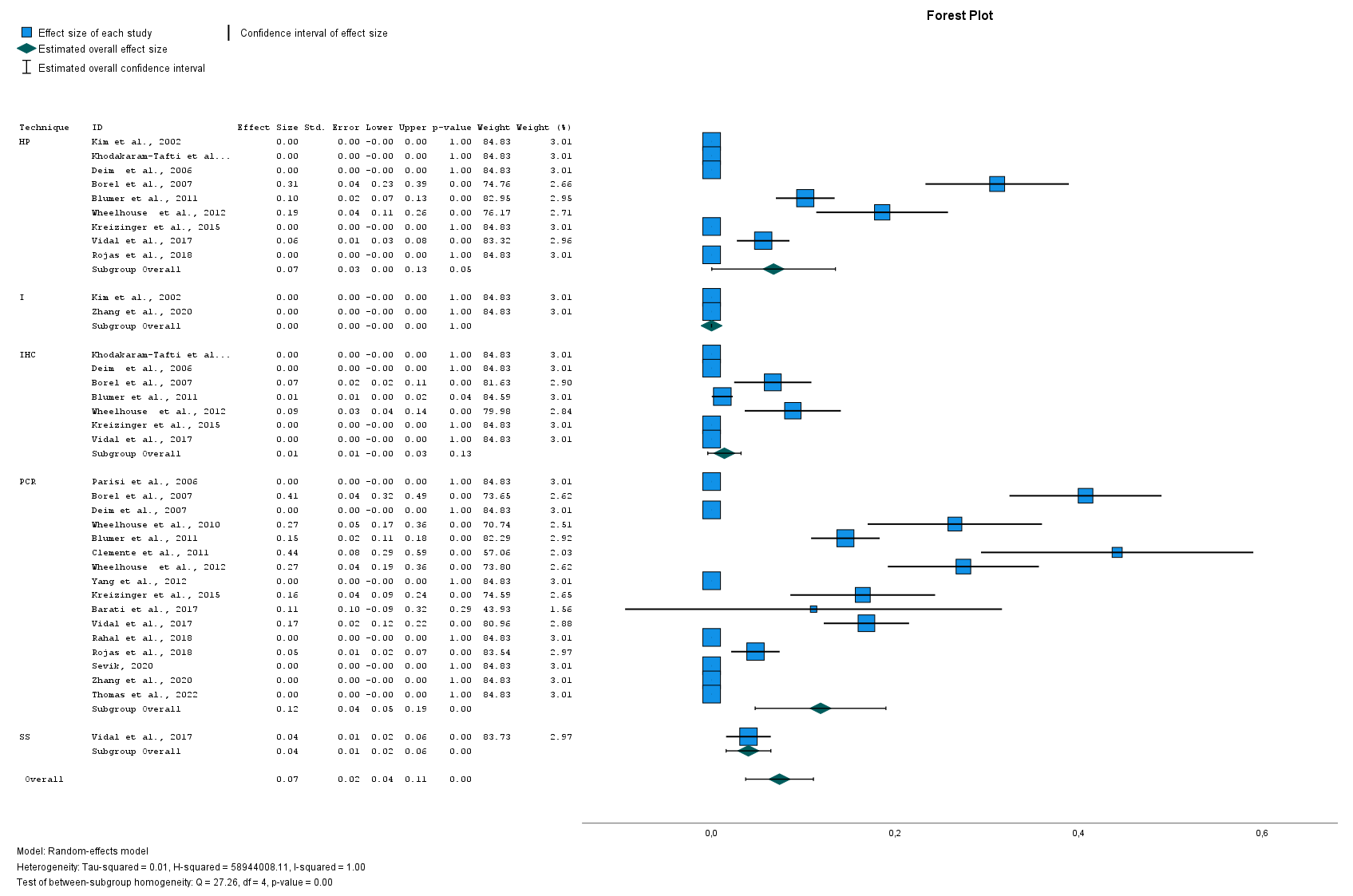


(d)


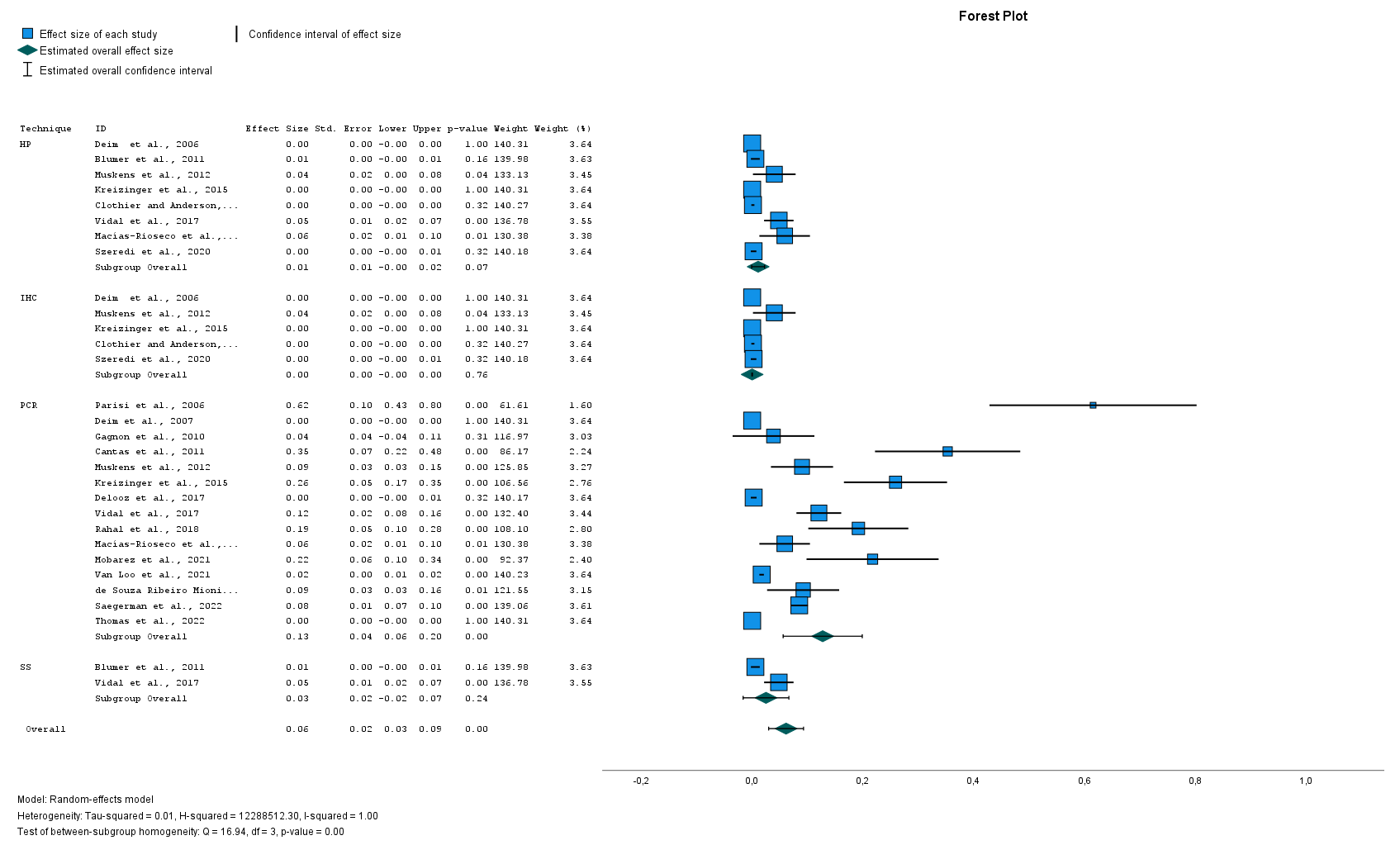


(e)

**
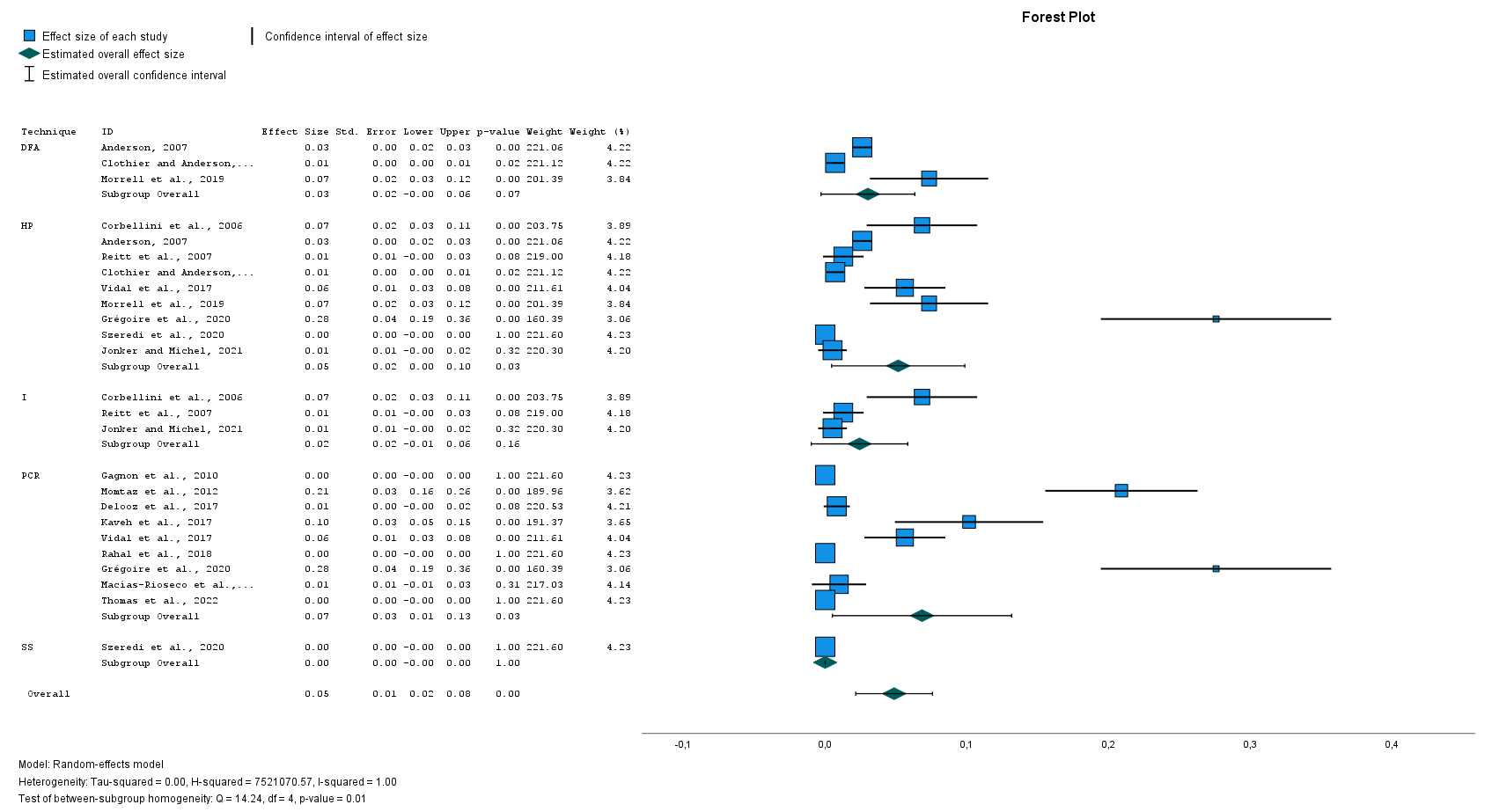
**

(f)


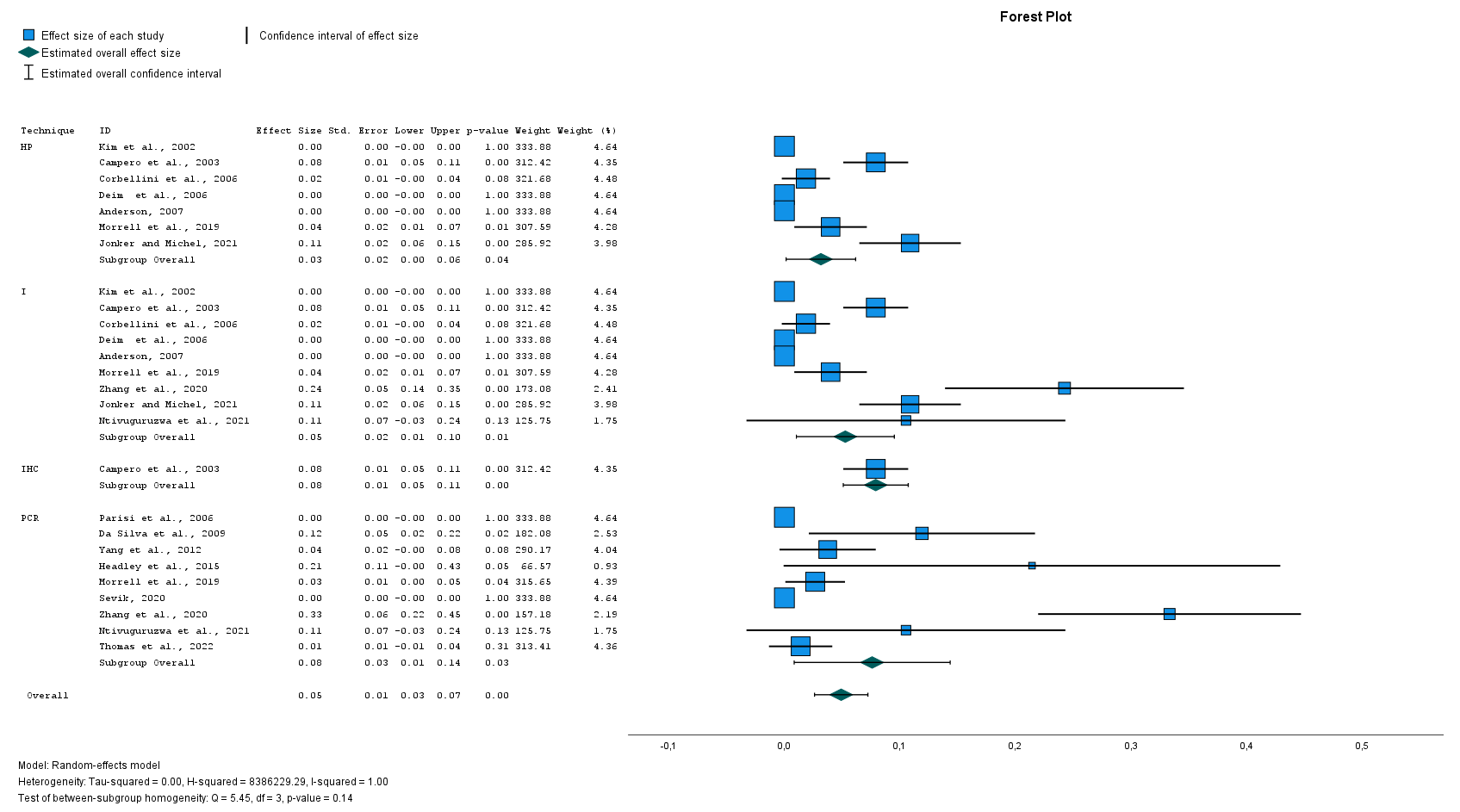


(g)


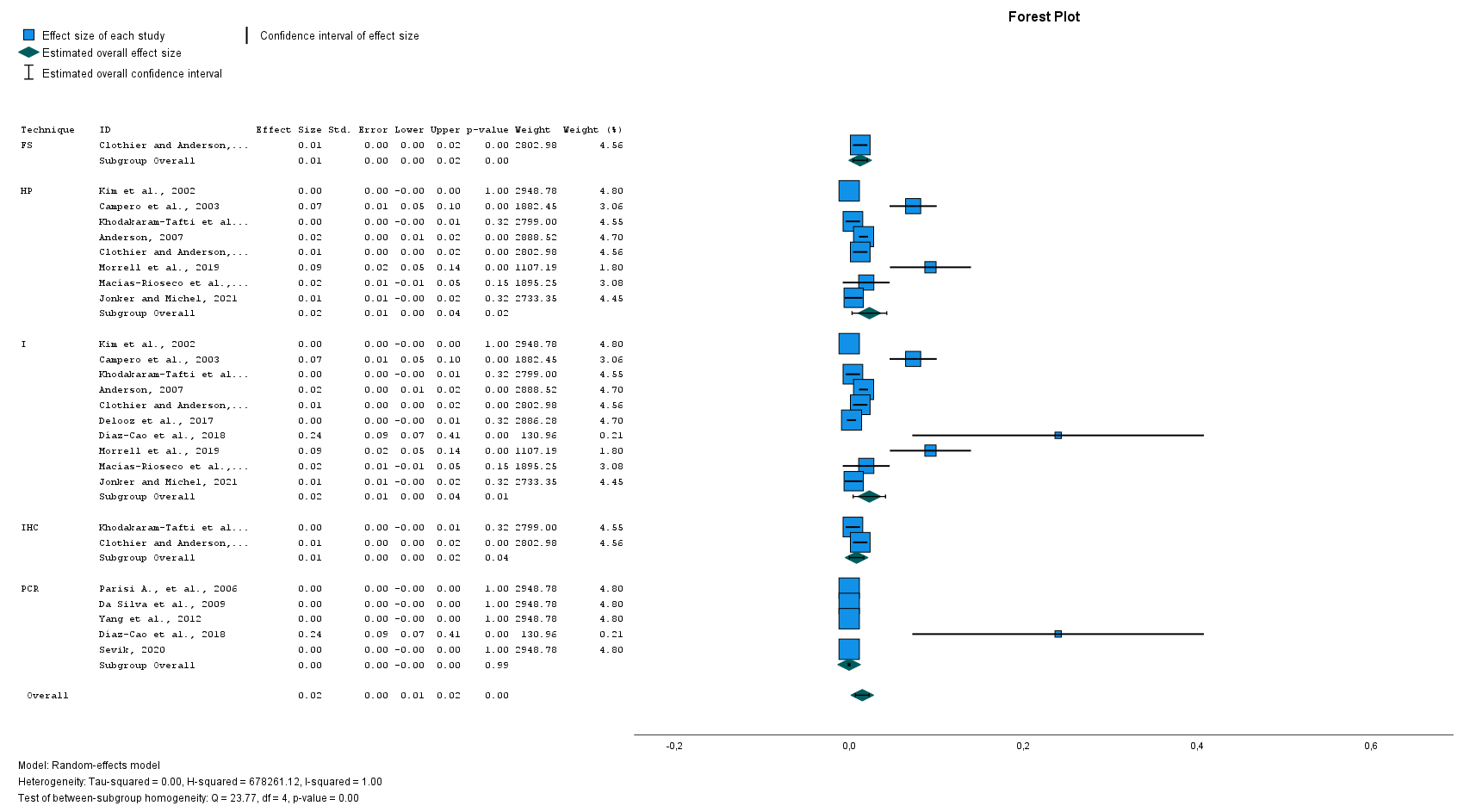


(h)


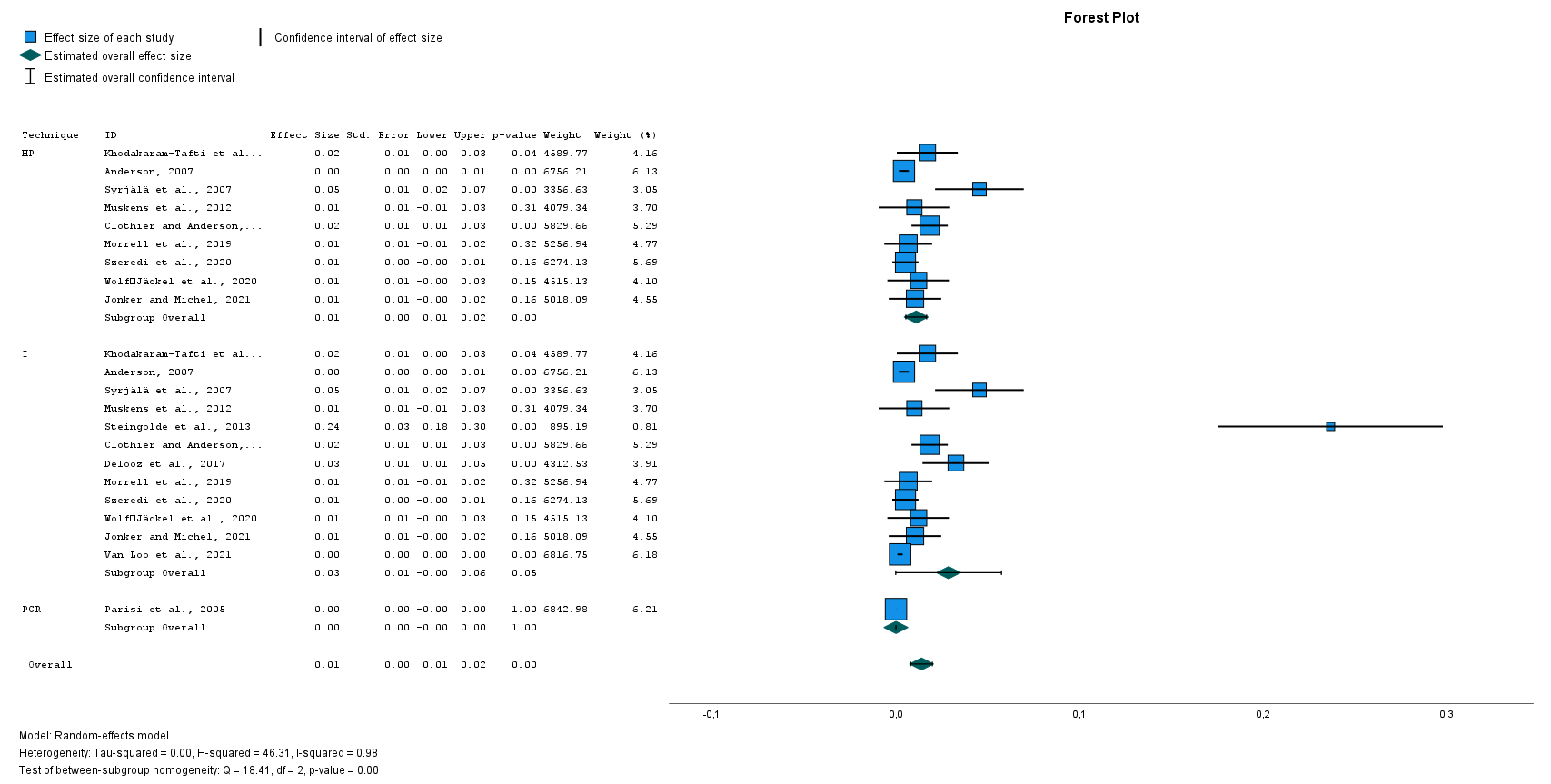


(i)


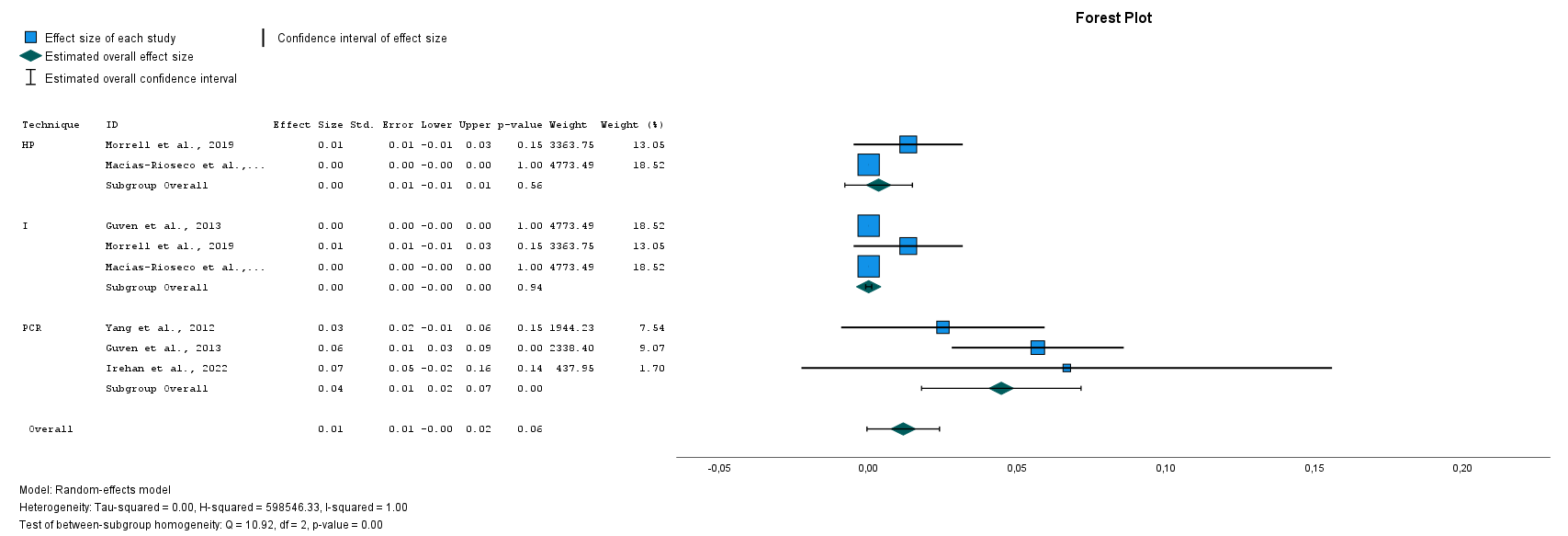


(j)


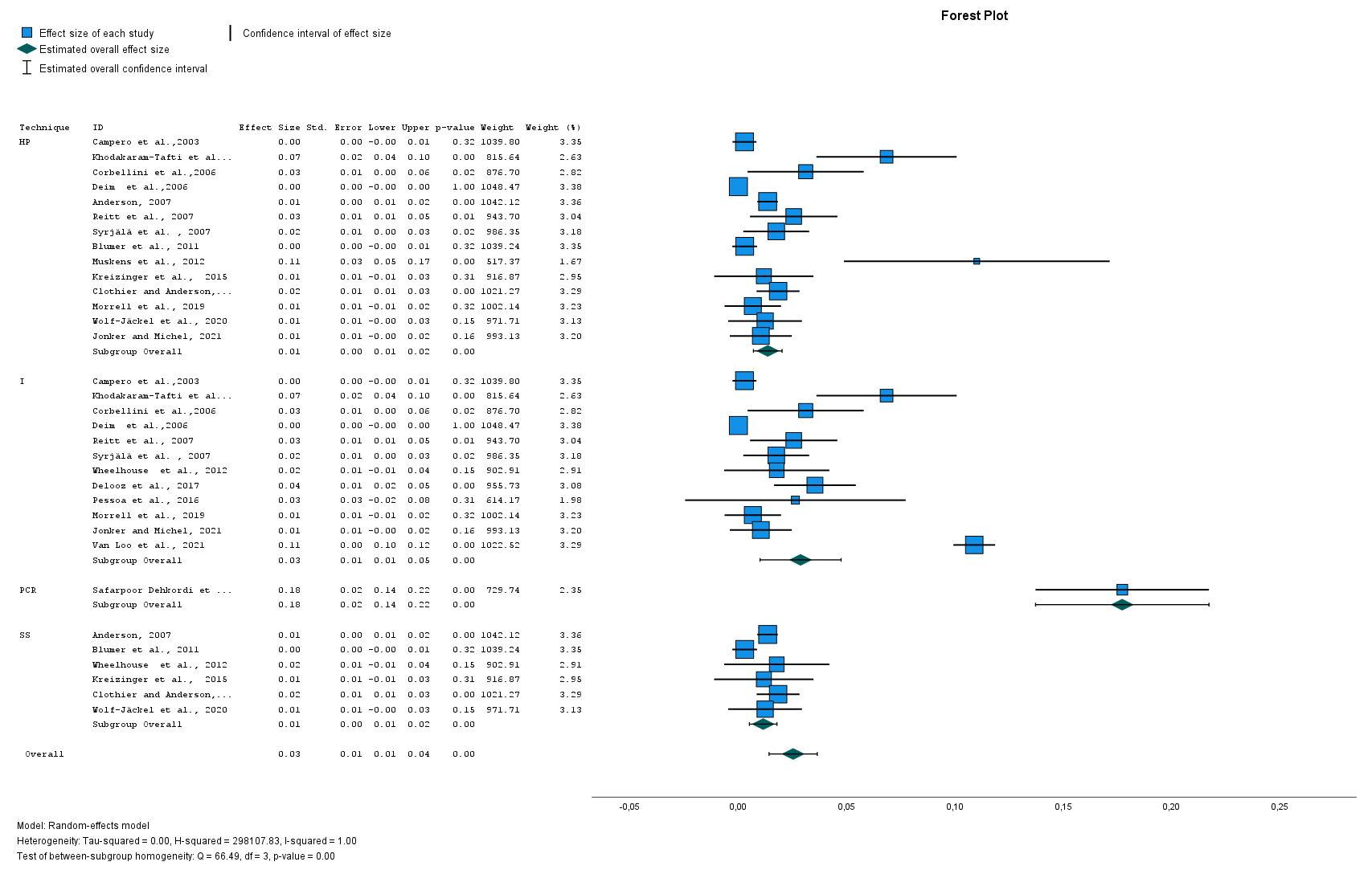


(k)


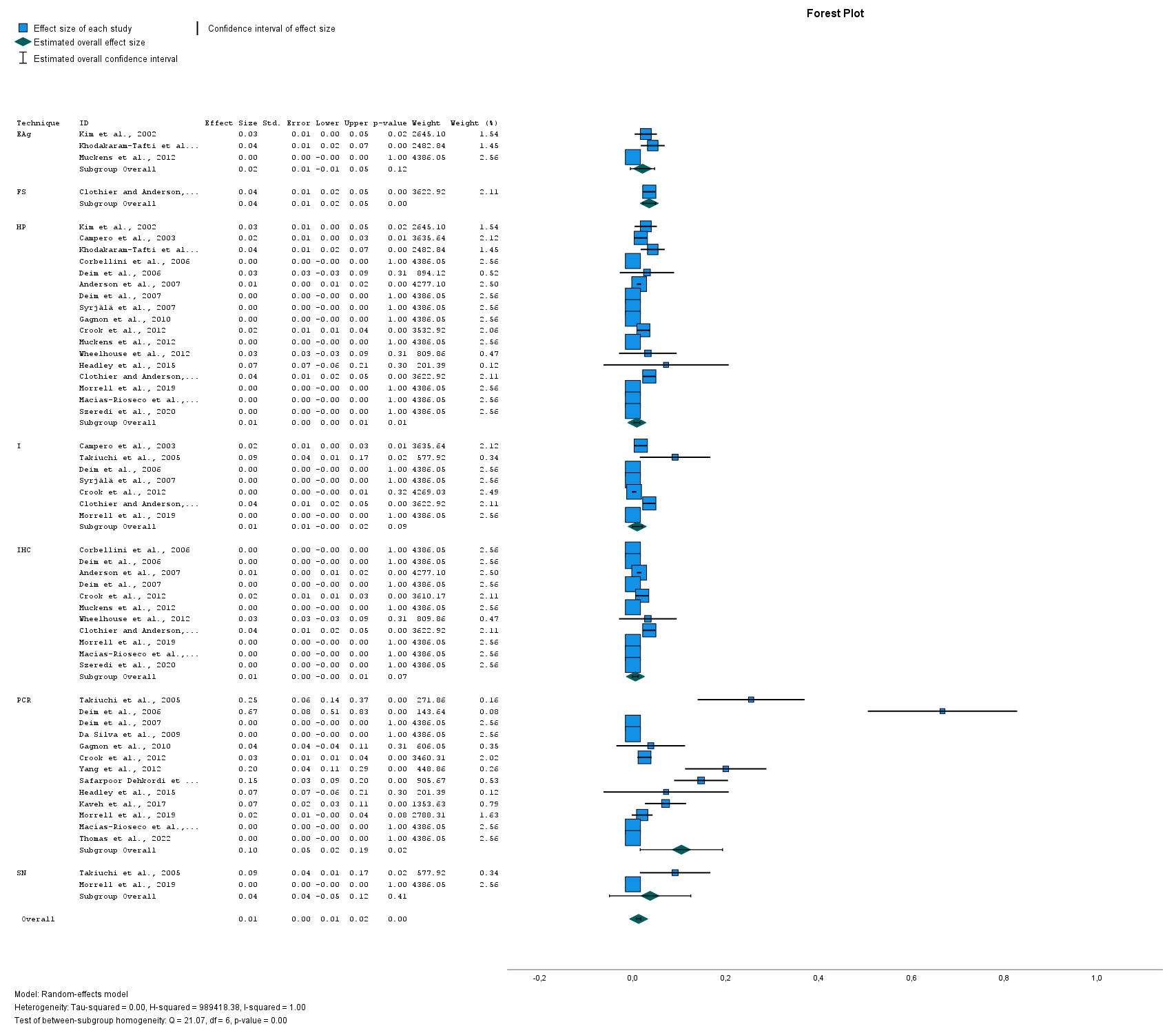


(l)


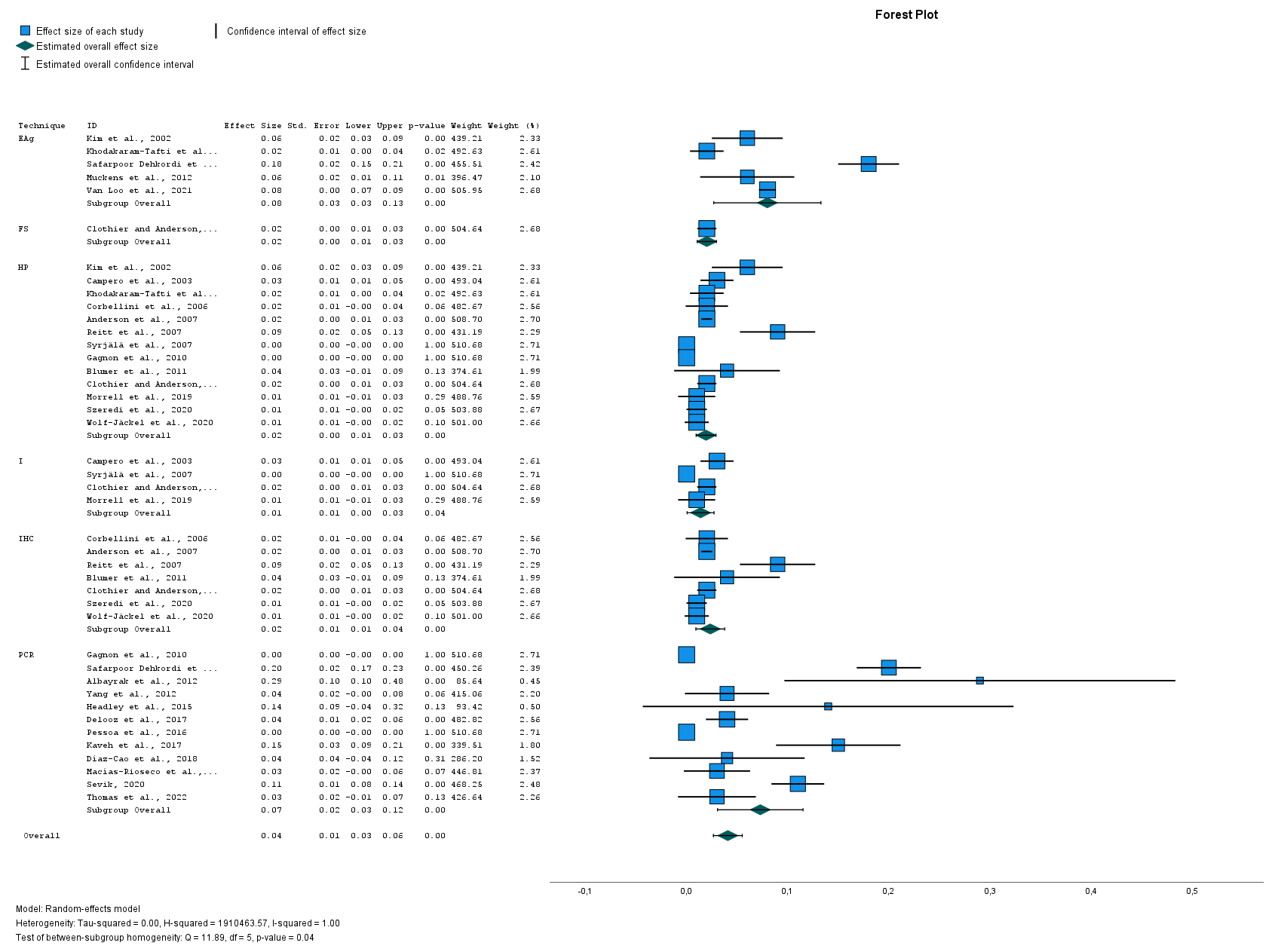


**Supplementary Figure 4.** Forest plot of the results of the leave-one-out cross-validation (LOOCV) for each agent of bovine abortion. The black square is the point estimate, and the horizontal line is the 95% confidence interval (CI) for prevalence plotted for each dataset. The left columns show the bibliographic reference, the prevalence and the 95% CI from each dataset. The blue diamond at the bottom of the forest plot is a worldwide prevalence of each infectious agents related to bovine abortion. (a) *Neospora caninum*; (b) Opportunistic bacteria; (c) Chlamydiaceae family; (d) *Coxiella burnetii*; (e) *Leptospira* spp.; (f) *Brucella* spp.; (g) *Campylobacter* spp.; (h) *Listeria* spp. (i) *Tritrichomonas foetus*; (j) Fungus; (k) Bovine Herpes Virus type 1; (l)Bovine Viral Diarrhoea.

(a)


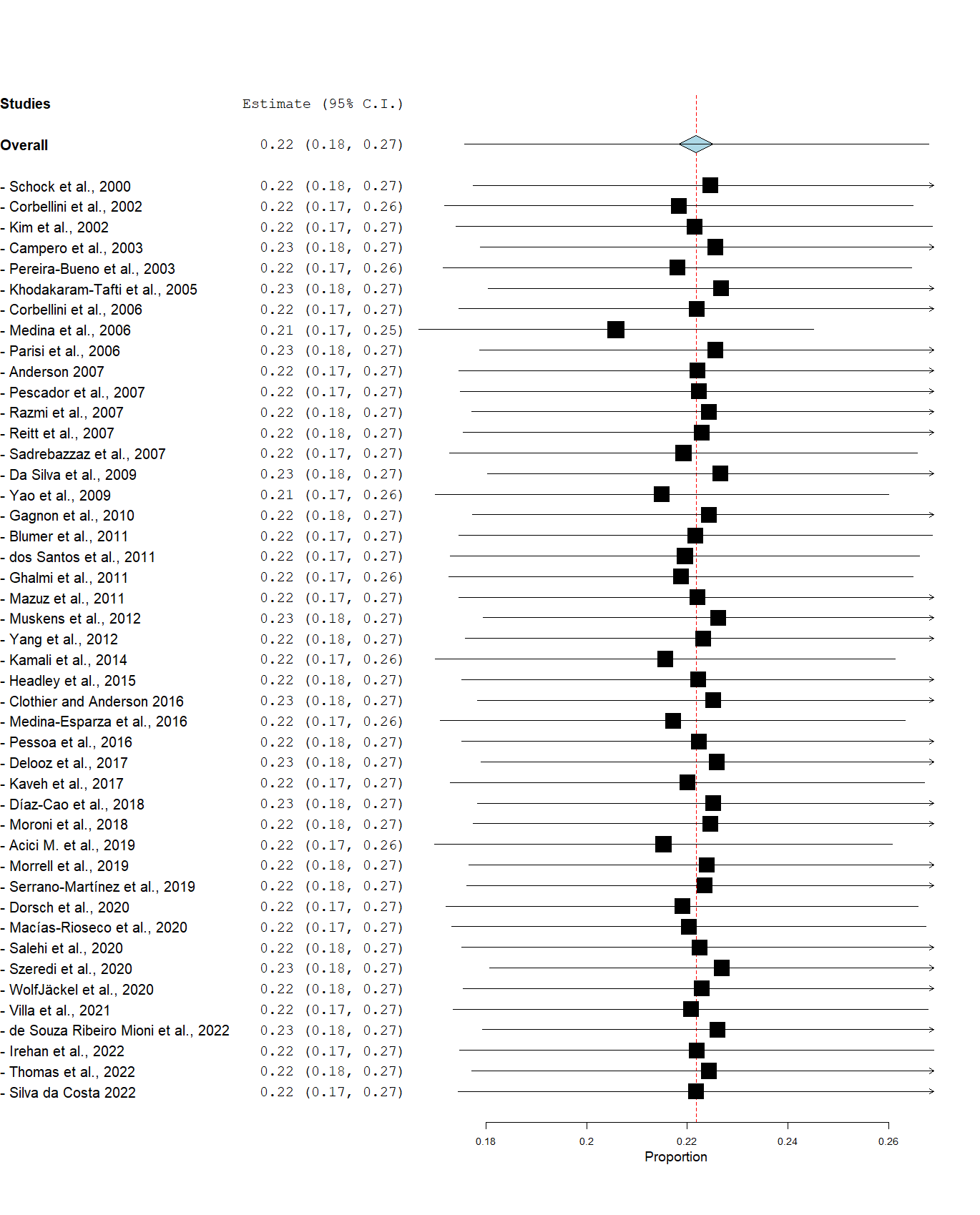


(b)


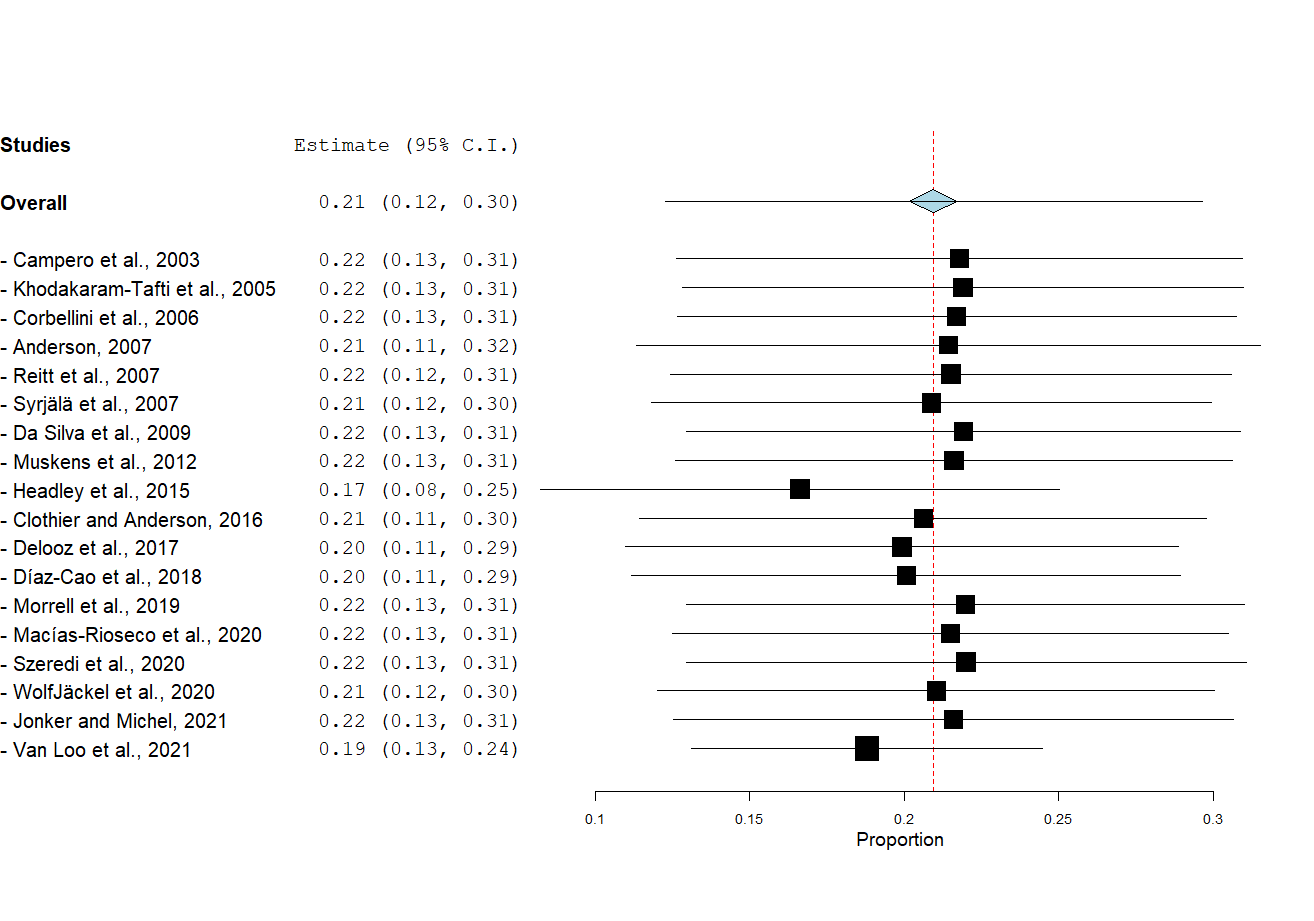


(c)


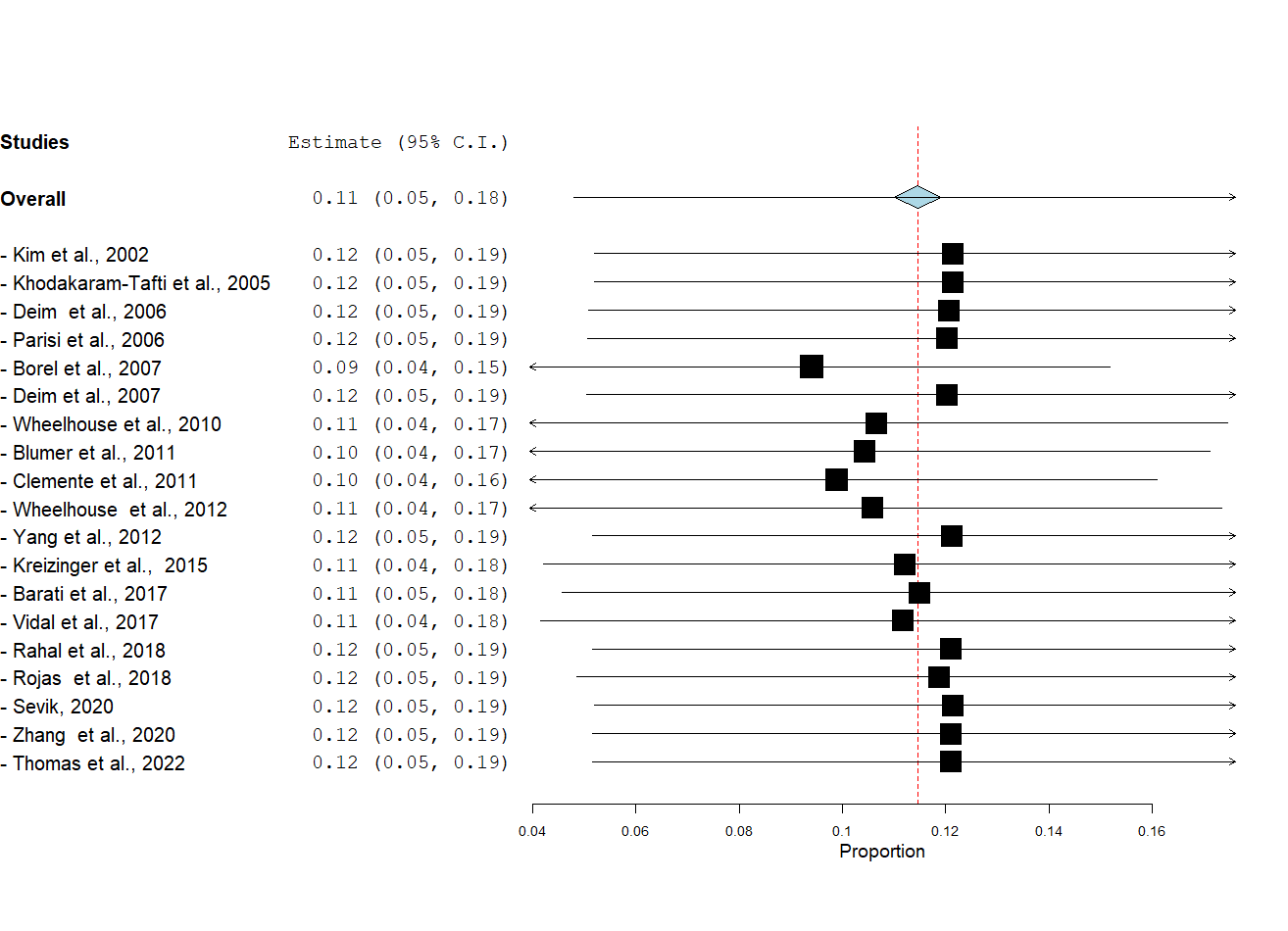


(d)


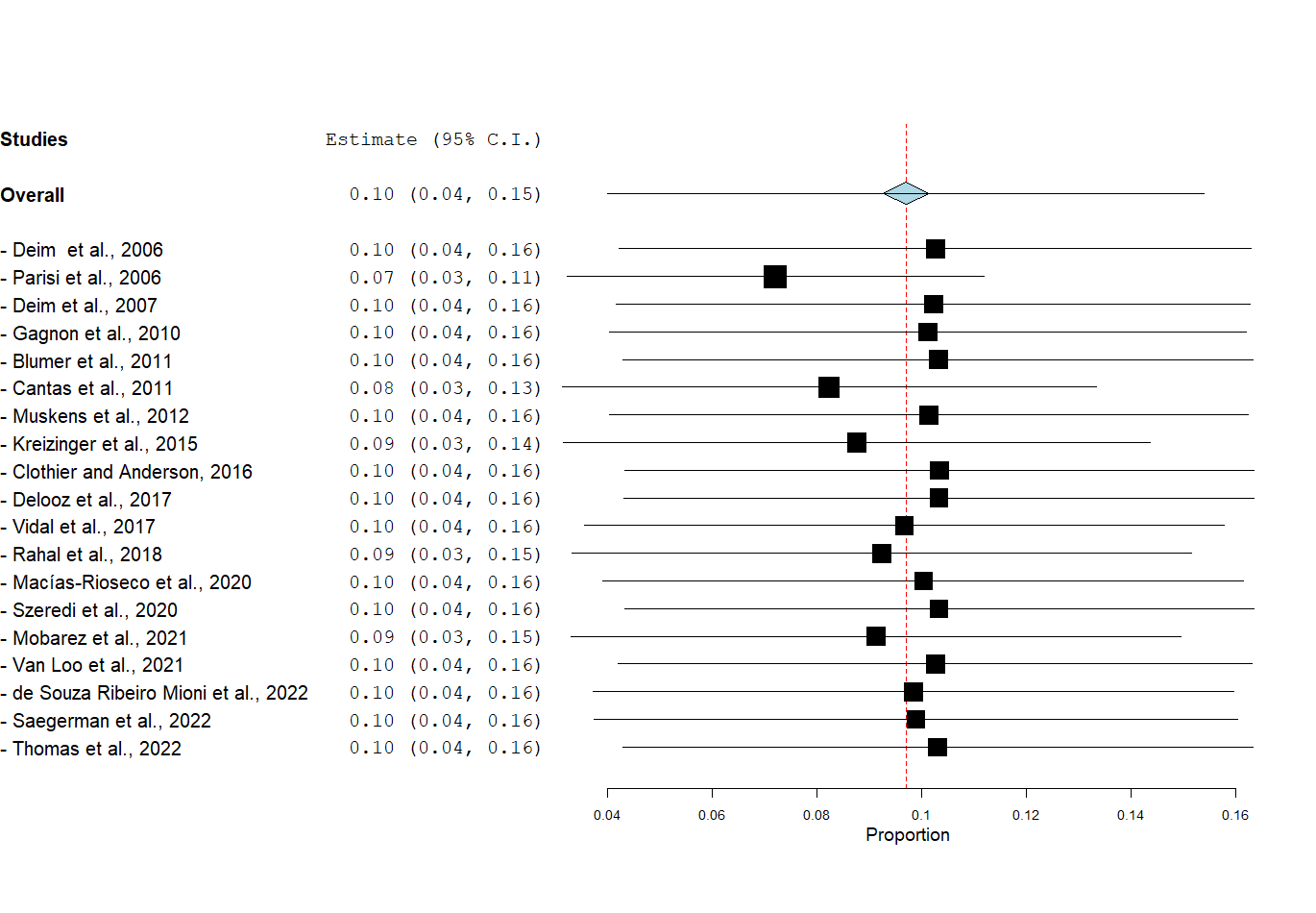


(e)


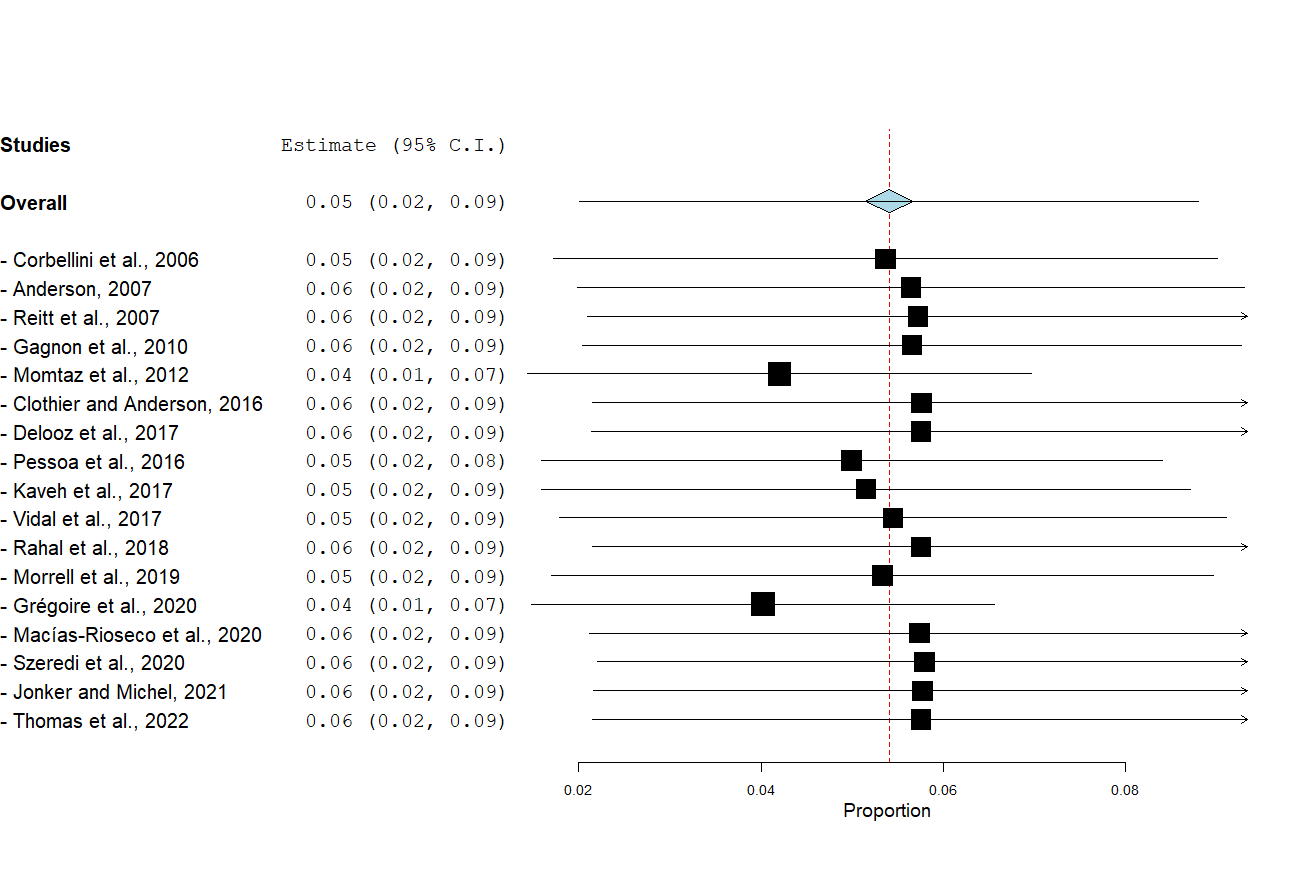


(f)


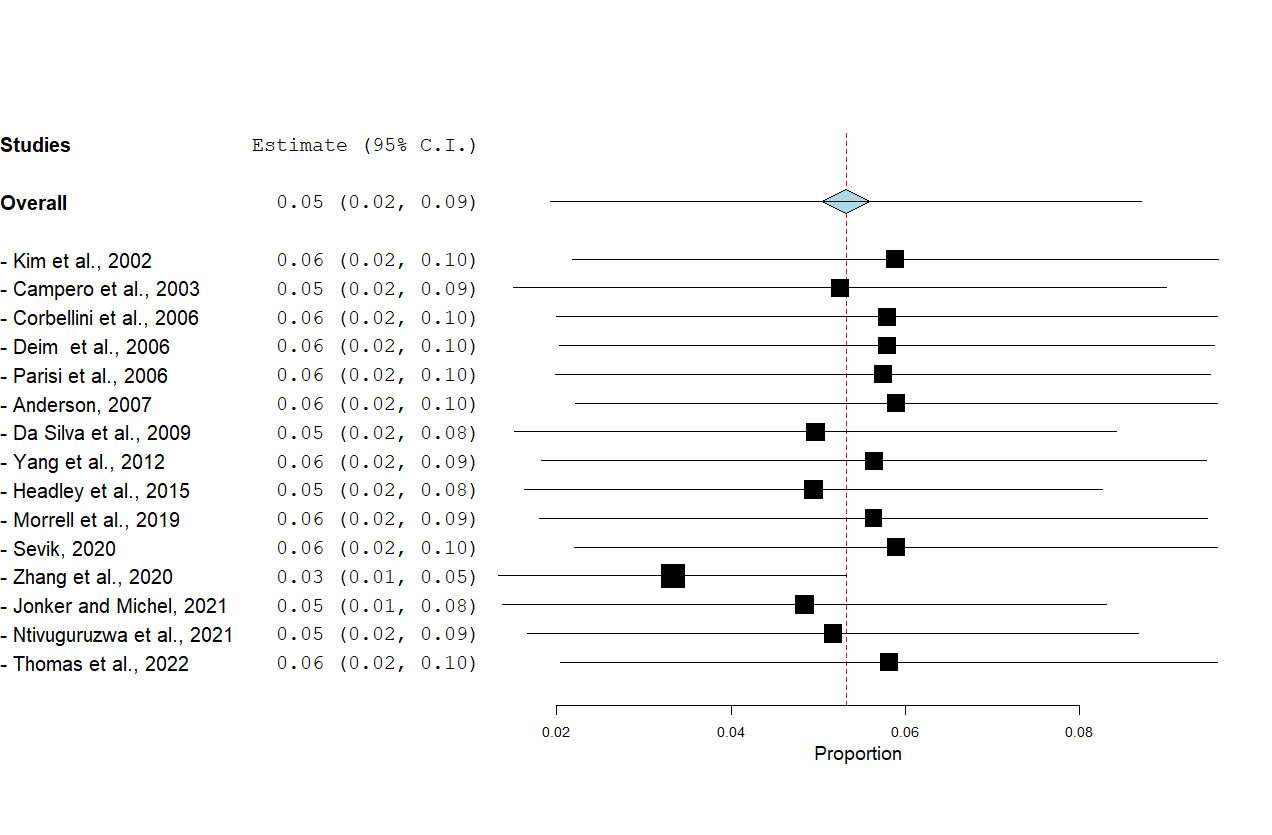


(g)


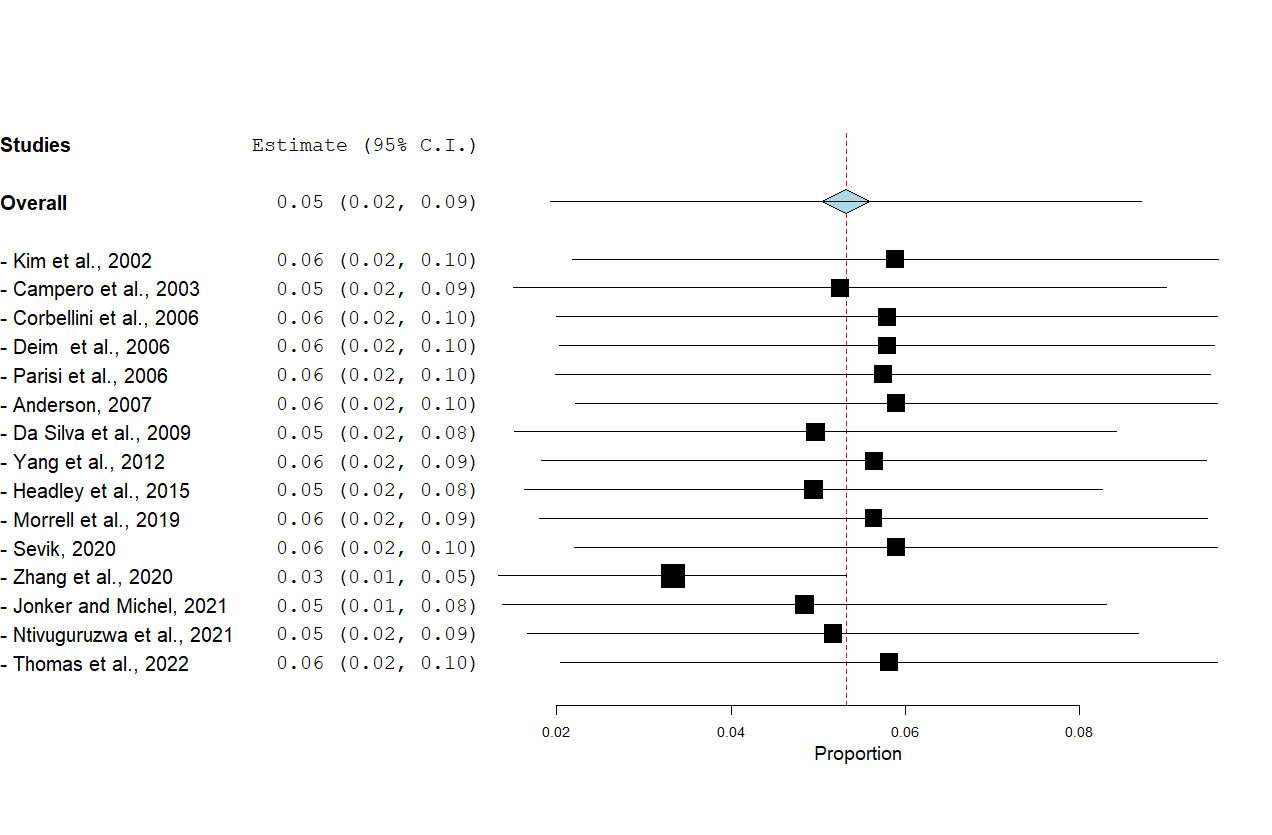


(h)


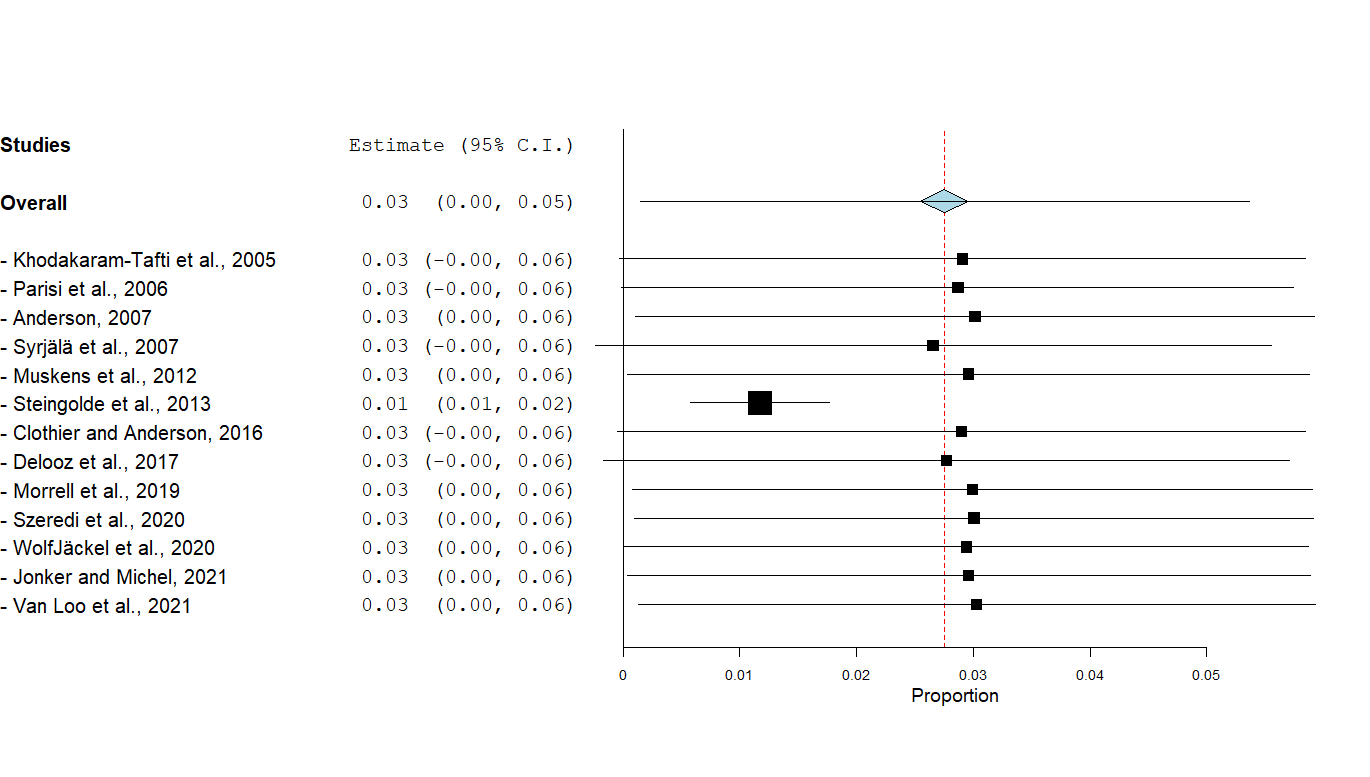


(i)


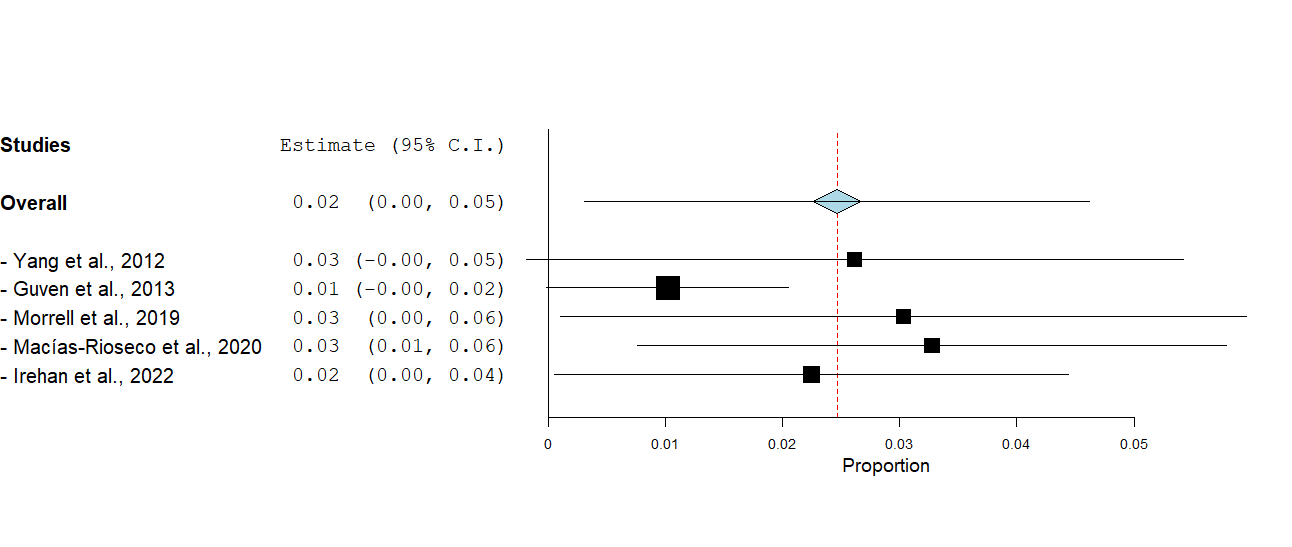


(j)


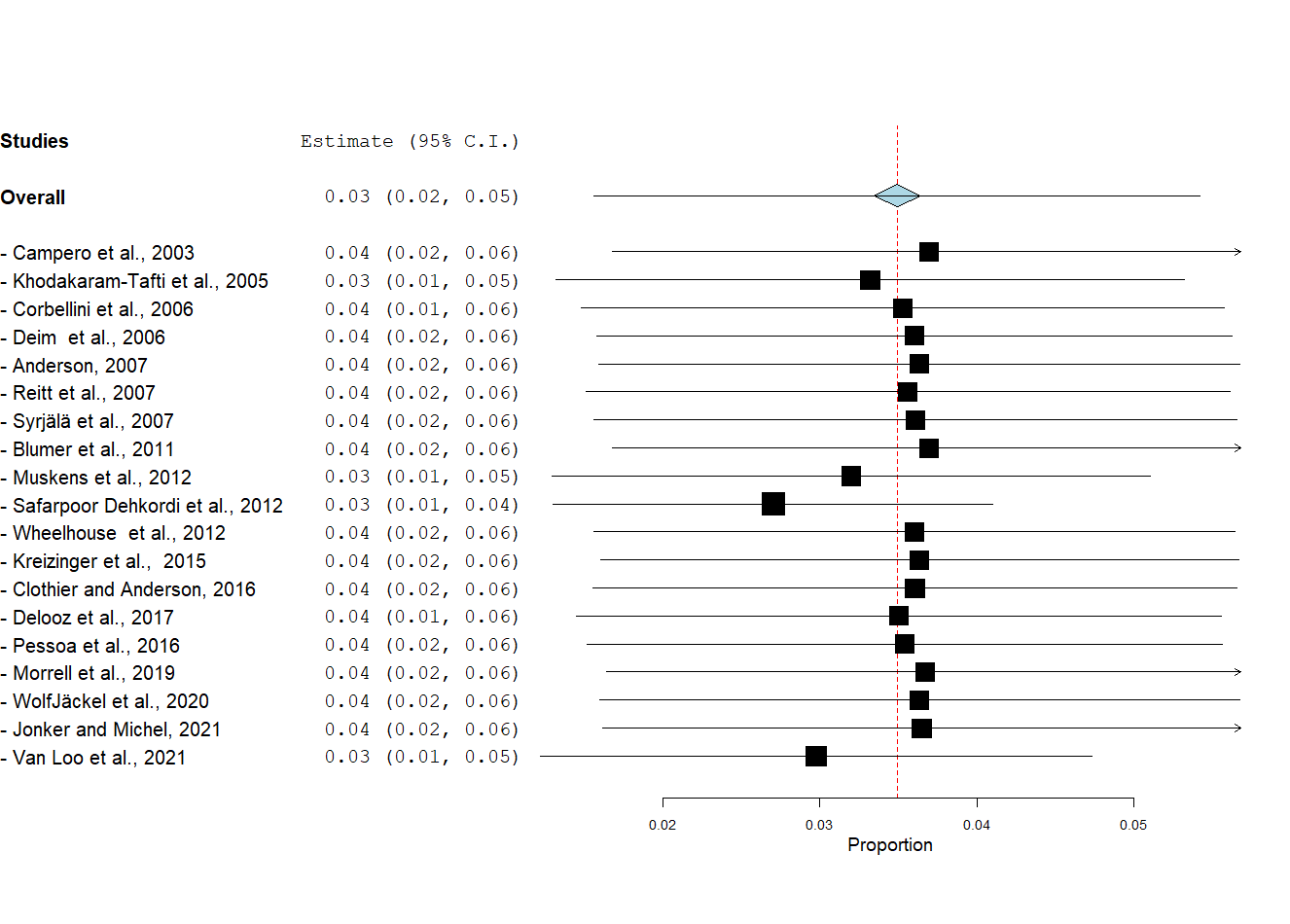


(k)


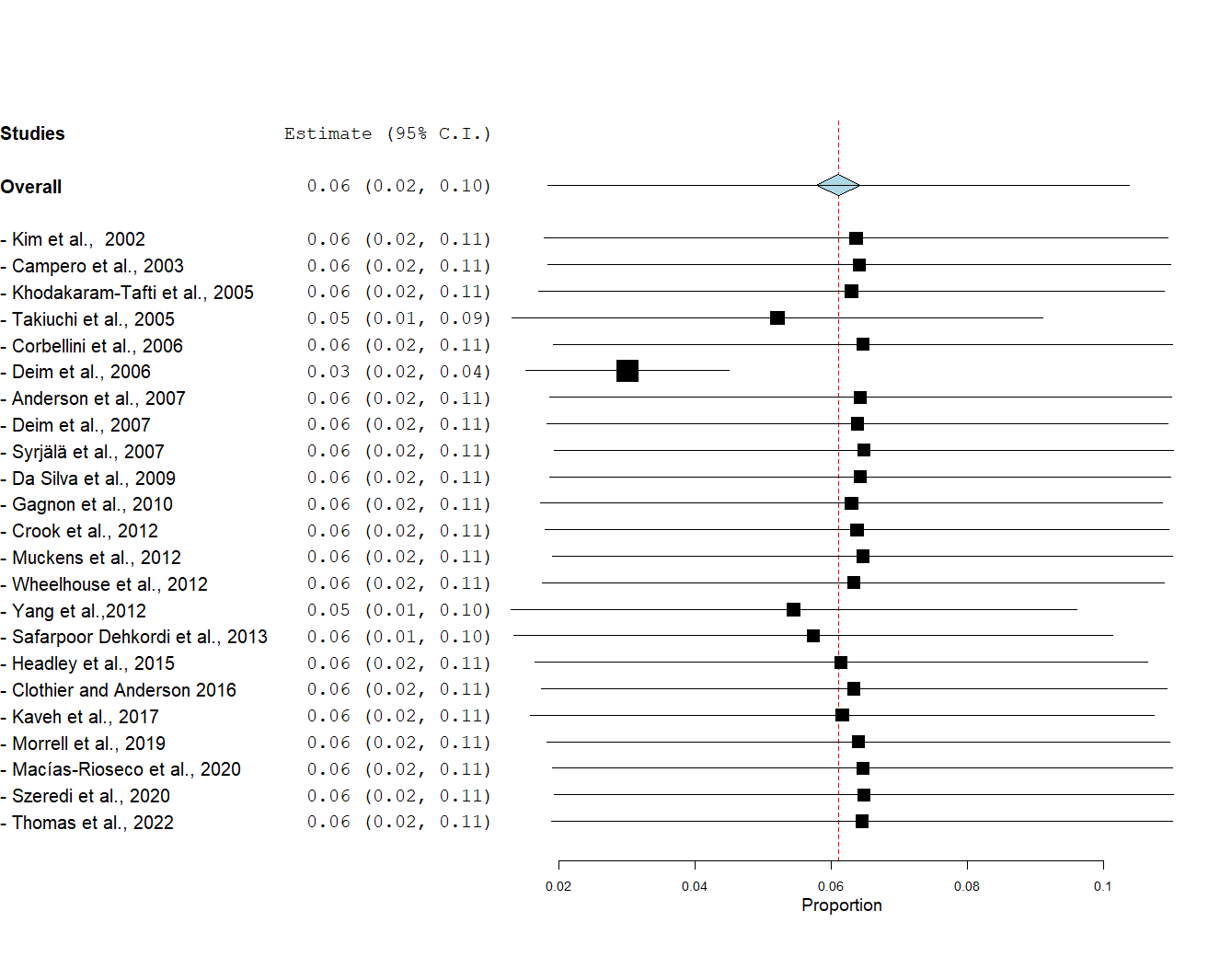


(l)


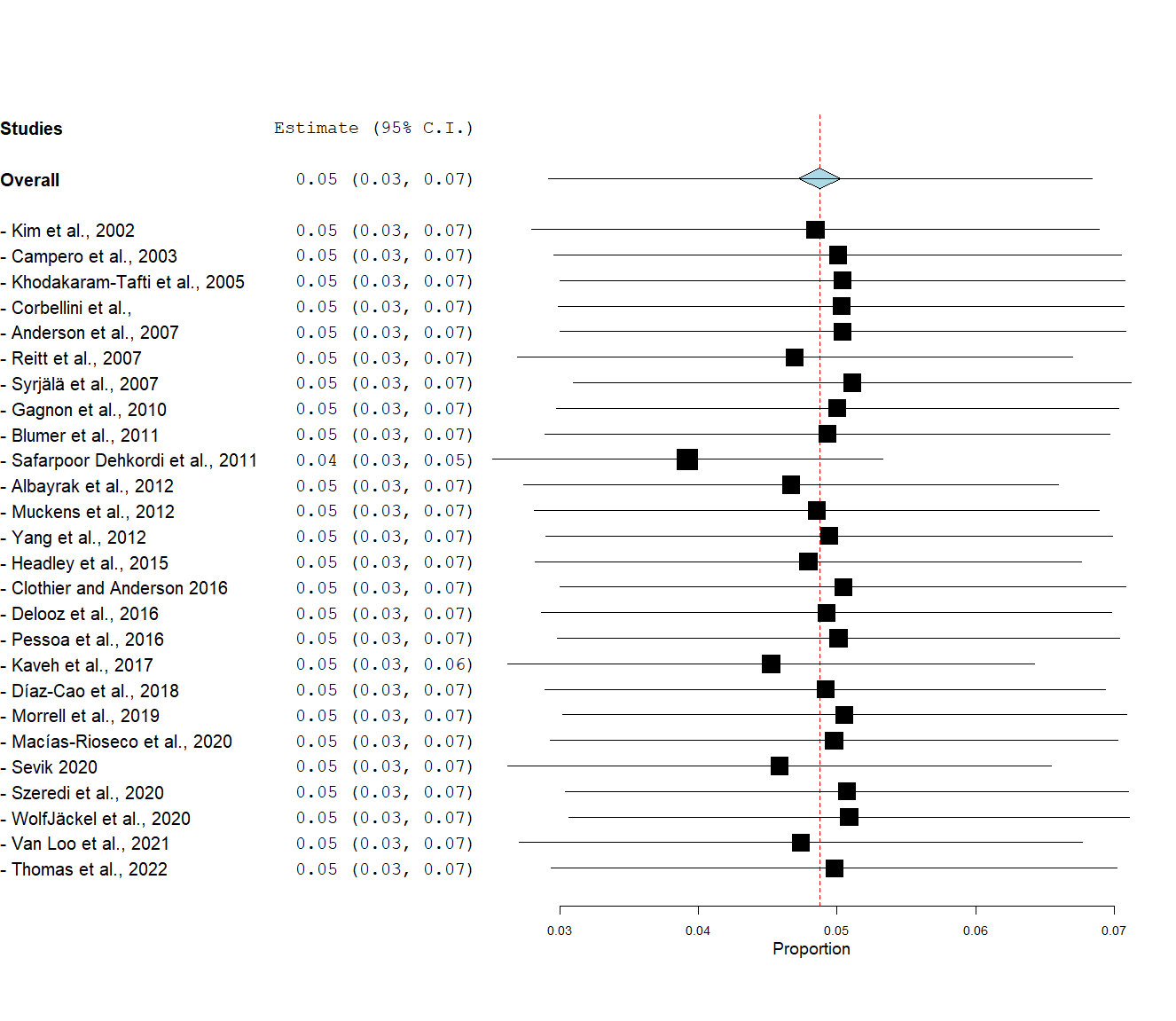


**Supplementary Figure 5.** Bubble plot of the results of the meta-regression of year of published articles against the prevalence of each infectious agent. The circles represent the individual studies. The continuous lines represent the regression lines. The year of publication is plotted on the horizontal axis. The prevalence of each each infectious agent is plotted on the vertical axis. (a) *Neospora caninum*; (b) Opportunistic bacteria; (c) Chlamydiaceae family; (d) *Coxiella burnetii*; (e) *Leptospira* spp.; (f) *Brucella* spp.; (g) *Campylobacter* spp.; (h) *Listeria* spp. (i) *Tritrichomonas foetus*; (j) Fungus; (k) Bovine Herpes Virus type 1; (l)Bovine Viral Diarrhoea.

(a)


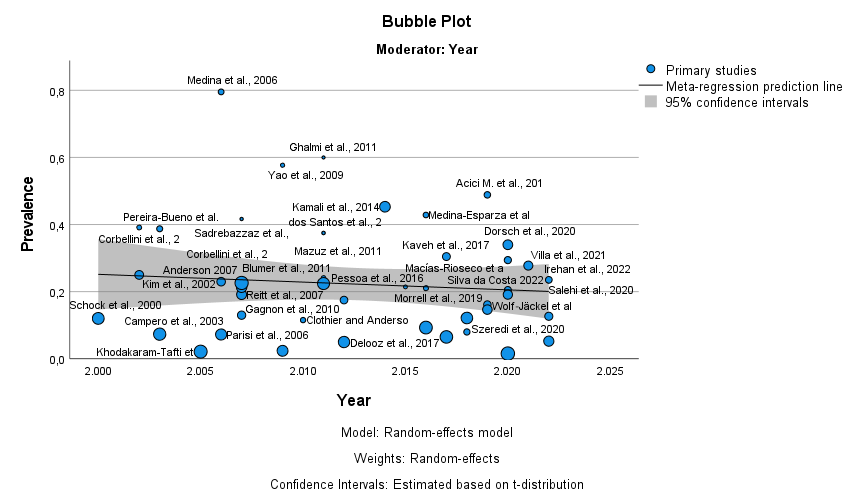


(b)


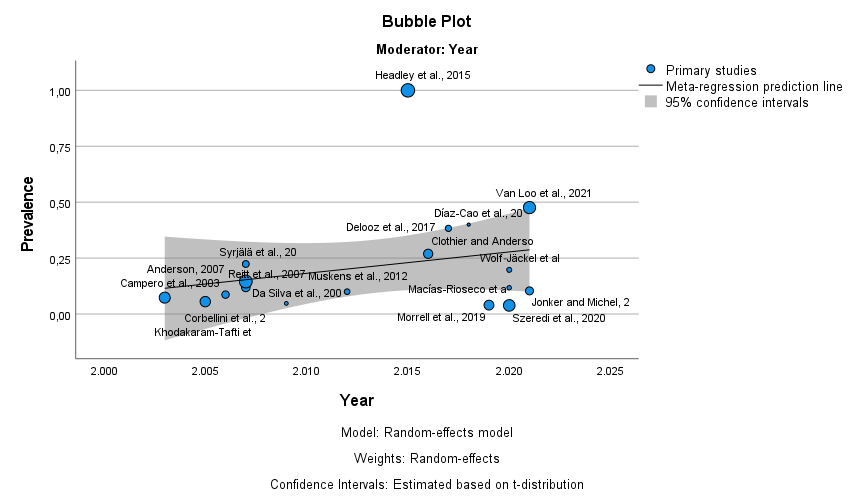


(c)


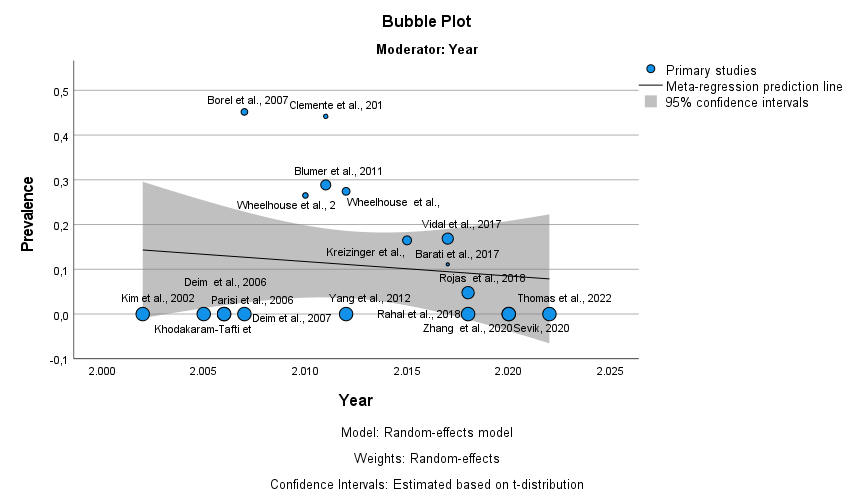


(d)


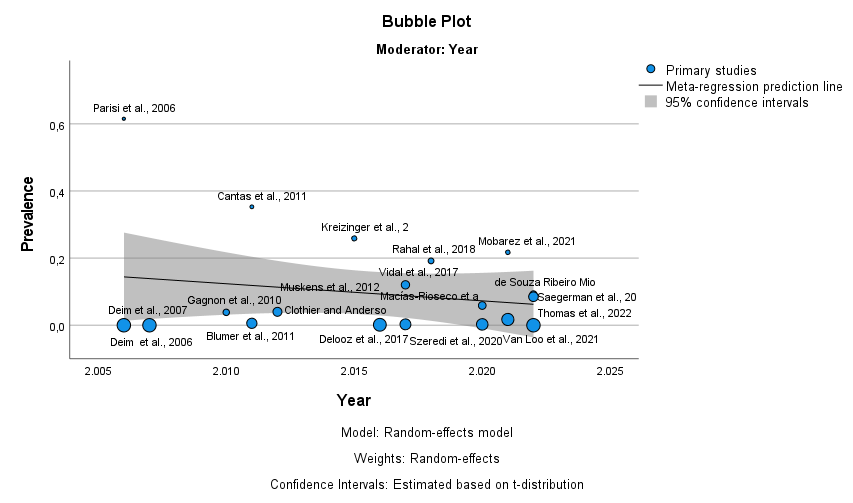


(e)


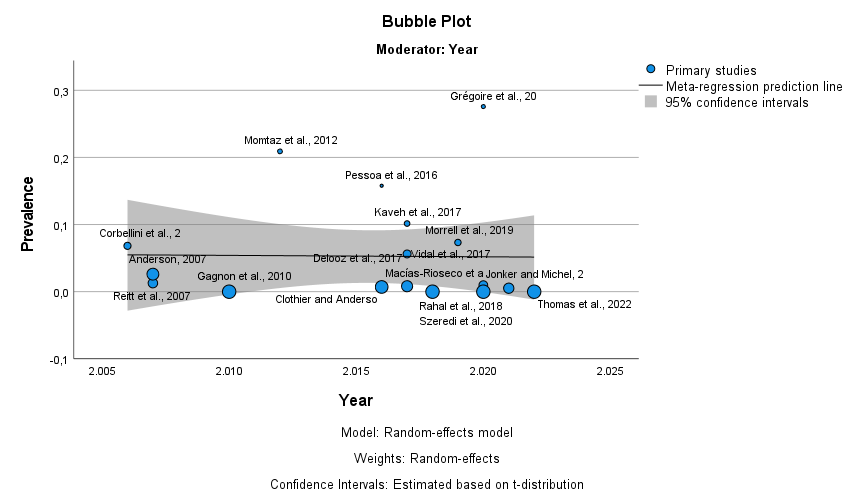


(f)


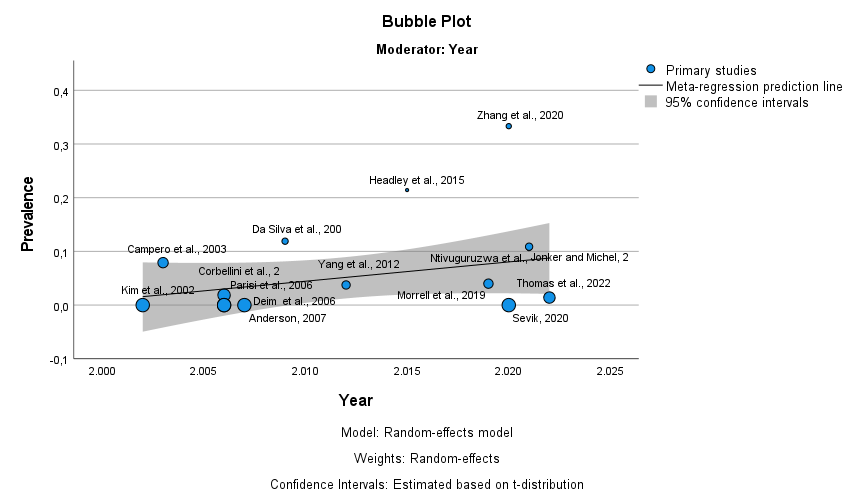


(g)


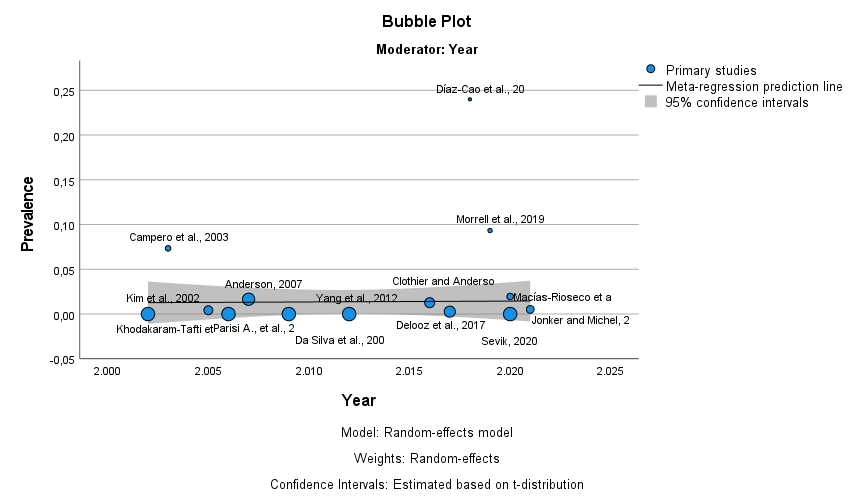


(h)


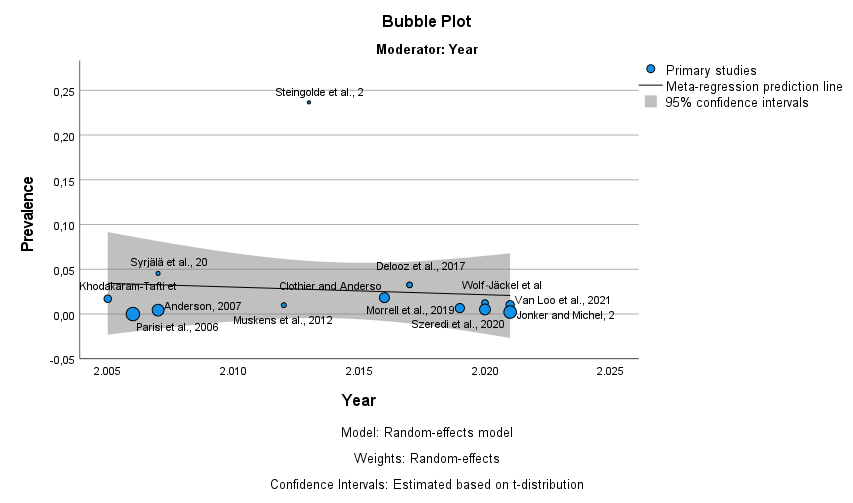


(i)


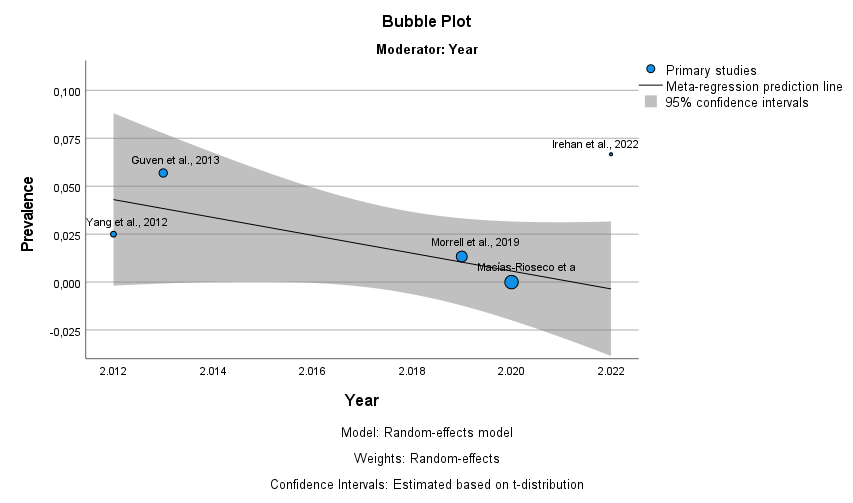


(j)


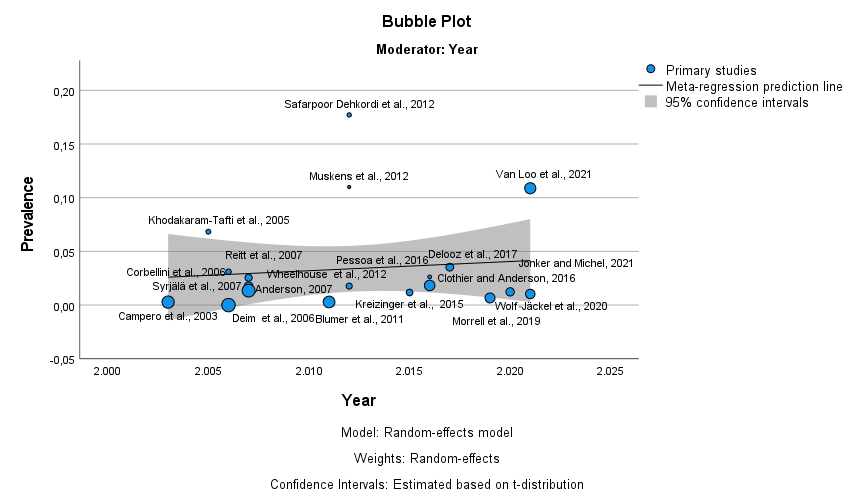


(k)


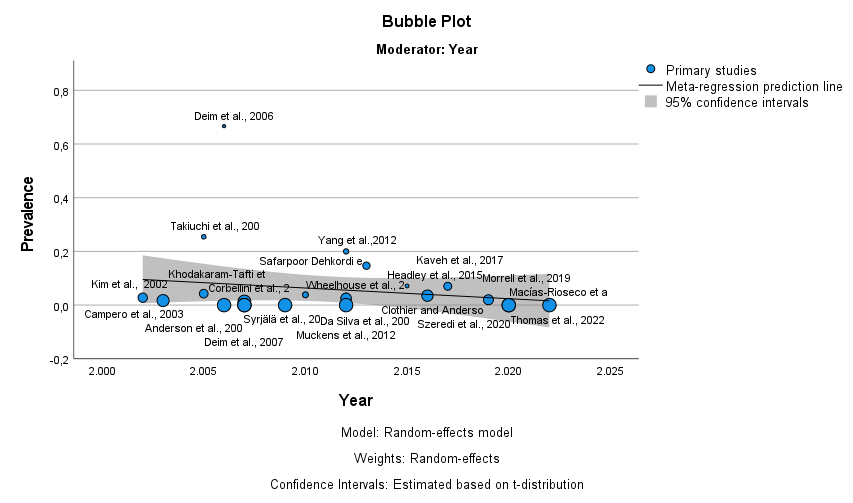


(l)


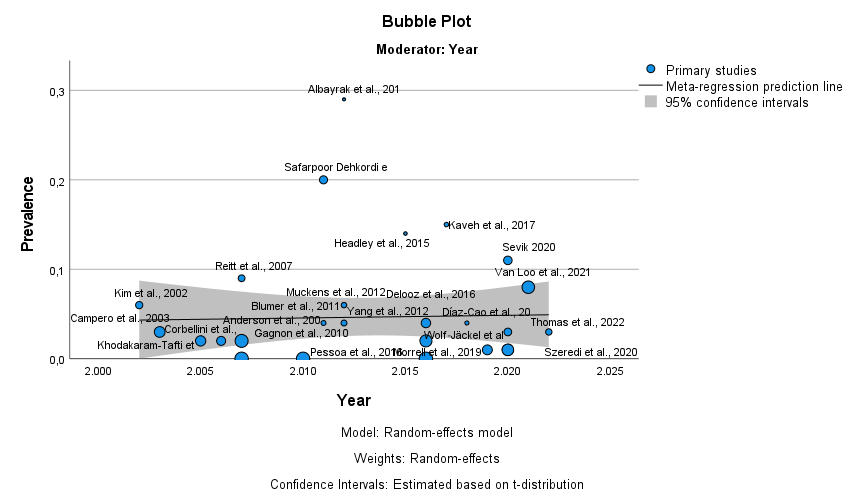

Supplement: Supplementary file 1 [file Data_Sheet_1.docx]
